# The prevalence and impact of psychiatric symptoms in an undiagnosed diseases clinical program

**DOI:** 10.1371/journal.pone.0216937

**Published:** 2019-06-06

**Authors:** Gabriella Waserstein, Clyde Partin, Debra Cohen, Pamela Schettler, Becky Kinkead, Mark Hyman Rapaport

**Affiliations:** 1 Emory University School of Medicine, Atlanta, Georgia, United States of America; 2 Emory Special Diagnostic Service Clinic, Department of Medicine, Emory University School of Medicine, Atlanta, Georgia, United States of America; 3 Department of Psychiatry and Behavioral Sciences, Emory University School of Medicine, Atlanta, Georgia, United States of America; University of New South Wales, AUSTRALIA

## Abstract

In 2008, the NIH launched an undiagnosed diseases program to investigate difficult to diagnose, and typically, multi-system diseases. The objective of this study was to evaluate the presence of psychiatric symptoms or psychiatric diagnoses in a cohort of patients seeking care at the Emory Special Diagnostic Service clinic. We hypothesized that psychiatric symptoms would be prevalent and associated with trauma exposure, and a decreased quality of life and functioning. This is a cross-sectional, retrospective analysis of 247 patients seen between February 7, 2014 and May 31, 2017. The sources for data included the Emory Health History Questionnaire (HHQ) that had the work and social adjustment and quality of life enjoyment and satisfaction questionnaire–short form (QLSQ) embedded in it; medical records, and the comprehensive standardized special diagnostic clinic forms. Primary outcomes were presence of any psychiatric symptom, based on report of the symptom on the HHQ or medical record, or presence of a confirmed preexisting psychiatric disorder. Seventy-two percent of patients had at least one psychiatric symptom while 24.3% of patients had a pre-existing psychiatric diagnosis. Patients with any psychiatric symptom had significantly diminished Q-LES-Q scores (45.27 ± 18.63) versus patients with no psychiatric symptoms (62.01 ± 21.57, t = 5.60, df = 225, p<0.0001) and they had significantly greater functional disability. Patients with a psychiatric disorder also had significantly diminished Q-LES-Q scores (45.16 ± 17.28) versus those without a psychiatric diagnosis (51.85 ± 21.54, t = 2.11, df = 225, p = 0.036) but did not have significantly increased functional impairment. Both patients with psychiatric symptoms and ones with psychiatric disorders had an increased prevalence of trauma. Psychiatric symptoms are prevalent in patients evaluated for undiagnosed disorders. The presence of any psychiatric symptom, with or without a formal psychiatric diagnosis, significantly decreases quality of life and functioning. This suggests that assessment for psychiatric symptoms should be part of the evaluation of individuals with undiagnosed disorders and may have important diagnostic and treatment implications.

## Introduction

More than a decade ago in 2008, the NIH launched an undiagnosed disease program to study difficult to diagnose, persistent multi-system syndromes of unknown etiology.[1] This program and other similar endeavors have been successful in identifying diagnoses in about 35% of patients, most of those diagnoses being either rare genetic disorders or newly identified genetic syndromes based on whole genome sequencing. [1–6] The Seavey Comprehensive Internal Medicine Clinic at Emory University School of Medicine recognized the need for such a clinic and developed the Emory Special Diagnostic Services (ESDS). Inspired by the NIH initiative, the ESDS was partially modeled after the NIH Undiagnosed Diseases Program.[7]

Psychiatric symptoms and psychiatric comorbidity are prevalent among patients with primary medical disorders with over 29% of medical patients a comorbid psychiatric disorder.[8, 9] The prevalence rates of comorbid depression alone range from 12–23% for outpatient cardiology patients, 12–18% for outpatients with diabetes, and from 16–36% of outpatients with HIV.[10–14] The presence of psychiatric disorders complicates the clinical course of the medical disorders and increases the cost of treatment.[10, 15] Furthermore, some patients with difficult to diagnose medical and neurological disorders may be suffering from unrecognized somatic symptom disorders or functional neurological disorders.[16–19] The presence of either psychiatric comorbidities or somatic symptom disorders is associated with decreased quality of life and functioning.[20–24] At this time there are no publications evaluating the presence nor impact of psychiatric symptoms nor co-morbid psychiatric disorders in patients evaluated in an Undiagnosed Diseases Clinic or in the NIH Undiagnosed Diseases Program. We hypothesized that psychiatric symptoms would be prevalent in this cohort of patients with difficult to diagnose symptoms. We further postulated that individuals with psychiatric symptoms or psychiatric disorders would be more disabled (in terms of physical or mental limitations), have a lower quality of life, and have greater functional impairment (in work, relationships, household functioning, etc.) than patients without psychiatric symptoms or disorders. We also postulated that subjects with psychiatric symptoms would be more likely to have a lifetime history of trauma.[19, 25]

## Methods

### Intake process and screening procedures

This study was approved by the Emory University Institutional Review Board (Approval number IRB00097006). This study was a retrospective chart review of de-identified data. Patients were referred to the ESDS by Emory healthcare or community physicians, healthcare administrators, family members, and self-referrals. The ESDS nurse navigator called referred patients to determine whether the reasons for seeking evaluation at ESDS aligned with the mission of the clinic: to evaluate patients whose symptoms were undiagnosed despite a reasonable medical work-up. The ESDS process was explained to patients, including required paperwork, records, and clinic fees, as well as a timeline for evaluation. Patients then completed an initial telephone interview and those passing the screening interview were requested to send their medical records for review. An in-depth review of the medical records was performed by the clinic director (CP). Inclusion and exclusion criteria followed the conventions used within the NIH Undiagnosed Diseases Network. Inclusion criteria included: presence of an undiagnosed symptom(s) or illness that had been extensively evaluated, reasonable expectation that evaluation by the ESDS could benefit the patient, and that there was still some diagnostic avenue to explore. Although there were no absolute exclusion criteria, patients presenting primarily for psychiatric complaints, as well as patients who had been referred for psychiatric evaluation but refused care, were not invited for an evaluation. Patients with primary diagnoses of chronic pain, fibromyalgia, and chronic fatigue that had been extensively evaluated were excluded as were patients whose histories suggested they would benefit from an evaluation by specific medical specialty.

Prior to evaluation, patients and/or their family members, completed a Health History Questionnaire (HHQ) that included the Work and Social Adjustment Scale (WSAS) and Quality of Life Enjoyment and Satisfaction Questionnaire-short form (Q-LES-Q).[26–29] The HHQ also included questions about demographic information, past medical and surgical history, a review of organ systems, screening questions about seven general mental health problems (anxiety, depression, mania, suicidal thoughts, delusions, hallucinations, and alcohol abuse), past emotional and mental health history, and trauma history and five questions that assess the impact of the current difficult to diagnose condition on work and family wellbeing (see S1 Form). The HHQ was designed to collect a broad range of background information for patient evaluation and diagnosis, rather than as a research instrument; as such, HHQ content was guided by clinical rather than theoretical considerations.

The physician reviewed all available medical records and the HHQ prior to an initial 1–2 hour evaluation. When needed, further testing was obtained and if necessary, a second appointment was arranged. Final diagnoses were assigned by the evaluating physician employing ICD-10 codes, following the completion of the ESDS evaluation (S1 Table).

### Data extraction for this study

From a detailed review of each patient’s Electronic Medical Record (EMR) and the HHQ that was completed by patients and/or their family members prior to evaluation at the clinic, we compiled and analyzed the following types of information for this report: presence of psychiatric symptoms/problems/complaints, formal psychiatric diagnoses received prior to ESDS evaluation, demographic characteristics, lifetime experience of trauma/abuse, ICD-10 diagnoses resulting from clinic consults, patients’ subjective rating of disability and functional impairment that they attribute to the current illness, and quality of life measured by level of subjective satisfaction with major areas of life prior to clinic evaluation.

Most data from the EMR came from the ESDS standardized template clinical note. A detailed EMR review was completed on each patient to extract pertinent information not found in the HHQ or ESDS evaluation note if the patient had been previously seen at Emory. For patients referred to Emory for evaluation external records were reviewed. The data were entered into a REDCAP database. The presence of psychiatric symptoms or psychiatric diagnosis was based on the HHQ and the review of all existing records. Outcomes for disability, functional impairment, and quality of life were extracted from the WSAS and Q-LES-Q sections of the HHQ along with 5 yes/no items about whether the current illness had caused the patient to quit working, cut back on working, feel it necessary to apply for disability benefits, receive disability benefits, or affected the patient’s professional or family well-being. The final ICD-10 diagnostic formulation was made by the clinic director (CP) and colleagues based on medical record information, the HHQ, the in-person evaluation, laboratory testing, and expert consultations.

### Definition of psychiatric symptoms/characteristics

The analyses were based on a broad definition and a narrow definition of psychiatric symptoms. The broad definition of psychiatric symptoms was defined as the presence of any psychiatric symptom or disorder from data collected from the HHQ and review of medical records. The narrow definition of a psychiatric disorder was the presence of a formal ICD-10 diagnosis of a psychiatric disorder in the patients’ medical records and/or HHQ.

### Data analysis

Statistical analysis consisted largely of computing frequency counts and comparing groups with chi-square or t-tests. The Wilcoxon Rank Sum Test was used to compare the number of ICD-10 diagnoses because of the non-normal distribution of this variable. The Holm correction, which is substantially less likely than the Bonferroni correction to inflate the number of false negatives, was used for determining statistical significance based on multiple comparisons.[30] Because of their high correlation with each other and with the summary score, component items of Q-LES-Q and WSAS scores were not counted as dependent variables for making the Holm adjustment; nor were variables that are a subset of another variable. The data tables include a footnote indicating statistical significance or non-significance for variables with uncorrected p-values <0.05 to which the Holm correction was applied.

We conducted two types of further analysis to examine how psychiatric symptoms are related to these patients’ quality of life and level of functioning. First, we used simple regression analysis to test group differences on Q-LES-Q and WSAS scores after covarying for possible confounding factors of age, gender, and marital status (married or not). Second, we performed stepwise regression analysis predicting Q-LES-Q and WSAS scores from other intake variables including each of the 2 definitions of psychiatric conditions, allowing forward stepping and backward stepping of a candidate variable based on meeting a p = 0.15 criterion for addition to or removal from the model.

## Results

### ESDS sample

Between September 6, 2014 and April 5, 2017, 917 patients contacted the clinic to inquire about evaluation. Sources of referral to the ESDS were: physicians (30.1%), patient self-referral (40.1%), web pages (4.9%), administrative (3.3%), or “other” (21.6%). Three hundred and forty-one patients met criteria for an evaluation. Reasons for exclusion from ESDS included: a chronic pain diagnosis (18.3%), previous extensive evaluations were unrevealing (23.7%), the patient failed to send medical records (17.2%), the patient refused evaluation (2.6%), the patient declined to pay the clinic one-time fee (1.6%), a presumptive diagnosis of somatoform disorder (10.6%), the patient was referred directly to a medical specialty clinic (11.3%), or “other” (14.6%). Of the 341 patients invited for the assessment, 269 of these patients were evaluated. Reasons for not attending the appointment included illness (6.9%), patient refusal (70.8%), inability to pay the fee (11.1%), resolution of symptoms (4.2%), or patients sought care elsewhere (6.9%).

Two hundred and forty-seven patients completed the HHQ, were evaluated, and were included in the analysis (Table 1). Approximately 47% of the sample were male, 53% female, and the mean age of the sample was 53.1 ± 18.2 years (range 17–86). Two thirds of patients were married. The overall sample was highly educated, with most patients having attended college (34.4%) or graduate school (22.4%).

**Table 1:** Demographic and clinical characteristics of the emory special diagnostic services (ESDS) sample (N = 247). Overall Sample and By Broad and Narrow Definitions of Psychiatric Complaints at Intake^a^.

**Demographics**		**Total** **(N = 247)**	**Broad: Any Psych. Sx or Dx**			**Narrow: Any Psych. Dx**		
			Yes (N = 178)	No (N = 69)	Significance	Yes (N = 60)	No (N = 187)	Significance
Age	Mean (sd)	53.1 (18.2)	52.0 (17.9)	56.0 (19.7)	t = 1.55, df = 245, p = 0.123	49.0 (18.3)	54.5 (18.0)	t = 2.02, df = 97.9^b^, p = 0.046^g^
	Median (Range)	56.0 (17–86)						
Gender	Male N (%)	116 (47.0)	76 (42.7)	40 (58.0)	Chi Sq = 4.66 df = 1, p = 0.031^g^	24 (40.0)	92 (49.2)	Chi Sq = 1.54, df = 1, p = 0.214
	Female N (%)	131 (53.0)	102 (57.3)	29 (42.0)		36 (60.0)	95 (50.8)	
Currently Married^c^	Yes N (%)	165 (67.1)	115 (65.0)	50 (72.5)	Chi Sq = 1.26, df = 1, p = 0.261	42 (70.0)	123 (66.1)	Chi Sq = 0.31, df = 1, p = 0.579
	No N (%)	81 (32.9)	62 (35.0)	19 (27.5)		18 (30.0)	63 (33.9)	
Education	High School or less N (%)	57 (23.1)	40 (22.7)	17 (24.6)	Chi Sq = 2.45, df = 3, p = 0.484	10 (16.7)	47 (25.4)	Chi Sq = 6.809, df = 3, p = 0.078
	Some College N (%)	47 (19.0)	35 (19.9)	12 (17.4)		8 (13.3)	39 (21.1)	
	Completed College N (%)	85 (34.4)	57 (32.4)	28 (40.6)		22 (36.7)	63 (34.0)	
	Graduate School N (%)	56 (22.4)	44 (25.0)	12 (17.4)		20 (33.3)	36 (19.5)	
	Tech/Trade N (%)	2 (0.8)	0 (0.0)	0 (0.0)		0 (0.0)	0 (0.0)	
**ClinicalCharacteristics**								
Any ICD-10 Diagnosis from Clinic Consults	Yes N (%)	173 (70.0)	132 (74.2)	41 (59.4)	Chi Sq = 5.15, df = 1, p = 0.023^g^	40 (66.7)	134 (71.7)	Chi Sq = 0.54, df = 1, p = 0.461
	No N (%)	74 (30.0)	46 (25.8)	28 (40.6)		20 (33.3)	53 (28.3)	
Any ICD-10 Psychiatric (F- Code) Dx from Clinic Consults	Yes N (%)	41 (23.7)	39 (29.1)	2 (4.9)	Chi Sq = 10.27, df = 1, p = 0.001	17 (41.5)	24 (17.9)	Chi Sq = 9.71, df = 1, p = 0.002
	No N (%)	132 (76.3)	95 (70.9)	39 (95.1)		24 (58.5)	110 (82.1)	
Any ICD-10 Non-Psych^d^ Dx from Clinic Consults	Yes N (%)	155 (89.6)	116 (87.9)	39 (95.1)	Chi Sq = 1.76, df = 1, p = 0.185	33 (82.5)	122 (91.7)	Chi Sq = 2.81, df = 1, p = 0.094
	No N (%)	18 (10.4)	16 (12.1)	2 (4.9)		7 (17.5)	11 (8.3)	
Total # of ICD-10 Diagnoses	1 N (%)	92 (53.2)	65 (49.2)	27 (65.8)	z = 3.23^d^, p = 0.001^f^	17 (42.5)	75 (56.4)	z = 3.07^e^, p = 0.002^f^
	2 N (%)	57 (33.0)	46 (34.8)	11 (26.8)		16 (40.0)	41 (30.8)	
	3 N (%)	13 (7.5)	11 (8.3)	2 (4.9)		4 (10.0)	9 (6.8)	
	4 N (%)	8 (4.6)	7 (5.3)	1 (2.4)		1 (2.5)	7 (5.3)	
	5 or 6 N (%)	3 (1.7)	3 (2.2)	0 (0.0)		2 (5.0)	1 (0.8)	
Lifetime Experience of Trauma/Abuse (No Info, N = 8)	Yes N (%)	36 (15.1)	32 (18.6)	4 (6.0)	Chi Sq = 6.02, df = 1, p = 0.008^g^	15 (25.9)	21 (11.6)	Chi Sq = 6.98, df = 1, p = 0.008^g^
	No N (%)	203 (84.9)	140 (81.4)	63 (94.0)		43 (74.1)	160 (88.4)	

^a^Based on all information sources at intake, regarding current or past psychiatric symptoms or diagnoses.

^b^After Satterthwaite adjustment for unequal group variances.

^c^One subject missed current marital status.

^d^ICD-10 diagnosis other than an F-code.

^e^Wilcoxon Rank Sum Test.

^f^Significant after Holm correction for multiple comparisons.

^g^Non-significant after Holm correction for multiple comparisons.

Thirty-six out of 239 patients (15.1%) reported experiencing lifetime trauma (Table 1). Sixteen patients declined to specify the type of trauma. Twelve patients identified one type, 6 patients reported two types, and 2 patients reported 3 types of traumatic experiences. The types of trauma experienced were: physical (5), sexual (9), emotional (6), victimization (3), combat (2), and witnessed trauma (5).

Mean scores for individual WSAS items (Table 2) include: work (5.03), home management (4.17), social leisure activities (4.56), private leisure activities (4.26), and family and relationships (2.75). Overall, 28.4% of patients reported stopping work and 47% of patients reported reducing work hours because of their illness. 20.9% of patients had applied for disability, and 7.2% of patients were receiving disability at intake. Approximately 71% of patients reported that their illness affected their professional or family wellbeing (Table 3). The final Q-LES-Q-SF score was standardized around a community sample with and without psychiatric disorders as described in the methods section. The overall Z-score of -2.47 for the 247 patient sample is in the bottom 1% relative to the normative population in terms of life satisfaction on this instrument (Table 4).

**Table 2:** Work and social impairment^a^ for esds sample (N = 247). Overall Sample and By Broad and Narrow Definitions of Psychiatric Complaints at Intake.

Areas of Functioning Mean (sd)/N	Total (N = 247)	Broad: Any Psych. Sx or Dx			Narrow: Any Psych Dx		
		Yes (N = 178)	No (N = 69)	Significance t, df, p	Yes (N = 60)	No (N = 187)	Significance t, df, p
Work	5.03 (2.80)/188	4.45 (2.61)/138	3.88 (3.01)/50	3.49, 186, 0.0006	4.93 (2.83)/46	5.06 (2.80)/142	0.27, 186, 0.788
Home Management	4.17 (2.84)/228	4.59 (2.38)/164	3.08 (2.82)/64	4.09, 226, <0.0001	4.25 (2.63)/55	4.14 (2.60)/173	0.29, 226, 0.774
Social Leisure Activities	4.56 (2.68)/230	4.92 (2.46)/165	3.63 (3.00)/65	3.36, 228, 0.001	4.72 (2.58)/57	4.50 (2.72)/173	0.53, 228, 0.600
Private Leisure Activities	4.26 (2.49)/231	4.48 (2.34)/167	3.70 (2.79)/64	2.13, 229, 0.034	4.12 (2.49)/57	4.31 (2.50)/174	0.49, 229, 0.623
Family and Relationships	2.75 (2.30)/229	3.13 (2.30)/166	1.73 (1.89)/63	4.28, 227, <0.0001	3.39 (2.29)/56	2.54 (2.27)/173	2.45, 227, 0.015
Sum of Above WSAS Items	20.51 (10.63)/222	20.40 (9.62)/161	15.52 (11.58)/61	4.49, 220, <0.0001^b^	21.63 (10.08)/54	20.15 (10.80)/168	0.89, 220, 0.373

^*a*^Work and Social Adjustment Scale impairment, rating scale is 0–8 per item, where 0 = not at all, 2 = slightly, 4 = definitely, 6 = markedly, and 8 = very severely for “how much your problem impairs your ability to carry out the activity.” The WSAS score is the sum of the 0–8 rating on each of the 5 items; if a single item was not answered, the average of the other 4 items was added to the sum to estimate response for that item.

^b^Significant after Holm correction for multiple comparisons.

**Table 3:** The Illness Impact^a^ for ESDS Sample (N = 247). Overall Sample and By Broad and Narrow Definitions of Psychiatric Complaints at Intake^b^.

Types of Impact N (%)		Total (N = 247)	Broad: Any Sx or Dx			Narrow: Any Dx		
			Yes (N = 178)	No (N = 69)	Significance (Chi Sq, df, p)	Yes (N = 60)	No (N = 187)	Significance (Chi Sq, df, p)
Had to quit working?	Yes	68 (28.7)	60 (34.9)	8 (12.3)	11.75, 1,<0.001^b^	13 (23.2)	55 (30.4)	1.08, 1, 0.300
	No	169 (71.3)	112 (65.1)	57 (87.7)		43 (76.8)	126 (69.6)	
	N	237	172	65		56	181	
Had to reduce work hours?	Yes	111 (47.0)	91 (52.9)	20 (31.2)	8.78, 1, 0.003^b^	24 (48.2)	84 (46.7)	0.04, 1, 0.839
	No	125 (53.0)	81 (47.1)	44 (68.8)		29 (51.8)	96 (53.3)	
	N	236	172	64		56	180	
Have felt it necessary to apply for disability benefits?	Yes	49 (20.9)	42 (24.6)	7 (10.9)	5.24, 1, 0.022^c^	12 (218)	37 (20.6)	0.04, 1, 0.841
	No	186 (79.1)	129 (75.4)	57 (89.1)		43 (78.2)	143 (79.4)	
	N	235	171	64		55	180	
Receiving disability benefits?	Yes	17 (7.2)	12 (7.0)	5 (7.8)	0.05, 1, 0.825	2 (3.6)	15 (8.3)	1.45, 1, 0.229
	No	219 (92.8)	160 (93.0)	59 (92.2)		54 (96.4)	165 (91.7)	
	N	236	172	64		56	180	
Has it affected your professional or family wellbeing?	Yes	167 (70.8)	134 (80.0)	33 (50.8)	17.33, 1, < .0001^b^	40 (71.4)	127 (70.6)	0.16, 1, 0.900
	No	69 (29.2)	37 (21.6)	32 (49.2)		16 (28.6)	53 (29.4)	
	N	236	171	65		56	180	

^*a*^Subjects were given response options Yes/No/Other regarding the impact due to their illness. The table shows Ns and percents based only on “Yes” and “No” responses, omitting “Other” or blank responses (which were due mainly to the item not being applicable to the subject).

^b^Significant after Holm correction for multiple comparisons.

^c^Non-significant after Holm correction for multiple comparisons.

**Table 4:** Quality of life enjoyment and satisfaction^a^ for ESDS sample (N = 247). Overall Sample and By Broad and Narrow Definitions of Psychiatric Complaints at Intake.

Items (Satisfaction in Past Week)^a^ Mean (sd)/N	Total (N = 247)	Broad: Any Psych. Sx or Dx			Narrow: Any Psych. Dx		
		Yes (N = 178)	No (N = 69)	Significance t, df, p	Yes (N = 60)	No (N = 187)	Significance t, df, p
Physical Health	2.28 (1.05)/237	2.17 (0.96)/172	2.55 (1.24)/65	2.23, 94.4^b^, 0.028	2.17 (1.08)/58	2.31 (1.05)/179	0.88, 235, 0.378
Mood	2.97 (1.03)/237	2.76 (0.95)/171	3.50 (1.06)/66	5.21, 235, <0.0001	2.76 (0.96)/58	3.03 (1.05)/179	1.77, 235, 0.078
Work	2.69 (1.26)/192	2.43 (1.18)/136	3.34 (1.21)/56	4.82, 190, <0.0001	2.52 (1.17)/46	2.75 (1.29)/146	1.06, 190, 0.292
Household Activities	2.57 (1.18)/236	2.36 (1.08)/170	3.09 (1.30)/66	4.39, 234, <0.0001	2.41 (106)/58	2.62 (1.22)/178	1.14, 234, 0.255
Social Relationships	3.16 (1.19)/231	2.90 (1.17)/165	3.80 (1.01)/66	5.53, 229, <0.0001	2.75 (1.14)/55	3.28 (1.19)/176	2.97, 229, 0.003
Family Relationships	3.77 (1.05)/235	3.52 (1.09)/170	4.40 (0.70)/65	7.33, 175^b^, <0.0001	3.36 (1.04)/58	3.90 (1.03)/177	3.44, 233, 0.001
Leisure Time Activities	2.50 (1.21)/232	2.35 (1.15)/166	2.88 (1.28)/66	3.05, 230, 0.002	2.39 (1.22)/57	1.54 (1.21)/175	0.82, 230, 0.415
Ability to Function in Daily Life	2.61 (1.20)/235	2.46 (1.13)/170	3.02 (1.30)/65	3.23, 233, 0.001	2.40 (1.21)/57	2.68 (1.20)/178	1.51, 233, 0.132
Sexual Drive/Interest	2.39 (1.24)/207	2.18 (1.15)/150	2.92 (1.33)/57	4.01, 205, <0.0001	2.06 (1.04)/68	2.48 (1.29)/159	2.07, 205, 0.039
Economic Status	3.68 (1.09)/226	3.51 (1.14)/163	4.11 (0.84)/63	4.34, 151^b^, <0.0001	3.64 (1.11)/55	3.69 (1.09)/171	0.32, 224, 0.752
Living/Housing Situation	4.10 (0.93)/234	3.96 (0.98)/169	4.45 (0.69)/65	4.25, 164^b^, <0.0001	4.03 (0.86)/58	4.12 (0.95)/176	0.60, 232, 0.547
Ability to Get Around Without Falling	3.20 (1.31)/238	3.09 (1.25)/172	3.48 (1.43)/66	2.08, 236, 0.039	3.05 (1.29)/58	3.25 (1.32)/180	1.00, 236, 0.318
Vision	3.42 (1.19)/236	3.26 (1.21)/171	3.85 (1.03)/65	3.47, 234, 0.0006	3.31 (1.20)/58	3.46 (1.19)/178	0.80, 234, 0.423
Overall Sense of Wellbeing	2.81 (1.12)/233	2.61 (1.03)/168	3.32 (1.20)/65	4.55, 231, <0.0001	2.57 (1.03)/58	2.89 (1.14)/175	1.87, 231, 0.062
Medication (if applicable)	3.16 (1.11)/180	3.01 (1.11)/130	3.54 (1.01)/50	2.95, 178, 0.004	2.87 (1.16)/45	3.25 (1.08)/135	2.04, 178, 0.043
Overall Life Satisfaction	2.75 (1.14)/239	2.61 (1.07)/172	3.12 (1.23)/65	3.16, 237, 0.002	2.52 (1.10)/58	2.83 (1.14)/181	1.82, 237, 0.070
**Score** Based on Items 1–14 for patients with no more than 2 items missing^c^ Mean (sd)/N	50.20 (20.73)/227	45.76 (18.63)/165	62.01 (21.57)/62	5.60, 225, <0.0001^d^	45.16 (17.28)/56	51.85 (21.54)/171	2.11, 225, 0.036^e^
Standardized (z-score) around community sample Mean (sd)/N	-2.47 (1.82)/227	-2.86 (1.63)/165	-1.44 (1.89)/62	5.60, 225, <0.0001	-2.92 (1.52)/56	-2.33 (1.89)/171	2.11, 225, 0.036

^a^Item response scale is 1 = very poor, 2 = poor, 3 = fair, 4 = good, 5 = very good for the subject’s satisfaction in the past week.

^b^After Satterthwaite adjustment for unequal group variances.

^c^Score is the percent of maximum possible, out of 12 to 14 items that were answered.

^d^Significant after Holm correction for multiple comparisons.

^e^Non-significant after Holm correction for multiple comparisons.

Of one hundred and seventy-three (70%) patients who received at least one final ICD-10 diagnosis following ESDS evaluation, 41 (23.7%) received a final psychiatric diagnosis. For patients that received a final diagnosis following evaluation, the number of final ICD-10 code diagnoses ranged from one (53.2%) to six (1.7%) (Table 1). The frequency of different types of ICD-10 final diagnoses for the 173 patients is shown in Supplemental Table 1 (S1 Table). The four most frequently given diagnoses were ICD codes for nervous system disorders, mental/behavioral health, musculoskeletal/connective tissue disorders, and digestive disorders.

Of the 247 patients, 69 (27.9%) had no psychiatric complaints and 178 (72.1%) had one or more psychiatric symptoms based on their responses to the 7 HHQ psychiatric items and past psychiatric history data in the HHQ or medical records. Fifty-nine (23.9%) had 1 psychiatric complaint, 70 (28.3%) had 2 psychiatric complaints, and 49 (19.8%) had 3 to 6 psychiatric complaints (3: N = 23, 4: N = 12, 5: N = 11, 6: N = 3). The predominance of symptoms endorsed were anxiety (N = 131) or depression symptoms (N = 118) (S2 Table). In comparison, only 24.3% of patients had a history of a formal psychiatric diagnosis (Table 1).

### Impact of any psychiatric symptom prior to evaluation

Patients with one or more psychiatric symptoms were more likely to be female and to have a lifetime history of trauma but did not differ on other demographic variables from those patients who did not report any psychiatric symptoms (Table 1). They were significantly more impaired than those without any psychiatric symptom on all five items of the WSAS and their sum (Table 2).

A significantly higher percentage of patients with at least one psychiatric symptom, compared to those without the presence of a psychiatric symptom, reported quitting work or reducing work hours, and applying for disability benefits do to their illness, and indicating that their illness had affected their professional or family wellbeing (Table 3).

They had significantly diminished Q-LES-Q scores (45.27 ± 18.63) versus patients with no psychiatric issues (62.01 ± 21.57, t = 5.60, df = 225, p<0.0001) (Table 4). The two groups differed significantly on every item of the Q-LES-Q.

Patients who endorsed having at least one psychiatric symptom on the HHQ or in the medical record, were more likely than patients without psychiatric symptoms to receive any final ICD-10 diagnosis, a final psychiatric diagnosis, and had a greater number of final ICD-10 diagnoses in their final diagnostic formulation by the ESDS (Table 1).

### Impact of a pre-existing psychiatric diagnosis prior to evaluation

Patients with an ICD, DSMIV or DSM 5 psychiatric diagnosis were slightly younger than patients without a formal psychiatric diagnosis and were more likely to have reported a history of trauma (Table 1). There was no significant difference in gender between these two groups (Table 1). In contradistinction to patients with vs. without any psychiatric symptoms, patients with formal psychiatric diagnoses differed from those without a psychiatric diagnosis on only one item of the WSAS, namely family and relationships (Table 2). They were also not more likely to quit or reduce work hours, receive or consider applying for disability, or have their illness affect their professional or family wellbeing compared to patients without formal psychiatric diagnoses (Table 3). Patients with a formal psychiatric disorder did have diminished Q-LES-Q scores (45.16 ± 17.28) versus those without a psychiatric diagnosis (51.85 ± 21.54, t = 2.11, df = 225, p = 0.036) (Table 4) but this was not significant after the Holm correction for multiple comparisons. The pattern of difference between the 2 groups based on having a psychiatric diagnosis was much more circumscribed than for groups defined by psychiatric symptoms. Patients who had received a formal psychiatric diagnosis by intake were more likely to receive a final ICD-10 formal psychiatric diagnosis following evaluation and were more likely to have a greater number of final ICD-10 diagnoses compared to patients without a psychiatric diagnosis (Table 1).

## Predictors of quality of life and functioning

In simple regression analysis, the broad definition of psychiatric complaints was a highly significant predictor of the Q-LES-Q score and the sum of 5 WSAS items, after covarying for age, gender, and education (p<0.001). The narrow definition significantly predicted Q-LES-Q score (p = 0.41) but not the WSAS item sum (p = 0.438). In stepwise regression analysis, once the broad definition of psychiatric complaints entered the predictive model for Q-LES-Q score or the sum of 5 WSAS items at the first step (at p<0.001 for both), no other variable met the p = 0.15 criterion for being added to the model. After the narrow definition entered the predictive model for the Q-LES-Q score (p = 0.075), female gender also entered the model (p = 0.066). The only variable to enter the stepwise model predicting the sum of 5 WSAS items was female gender (p = 0.064). See S3 Table for a summary of beta coefficients, R^2^, and p-values for these 8 regression analyses. See S1 Dataset for the three codebooks and data tables with the data underlying the reported means, medians, variance measure, p values and regression analyses.

## Discussion

Employing the broad definition of psychiatric characteristics, 72% of the patients evaluated in the ESDS program had psychiatric symptoms or diagnoses. This crude screening tool (S1 Form) plus review of medical records suggest that psychiatric symptoms are prevalent in this population of patients, even when patients with suspected somatization disorders, fibromyalgia, and pain disorders are excluded. Individuals with the presence of any symptom were more likely to be female, tended to be younger, and were more likely to have been exposed to trauma. These patients were much more disabled and functionally impaired, and had significantly worse quality of life than patients who did not have any psychiatric symptoms; this was true even after covarying for the possible confounding effects of age, gender, and education level. This association between the presence of any psychiatric symptom and decreased quality of life and greater functional disability is consistent with findings in the psychiatric literature that the presence of sub-syndromic depressive symptoms is associated with diminished quality of life and greater functional impairment.[31–34] As expected, these patients were more likely to have a final diagnosis, more final diagnoses, and at least one final psychiatric diagnosis. There is some overlap between the demographic characteristics of this cohort and somatic symptom disorder in that there were more women and greater exposure to trauma in this group; but unlike most cohorts with somatic symptom disorder, this cohort is well educated, younger in age, and not from a low socioeconomic status.[35]

Approximately 24% of patients had a psychiatric diagnosis recorded either in their medical records or HHQ. These patients also had decreased total Q-LES-Q scores, when contrasted to the rest of the patient population, with decreased scores on social and family relationships, sex drive, and medication satisfaction items, but did not differ from the rest of the cohort on measures of the WSAS nor questions about employment, home management, applying for disability, or lost productivity. They were more likely to have a final psychiatric diagnosis and to have experienced a significant traumatic life event than subjects without a formal pre-existing psychiatric diagnosis. This type of pattern of change where the broader criteria for defining inclusion into a specific cohort of subjects is associated with greater difference on outcome measures of interest (Q-LES-Q scores and items, WSAS items, and questions about employment and disability) than a narrower criteria for defining inclusion into a specific cohort of interest, is most consistent with the postulate that the narrower definition is overly restrictive, leading to a Type II error. These findings suggest that more systematic assessment with validated screening instruments is warranted in the evaluation of difficult to diagnose disorders population.

Our findings that psychiatric characteristics and symptoms are common in this undiagnosed disorders cohort of patients should not be surprising. Many disorders impact both the periphery of the body and the brain.[36, 37] However, we frequently do not evaluate the impact of these disorders on the brain. Furthermore, the stress of having sustained chronic unexplained symptoms should be expected to increase the presence not only of psychiatric symptoms but also of anxiety disorders and depression.[38–41] Despite the screening that was performed of potential candidates for the ESDS, a number of these patients had either somatic symptom disorders or possibly illness anxiety disorders. In fact, the patients referred for specialist care in neurology included the 6 who received an ICD-10 diagnosis of functional neurological disorders. However, it is important to recognize that somatic symptom disorders, illness anxiety disorders, and functional neurological disorders truly are biological disorders of circuitry in the brain and thus warrant careful assessment. In fact, including a more comprehensive battery of psychiatric measures and studies in the Undiagnosed Disease Network might facilitate our understanding of the genetics and circuit biology of fundamental processes like interoception, as well as more classical syndromes like somatic symptom disorder and functional neurological disorders. An additional important reason for expanding the psychiatric assessment of these patients is preliminary evidence suggesting that somatic symptom disorder, functional neurological disorders, and illness anxiety disorder may benefit from psychotherapy as well as physical and occupational therapy interventions.[42]

There are a number of limitations to this preliminary analysis of the prevalence and impact of psychiatric symptoms in a difficult to diagnose disease cohort of patients. First, the ascertainment of psychiatric symptoms and diagnoses in our database was relatively unsophisticated and requires refinement with the addition of standardized screening measures. We believe this may explain the low endorsement of lifetime history of trauma. Second, this is a retrospective analysis of a uniquely screened population of patients. The inclusion and exclusion criteria employed by the ESDS are similar to but distinct from other undiagnosed disease programs. Many of these programs are focused on genetic approaches and also see patients under age eighteen. However, our process has more similarities than differences to these existing programs. Another limitation of our data is that we suspect that the prevalence of final psychiatric diagnoses may be underreported since a validated psychiatric interview was not part of the standard evaluation of patients in the ESDS program. Despite these limitations, we believe that these data make a significant contribution to the field and suggest that more extensive and careful assessment of psychiatric symptoms and diagnoses should be part of undiagnosed disease program assessments. Furthermore, brain imaging and other biological measures may be useful in helping to characterize these patients and might, over time, lead to the identification of novel syndromes.

In conclusion, As Gahl and Tifft stated in their JAMA commentary, “The brain represents the next frontier for medicine.”[43] Our findings suggest that the presence of psychiatric symptoms or diagnoses are relatively common in our cohort of difficult to diagnose patients. The presence of psychiatric symptoms in this patient cohort is associated with more work and social disability and diminished quality of life when contrasted against patients who do not endorse any psychiatric symptoms. Thus, we believe that a more systematic assessment of psychiatric signs and symptoms as part of the evaluation of patients seen in an undiagnosed disease programs is warranted.

## Supporting information

S1 Form Health history questionnaire (HHQ). (DOC)

S1 Table ICD-10 final diagnosis categories for n = 247 emory special diagnostic service patients. (DOCX)

S2 Table Psychiatric symptoms/diagnoses at intake for n = 247 emory special diagnostic service patients. (DOCX)

S3 Table Details (beta coefficients, R^2^, and p-values) from regression analyses. (DOCX)

S1 Dataset Codebooks and data tables for all data underlying the reported means, medians, variance measure, p values and regression analyses. (DOCX)
