# Supplementary material for: The prevalence and impact of psychiatric symptoms in an undiagnosed diseases clinical program
Source: PLoS One. 2019 Jun 6;14(6):e0216937. doi: 10.1371/journal.pone.0216937 (PMC6553712; doi:10.1371/journal.pone.0216937)

**
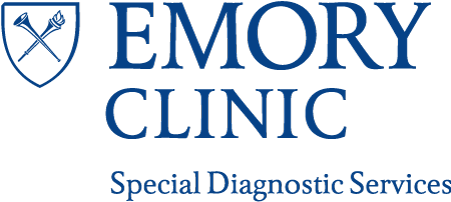
**

**Telephone: (404) 778-0990**

**Fax: (404) 778-0980**

**INITIAL DIAGNOSTIC SERVICES PROFILE**

MRN #:

*Per Diagnostic Staff*

**DEMOGRAPHICS**

Name: , ,

(Last) (First) (M.I.)

Age: E-mail address: ___________________________________________

Cell Number_________________________ Home/Business Number_______________________________

Date of Birth: / / M F Appointment Date: / /

Appointment with Dr. ______________ Occupation:

Pharmacy Information: Name of your preferred pharmacy: _________________________________________

Pharmacy Address: ___________________________________________________________________________

Pharmacy Phone: (_____) - ________________ Pharmacy Fax: (_____) - __________________

Personal Physician: (Name & Address)

_________

_______

______

Phone: ( ) - ______

Fax: ( ) - ______

Please sign below if you would like copies of your reports sent to your Primary Physician to update his/her files.

**Signature:** **Date:** ____/ / ____

*Revision Date 08/11/14*

**Please list your Diagnostic Concerns**

1. _________________ 2.)____________________ 3.)____________________

4.)__________________ 5.)____________________ 6.)____________________

| **Past Emotional Mental/Health History** | | | | |  |  |  |
| --- | --- | --- | --- | --- | --- | --- | --- |
|  |  |  |  |  |  |  |  |
| Please list any mental health problems which you have, or have had in the past. Indicate year of onset. | | | | | | | |

| **PROBLEM** | **ONSET** |
| --- | --- |
| (1) |  |
| (2) |  |
| (3) |  |
| (4) |  |

**Have you experienced the following events in your lifetime:**

**1. Have you ever seen something violent happen to someone (attacked or beaten) or seen someone killed?**

**YES____ NO____ Comments_____________________________________________________**

**2. Have you ever been attacked, mugged, robbed or been the victim of a serious crime?**

**YES____ NO____ Comments_____________________________________________________**

**3. Has anyone ever injured you with a weapon- gun, knife, stick, etc.?**

**YES____ NO____ Comments_____________________________________________________**

**4. Have you ever experienced a serious accident?**

**YES____ NO____ Comments_____________________________________________________**

**5. Have you ever lived in a combat or war zone or a political uprising?**

**YES____ NO____ Comments_____________________________________________________**

**6. Have you ever been physically abused or witnessed physical abuse?**

**YES____ NO____ Comments_____________________________________________________**

**7. Have you ever been sexually abused or witnessed sexual abuse?**

**YES____ NO____ Comments_____________________________________________________**

**8. Have you ever experienced any other traumatic event? Please explain.**

**YES____ NO____ Comments_____________________________________________________**

**LIFESTYLE FACTORS**

1. **Cigarette Use**

a. Current smoker ________ Past smoker ________ Year quit ________ Never smoked ____________

b. Average number of packs per day: c. Total number of years smoked: _____________

2. **Other Tobacco Use**

a. None ________ Current user ________ Past user ________ Year quit___________________

b. Pipe ________ Cigar ________ Smokeless ________ Number of years used: ________________

c. Electronic Cigarette _________

3. **Alcohol Use**

a. Average number of drinks per WEEK:

(One "drink" equals: 1 1/2 oz. liquor, 4 oz. wine, 12 oz. beer)

0-2:_______ 3-5: _______ 6-10: _______ 11-15: _______ 16-20: _______ 21-25: _______ 26+:_________

b. Drank in past but quit________ (Year quit________) Never drank___________

4. **Sleep:** Average number of hours per NIGHT: _______

5. **International travel in last two years:** Date(s) ____________________

Location(s)____________________________________________________________

**SOCIAL HISTORY**

**1. Marital Status:**

a. Currently married? ⁯Yes ⁯ No Years married: Spouse's Name: _

b. Number times married: _______ Divorced: ___________ Number of children: _______

1. **Education:**

College Graduate: ⁯Yes ⁯ No Year ______

Graduate Degree: Year ______

If no, highest grade achieved: _____________

1. **City and State of birth ____________________________**

**4.Previous Lyme Test Yes No If yes, number of tests________ If you have had previous Lyme test(s) number of positive Lyme tests________**

1. **Have you had to quit working because of this illness? YES___ NO____ Other____**
2. **Have you had to cut back the number of hours you**

**work due to this illness? YES___ NO____ Other____**

1. **Have felt it necessary to apply for disability benefits**

**due to this illness? YES___ NO____ Other____**

1. **Are you receiving disability benefits because of this**

**illness? YES____ NO____ Other____**

1. **Has this illness affected your professional or family**

**well being? YES____ NO____ Other____**

**Quality of Life Enjoyment and Satisfaction Questionnaire – Short Form**

**(Q-LES-Q-SF)**

Taking everything into consideration, during the past week how satisfied have you been with your………

|  | Very Poor | Poor | Fair | Good | Very Good |
| --- | --- | --- | --- | --- | --- |
| ….physical health? | 1 | 2 | 3 | 4 | 5 |
| ….mood? | 1 | 2 | 3 | 4 | 5 |
| ….work? | 1 | 2 | 3 | 4 | 5 |
| …household activities? | 1 | 2 | 3 | 4 | 5 |
| ….social relationships? | 1 | 2 | 3 | 4 | 5 |
| ….family relationships? | 1 | 2 | 3 | 4 | 5 |
| ….leisure time activities? | 1 | 2 | 3 | 4 | 5 |
| …ability to function in daily life? | 1 | 2 | 3 | 4 | 5 |
| …sexual drive, interest and/or performance? (If very poor, poor or fair underline the factor why) | 1 | 2 | 3 | 4 | 5 |
| …economic status? | 1 | 2 | 3 | 4 | 5 |
| …living/housing situation? (if very poor, poor or fair underline the factor why) | 1 | 2 | 3 | 4 | 5 |
| …ability to get around physically without feeling dizzy or unsteady or falling? (if very poor, poor or fair underline the factor why) | 1 | 2 | 3 | 4 | 5 |
| …your vision in terms of ability to do work or hobbies? (if very poor, poor or fair underline the factor why) | 1 | 2 | 3 | 4 | 5 |
| …overall sense of well being? | 1 | 2 | 3 | 4 | 5 |
| …medication? (If not taking any, check here____ and leave item blank) | 1 | 2 | 3 | 4 | 5 |
| …How would you rate your overall life satisfaction and contentment during the past week? | 1 | 2 | 3 | 4 | 5 |

**Work and Social Adjustment Scale (W&SAS)**

People's problems sometimes affect their ability to do certain day-to-day tasks in their lives. To rate your problems look at each section and determine on the scale provided how much your problem impairs your ability to carry out the activity.

*1.)* ***Work*** **–** if you are retired or choose not to have a job for reasons unrelated to your problem, please tick here

**0 1 2 3 4 5 6 7 8**

*not at all slightly definitely markedly very severely*

*I cannot work*

*2.)*  ***Home Management* –** cleaning, tidying, shopping, cooking, looking after home/children, paying bills etc

**0 1 2 3 4 5 6 7 8**

*not at all slightly definitely markedly very severely*

*3.)* ***Social Leisure Activities*** **–** with other people, e.g. parties, pubs, outings, entertaining etc

**0 1 2 3 4 5 6 7 8**

*not at all slightly definitely markedly very severely*

*4.)* ***Private Leisure Activities* –** done alone, e.g. reading, gardening, sewing, hobbies, walking etc

**0 1 2 3 4 5 6 7 8**

*not at all slightly definitely markedly very severely*

*5.)* ***Family & Relationships* –**form and maintain close relationships with others including the people that I live with

**0 1 2 3 4 5 6 7 8**

*not at all slightly definitely markedly very severely*


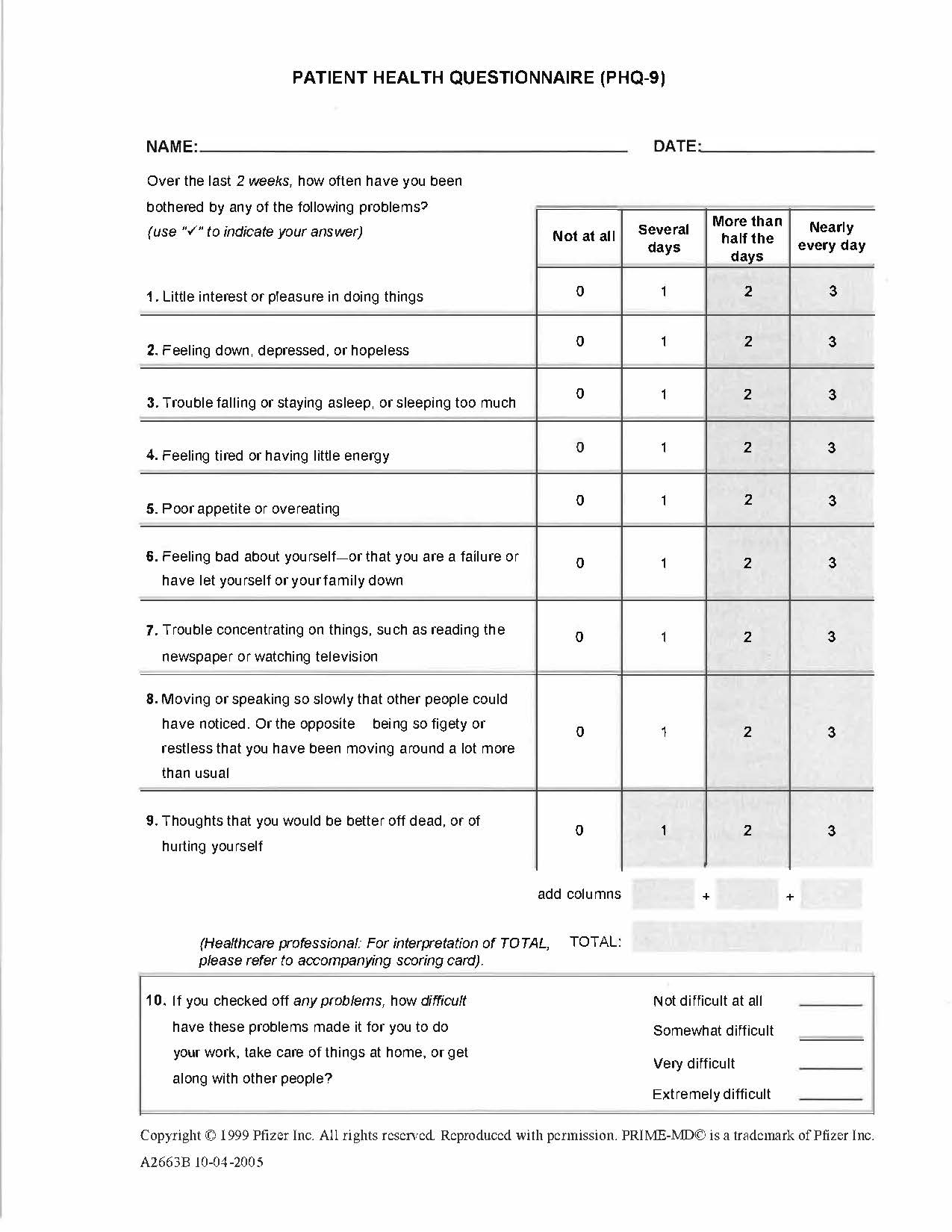

Supplement: S1 Form — (DOC) [file pone.0216937.s001.doc]
