# Supplementary material for: The prevalence and impact of psychiatric symptoms in an undiagnosed diseases clinical program
Source: PLoS One. 2019 Jun 6;14(6):e0216937. doi: 10.1371/journal.pone.0216937 (PMC6553712; doi:10.1371/journal.pone.0216937)
Supplement: S1 Table — (DOCX) [file pone.0216937.s002.docx]

S1 Table. ICD-10 Final Diagnosis^a^ Categories for N=247 Emory Special Diagnostic Service (ESDS) Patients.

| **ICD-10 Category** | ICD-10 Codes | Number of Subjects^b^  (% of 247) | Number of  Subjects with Multiple Diagnoses^c^ |
| --- | --- | --- | --- |
| Infectious/Parasitic | A00-B99 | 10 (4.05) | 0 |
| Neoplasms | C00-D49 | 13 (5.26) | 0 |
| Blood/Immune | D50-D89 | 5 (2.02) | 0 |
| Endocrine/Nutritional/Metabolic | E00-E89 | 12 (4.86) | 1 |
| Mental/Behavioral | F01-F99 | 41 (16.60)^d^ | 11 |
| Nervous System | G00-G99 | 45 (18.22) | 6 |
| Eye/Adnexa | H00-H59 | 2 (0.81) | 0 |
| Circulatory | I00-I99 | 11 (4.45) | 2 |
| Respiratory | J00-J99 | 14 (5.67) | 0 |
| Digestive | K00-K95 | 22 (8.91) | 2 |
| Skin/Subcutaneous | L00-L99 | 8 (3.24) | 0 |
| MS/Connective Tissue | M00-M99 | 26 (10.53) | 2 |
| Genitourinary | N00-N99 | 4 (1.62) | 1 |
| Congenital Malformation | Q00-Q99 | 5 (2.02) | 0 |
| Signs/Symptoms/Abnormalities | R00-R99 | 21 (8.50) | 0 |
| Injury/Poison/External Cause | S00-T88 | 10 (4.05) | 1 |
| Health Status Contact | Z00-Z99 | 2 (0.81) | 0 |
| Any ICD-10 Diagnosis | [All codes] | 173 (70.0) | 153^e^ |
| No ICD-10 Diagnosis | [No codes] | 74 (30.0) | 0 |

^a^ Final diagnoses were made by the clinic director and colleagues at the completion of the ESDS intake evaluation, based on medical record information, the intake Health History Questionnaire (HHQ), the in-person evaluation, laboratory testing, and expert consultation.

^b^ A subject is counted within an ICD-10 diagnostic category if his or her primary diagnosis or any of up to four secondary diagnoses was within the code range for that category. Thus, multiple diagnoses within a category for an individual subject were counted only once. The number of subjects is greater than 247 across categories because a subject could have diagnoses in multiple categories.

^c^ This column shows the total number of subjects with multiple diagnoses (primary or secondary) assigned within each ICD-10 category.

^d^ Six subjects received an F44 (functional neurologic disorder) diagnosis.

^e^ Of 247 subjects, 153 (61.94%) were given more than one diagnosis. For all but two of these subjects, there was a primary diagnosis and one or more secondary diagnoses; the remaining two subjects were each given two secondary, but no primary diagnosis.
