# Supplementary material for: The prevalence and impact of psychiatric symptoms in an undiagnosed diseases clinical program
Source: PLoS One. 2019 Jun 6;14(6):e0216937. doi: 10.1371/journal.pone.0216937 (PMC6553712; doi:10.1371/journal.pone.0216937)
Supplement: S2 Table — (DOCX) [file pone.0216937.s003.docx]

S2 Table. Psychiatric Symptoms/Diagnoses at Intake for n=247 Emory Special Diagnostic Services (ESDS) Patients.

| Broad Category | Code^a^ | Disorder | N (% of 247) |
| --- | --- | --- | --- |
| Mood Disorders | 01 | Depression/MDD | **118 (47.8)** |
|  | 05 | Dysthymia | 5 (2.0) |
|  | 55 | Mania | 1 (0.4) |
|  | 03 | Bipolar II Disorder | 3 (1.2) |
|  | 53 | Bipolar Disorder, Unspecified | 3 (1.2) |
|  | | | |
| Anxiety Disorders | 11 | Anxiety/GAD | **131 (53.0)** |
|  | 14 | Panic Disorder/Panic Attacks | 15 (6.1) |
|  | 13 | Social Anxiety Disorder | 1 (0.4) |
|  | 15 | Specific Phobias | 1 (0.4) |
|  | 16 | Obsessive-Compulsive Disorder | 10 (4.0) |
|  | 17 | PTSD | 4 (1.6) |
|  | 19 | Adjustment Disorder | 1 (0.4) |
|  | 20 | Somatic Symptom Disorder | 2 (0.8) |
|  | | | |
| Psychotic Disorders | 07 | Psychosis/Psychotic Episode | 2 (0.8) |
|  | 10 | Schizoaffective Disorder | 1 (0.4) |
|  | 57 | Delusional | 1 (0.4) |
|  | 58 | Hallucinations | 9 (3.6) |
|  | | | |
| Substance Use Disorders | 39 | Alcohol Abuse | 13 (5.3) |
|  | 40 | Illicit Drug Abuse | 5 (2.0) |
|  | | | |
| Sleep Disorders | 25 | Insomnia | 24 (9.7) |
|  | 26 | Narcolepsy | 2 (0.8) |
|  | 28 | REM Sleep Behavior Disorder | 1 (0.4) |
|  | 29 | Nightmare Disorder | 1 (0.4) |
|  | 62 | Sleep Disorder, Ill Defined | 2 (0.8) |
|  | | | |
| Eating Disorders | 23 | Anorexia | 1 (0.4) |
|  | 24 | Bulimia | 2 (0.8) |
|  | 61 | Eating Disorder, Unspecified | 2 (0.8) |
|  | | | |
| Functional Neurologic Disorders (FND) | 22 | Functional Neurologic Disorder | 2 (0.8) |
|  | 60 | Dissociative Symptoms or Disorder | 1 (0.4) |
|  | | | |
| Disorders Originating in Childhood | 32 | Intellectual Disability | 2 (0.8) |
|  | 34 | Autism Spectrum Disorder | 2 (0.8) |
|  | 35 | ADHD/ADD | 13 (5.3) |
|  | 36 | Tic Disorder | 1 (0.4) |
|  | | | |
| Suicidality | 52 | Past Suicide Attempt | 1 (0.4) |
|  | 56 | Suicidal Ideation | 7 (2.8) |
|  | 59 | Thoughts of Self Harm | 1 (0.4) |
|  | | | |
| **Any of the above** |  |  | 178 (72.1) |

^a^ Code was assigned arbitrarily during data compilation, based on all information sources at intake; it does not correspond to any diagnostic system.
