# Supplementary material for: The prevalence and impact of psychiatric symptoms in an undiagnosed diseases clinical program
Source: PLoS One. 2019 Jun 6;14(6):e0216937. doi: 10.1371/journal.pone.0216937 (PMC6553712; doi:10.1371/journal.pone.0216937)
Supplement: S3 Table — (DOCX) [file pone.0216937.s004.docx]

S3 Table - Details of Regression Analyses

First series of regression analyses: Prediction of Q-LES-Q quality of life or WSAS functioning score from broad or narrow definition of psychiatric characteristics, after covarying for age, female gender (dummy 0/1 variable where female = 1), and education (dummy 0/1 variable where completing college or some graduate school = 1).

| Prediction of This  Variable | Broad^a^ or Narrow^b^ Definition of Psychiatric Characteristics | βCoefficient | R^2^ | p-value |
| --- | --- | --- | --- | --- |
| Q-LES-Q Score | Broad | -15.69 | 0.133 | <0.0001 |
| Q-LES-Q Score | Narrow | -6.64 | 0.040 | 0.041 |
| WSAS Sum of 5 Items | Broad | 6.52 | 0.096 | <0.0001 |
| WSAS Sum of 5 Items | Narrow | 1.32 | 0.026 | 0.438 |

^a^Any psychiatric symptoms or formal diagnosis.

^b^Any formal psychiatric diagnosis.

Second series of regression analyses: Stepwise prediction of Q-LES-Q quality of life or WSAS functioning score from broad or narrow definition of psychiatric characteristics plus 5 other intake characteristics -- namely, age, female gender (dummy 0/1 variable where female = 1), and education (dummy 0/1 variable where completing college or some graduate school = 1), marital status (dummy 0/1 variable where married or living together = 1), and lifetime experience of trauma or abuse (dummy 0/1 variable where “yes” = 1) -- with p=0.15 criterion for a variable to be entered into or deleted from the model (forward or backward stepping).

| Prediction of This Variable  and Steps of Entry into  Predictive Model | Broad^a^ or Narrow^b^ Definition of Psychiatric Characteristics  As Candidate  Predictor | βCoefficient | R^2^ | p-value |
| --- | --- | --- | --- | --- |
| Q-LES-Q Score |  |  |  |  |
| Step 1: Variable Entered: Broad Definition    Step 2: No Further Variable Meets Entry Criterion | Broad  (entered)  --- | -15.33  --- | 0.111  --- | <0.0001  --- |
| Step 1 Variable Entered: Narrow Definition^c^  Step 2: Variable Entered: Female Gender | Narrow  (entered)  --- | -5.02  -5.97 | 0.020  0.034 | 0.075  0.066 |
| WSAS Sum of 5 Items |  |  |  |  |
| Step 1 Variable Entered: Broad Definition  Step 2: No Further Variable Meets Entry Criterion | Broad  (entered)  --- | 6.36  --- | 0.073  --- | <0.0001  --- |
| Step 1 Variable Entered: Female Gender  Step 2: No Further Variable Meets Entry Criterion | Narrow  (not entered)  --- | 2.69  --- | 0.016  --- | 0.064  --- |

^a^Any psychiatric symptoms or formal diagnosis.

^b^Any formal psychiatric diagnosis.

^c^Narrow definition of psychiatric characteristics remained in the model after female gender was entered in Step 2.
