# Supplementary material for: The prevalence and impact of psychiatric symptoms in an undiagnosed diseases clinical program
Source: PLoS One. 2019 Jun 6;14(6):e0216937. doi: 10.1371/journal.pone.0216937 (PMC6553712; doi:10.1371/journal.pone.0216937)
Supplement: S1 Dataset — (DOCX) [file pone.0216937.s005.docx]

S1 Dataset. Three codebooks and data tables with the data underlying the reported means, medians, variance measure, p values and regression analyses

**Data File A**

**Codebook for Data File A**

The following data elements were extracted from components of a REDCap database. These had been derived from detailed review by first author (G.W.) of the Home Health Questionnaire (HHQ), Electronic Medical Record (EMR), and any external records from referring sources.

Variable Description and Analysis Notes

record_id number (38-1087) used to link (merge) data for each of n=247 patients presenting to the Emory Special Diagnostic Service clinic from Feb. 7, 2014 and May 31, 2017

gender 1=male, 2=female

age range: 17-86

psycode1 to psycode6 2-digit code (01 to 62, as shown in S3 Table of the PLOS ONE article) assigned to classify up to 6 types of psychiatric symptoms, disorders, problems, or complaints experienced prior to the initial evaluation

psydx1 to psydx6 1=yes, 2=no, 3=unknown assigned to indicate whether the patient had ever received a formal diagnosis for the corresponding type of psychiatric symptom (1-6) above

trauma 1=yes, 2=no, 3=blank (not answered) for HHQ question asking whether the patient had ever experienced major psychological trauma such as physical, sexual, emotional abuse, victimization, combat, or witnessed trauma

type1 to type7 1=yes, 0=no for each of the following 7 types of trauma: physical, sexual, emotional, victimization, combat, witnessed trauma, or had trauma but left type unspecified

married 1=yes, 0=no to “Currently married?” question on HHQ

education 0-7 for highest level achieved: 0=blank, 1=less than grade 9, 2=some high school, 3=completed high school or equivalency test, 4=some college, 5=graduated from college, 6=some graduate school, 7=completed graduate school, 8=completed technical, trade, or professional non-academic program.

**Data File A**

| **File A** record_id | gender | age | psycode1 | psycode2 | psycode3 | psycode4 | psycode5 | psycode6 | psydx1 | psydx2 | psydx3 | psydx4 | psydx5 | psydx6 | trauma | type1 | type2 | type3 | type4 | type5 | type6 | type7 | married | education |
| --- | --- | --- | --- | --- | --- | --- | --- | --- | --- | --- | --- | --- | --- | --- | --- | --- | --- | --- | --- | --- | --- | --- | --- | --- |
| 38 | 2 | 56 | 1 | 11 |  |  |  |  | 1 | 1 |  |  |  |  | 2 | 0 | 0 | 0 | 0 | 0 | 0 | 0 | 1 | 7 |
| 39 | 2 | 52 | 1 | 11 |  |  |  |  | 1 | 1 |  |  |  |  | 2 | 0 | 0 | 0 | 0 | 0 | 0 | 0 | 1 | 5 |
| 43 | 2 | 61 |  |  |  |  |  |  |  |  |  |  |  |  | 3 | 0 | 0 | 0 | 0 | 0 | 0 | 0 | 0 | 5 |
| 46 | 2 | 37 | 11 | 14 | 1 |  |  |  | 3 | 3 | 3 |  |  |  | 1 | 0 | 1 | 0 | 0 | 0 | 1 | 0 | 1 | 4 |
| 47 | 1 | 81 | 11 | 3 |  |  |  |  | 2 | 1 |  |  |  |  | 2 | 0 | 0 | 0 | 0 | 0 | 0 | 0 | 1 | 6 |
| 49 | 2 | 43 | 1 | 56 |  |  |  |  | 1 | 3 |  |  |  |  | 1 | 0 | 0 | 0 | 0 | 0 | 0 | 1 | 0 | 7 |
| 54 | 1 | 68 | 1 | 11 |  |  |  |  | 3 | 3 |  |  |  |  | 2 | 0 | 0 | 0 | 0 | 0 | 0 | 0 | 1 | 5 |
| 55 | 2 | 27 | 1 |  |  |  |  |  | 1 |  |  |  |  |  | 2 | 0 | 0 | 0 | 0 | 0 | 0 | 0 | 1 | 5 |
| 58 | 2 | 44 |  |  |  |  |  |  |  |  |  |  |  |  | 2 | 0 | 0 | 0 | 0 | 0 | 0 | 0 | 1 | 7 |
| 65 | 2 | 32 | 1 | 11 | 16 |  |  |  | 1 | 1 | 1 |  |  |  | 1 | 1 | 1 | 0 | 0 | 0 | 0 | 0 | 1 | 5 |
| 69 | 2 | 42 | 11 | 1 |  |  |  |  | 2 | 2 |  |  |  |  | 2 | 0 | 0 | 0 | 0 | 0 | 0 | 0 | 1 | 7 |
| 72 | 2 | 39 | 11 |  |  |  |  |  | 1 |  |  |  |  |  | 1 | 0 | 0 | 0 | 0 | 0 | 0 | 1 | 1 | 5 |
| 73 | 2 | 49 | 1 |  |  |  |  |  | 2 |  |  |  |  |  | 2 | 0 | 0 | 0 | 0 | 0 | 0 | 0 | 1 | 8 |
| 74 | 1 | 72 |  |  |  |  |  |  |  |  |  |  |  |  | 3 | 0 | 0 | 0 | 0 | 0 | 0 | 0 | 1 | 4 |
| 76 | 2 | 34 | 1 | 11 |  |  |  |  |  |  |  |  |  |  | 2 | 0 | 0 | 0 | 0 | 0 | 0 | 0 | 0 | 3 |
| 78 | 2 | 71 | 1 |  |  |  |  |  | 3 |  |  |  |  |  |  | 0 | 0 | 0 | 0 | 0 | 0 | 0 | 1 | 3 |
| 79 | 2 | 41 |  |  |  |  |  |  |  |  |  |  |  |  | 2 | 0 | 0 | 0 | 0 | 0 | 0 | 0 | 0 | 5 |
| 80 | 1 | 78 | 1 |  |  |  |  | 59 | 1 |  |  |  |  | 2 | 3 | 0 | 0 | 0 | 0 | 0 | 0 | 0 | 1 | 7 |
| 84 | 1 | 69 | 39 |  |  |  |  |  |  |  |  |  |  |  | 2 | 0 | 0 | 0 | 0 | 0 | 0 | 0 | 1 | 3 |
| 85 | 1 | 23 | 11 | 1 |  |  |  |  | 2 | 2 |  |  |  |  | 2 | 0 | 0 | 0 | 0 | 0 | 0 | 0 | 0 | 4 |
| 87 | 1 | 19 | 53 | 1 | 10 | 11 | 26 |  | 3 | 3 | 3 | 3 | 3 |  | 2 | 0 | 0 | 0 | 0 | 0 | 0 | 0 | 0 | 4 |
| 91 | 1 | 84 | 11 | 1 |  |  |  |  | 3 |  |  |  |  |  | 2 | 0 | 0 | 0 | 0 | 0 | 0 | 0 | 1 | 5 |
| 92 | 1 | 65 | 53 | 11 | 39 | 40 |  |  | 1 | 3 | 1 | 1 |  |  | 2 | 0 | 0 | 0 | 0 | 0 | 0 | 0 | 1 | 7 |
| 93 | 2 | 19 |  |  |  |  |  |  |  |  |  |  |  |  | 2 | 0 | 0 | 0 | 0 | 0 | 0 | 0 | 0 | 4 |
| 94 | 2 | 69 | 11 | 1 |  |  |  |  | 3 | 3 |  |  |  |  | 2 | 0 | 0 | 0 | 0 | 0 | 0 | 0 | 0 | 7 |
| 96 | 2 | 40 |  |  |  |  |  |  |  |  |  |  |  |  | 2 | 0 | 0 | 0 | 0 | 0 | 0 | 0 | 0 | 5 |
| 99 | 1 | 75 |  |  |  |  |  |  |  |  |  |  |  |  | 2 | 0 | 0 | 0 | 0 | 0 | 0 | 0 | 1 | 5 |
| 105 | 1 | 63 | 16 |  |  |  |  |  | 1 |  |  |  |  |  | 2 | 0 | 0 | 0 | 0 | 0 | 0 | 0 | 1 | 5 |
| 107 | 2 | 47 | 25 | 1 | 11 |  |  |  | 3 |  | 3 |  |  |  | 1 | 0 | 0 | 0 | 0 | 0 | 1 | 0 | 1 | 5 |
| 110 | 1 | 81 |  |  |  |  |  |  |  |  |  |  |  |  | 2 | 0 | 0 | 0 | 0 | 0 | 0 | 0 | 1 | 3 |
| 111 | 1 | 53 | 1 | 39 |  |  |  |  | 1 | 1 |  |  |  |  | 2 | 0 | 0 | 0 | 0 | 0 | 0 | 0 | 1 | 7 |
| 113 | 2 | 63 | 11 | 1 |  |  |  |  | 2 | 2 |  |  |  |  | 2 | 0 | 0 | 0 | 0 | 0 | 0 | 0 | 1 | 5 |
| 115 | 2 | 30 | 1 | 11 |  |  |  |  | 1 | 3 |  |  |  |  | 2 | 0 | 0 | 0 | 0 | 0 | 0 | 0 | 1 | 5 |
| 116 | 2 | 78 | 11 | 1 |  |  |  |  | 3 | 3 |  |  |  |  | 2 | 0 | 0 | 0 | 0 | 0 | 0 | 0 | 0 | 4 |
| 120 | 1 | 85 | 1 |  |  |  |  |  | 3 |  |  |  |  |  | 2 | 0 | 0 | 0 | 0 | 0 | 0 | 0 | 0 | 3 |
| 121 | 2 | 20 |  |  |  |  |  |  |  |  |  |  |  |  | 2 | 0 | 0 | 0 | 0 | 0 | 0 | 0 | 0 | 4 |
| 122 | 1 | 52 | 1 |  |  |  |  |  | 3 |  |  |  |  |  | 2 | 0 | 0 | 0 | 0 | 0 | 0 | 0 | 1 | 7 |
| 124 | 1 | 47 |  |  |  |  |  |  |  |  |  |  |  |  | 2 | 0 | 0 | 0 | 0 | 0 | 0 | 0 | 1 | 5 |
| 125 | 1 | 56 | 1 | 11 | 39 |  |  |  | 3 | 3 | 1 |  |  |  | 2 | 0 | 0 | 0 | 0 | 0 | 0 | 0 | 1 | 3 |
| 126 | 2 | 68 | 1 | 11 |  |  |  |  | 2 | 2 |  |  |  |  | 2 | 0 | 0 | 0 | 0 | 0 | 0 | 0 | 1 | 4 |
| 127 | 1 | 74 | 1 |  |  |  |  |  | 3 |  |  |  |  |  | 2 | 0 | 0 | 0 | 0 | 0 | 0 | 0 | 1 | 3 |
| 128 | 2 | 47 | 11 | 1 |  |  |  |  | 3 | 3 |  |  |  |  | 2 | 0 | 0 | 0 | 0 | 0 | 0 | 0 | 1 | 3 |
| 129 | 1 | 43 |  |  |  |  |  |  |  |  |  |  |  |  | 2 | 0 | 0 | 0 | 0 | 0 | 0 | 0 | 1 | 7 |
| 130 | 1 | 43 | 11 | 1 |  |  |  |  |  |  |  |  |  |  | 2 | 0 | 0 | 0 | 0 | 0 | 0 | 0 | 1 | 7 |
| 131 | 2 | 30 | 11 | 1 | 16 | 25 | 14 |  | 1 | 1 | 1 | 1 | 3 |  | 1 | 0 | 0 | 0 | 0 | 0 | 0 | 0 | 1 | 5 |
| 132 | 1 | 82 | 11 | 1 |  |  |  |  | 1 | 3 |  |  |  |  | 1 | 0 | 0 | 0 | 0 | 0 | 0 | 0 | 1 | 7 |
| 133 | 1 | 40 |  |  |  |  |  |  |  |  |  |  |  |  | 2 | 0 | 0 | 0 | 0 | 0 | 0 | 0 | 0 | 5 |
| 134 | 2 | 53 | 11 |  |  |  |  |  |  |  |  |  |  |  | 2 | 0 | 0 | 0 | 0 | 0 | 0 | 0 | 0 | 5 |
| 135 | 2 | 24 | 1 | 52 | 11 | 39 | 40 |  |  | 3 | 3 | 3 | 1 |  | 1 | 0 | 0 | 0 | 0 | 0 | 0 | 1 | 0 | 3 |
| 136 | 2 | 46 | 15 |  |  |  |  |  | 2 |  |  |  |  |  | 2 | 0 | 0 | 0 | 0 | 0 | 0 | 0 | 0 | 5 |
| 137 | 2 | 60 | 11 |  |  |  |  |  | 2 |  |  |  |  |  | 1 | 0 | 0 | 0 | 0 | 0 | 0 | 1 | 1 | 4 |
| 138 | 1 | 76 | 1 |  |  |  |  |  | 3 |  |  |  |  |  | 2 | 0 | 0 | 0 | 0 | 0 | 0 | 0 | 1 | 3 |
| 139 | 2 | 25 | 34 | 58 |  |  |  |  | 1 | 3 |  |  |  |  | 1 | 0 | 0 | 0 | 0 | 0 | 0 | 1 | 0 | 3 |
| 140 | 1 | 75 | 25 |  |  |  |  |  | 3 |  |  |  |  |  | 1 | 0 | 0 | 0 | 1 | 0 | 0 | 0 | 1 | 5 |
| 142 | 1 | 58 | 11 |  |  |  |  |  |  |  |  |  |  |  | 2 | 0 | 0 | 0 | 0 | 0 | 0 | 0 | 1 | 7 |
| 146 | 2 | 19 | 35 | 1 | 23 | 24 | 25 |  | 1 | 3 | 1 | 3 |  |  | 2 | 0 | 0 | 0 | 0 | 0 | 0 | 0 | 0 | 4 |
| 152 | 2 | 58 | 11 | 1 |  |  |  |  | 3 | 3 |  |  |  |  | 2 | 0 | 0 | 0 | 0 | 0 | 0 | 0 | 1 | 5 |
| 159 | 1 | 42 | 11 |  |  |  |  |  | 1 |  |  |  |  |  | 2 | 0 | 0 | 0 | 0 | 0 | 0 | 0 | 1 | 5 |
| 160 | 2 | 36 | 34 | 11 | 5 | 17 |  |  | 3 | 3 | 3 | 1 |  |  | 1 | 0 | 1 | 0 | 0 | 0 | 0 | 0 | 0 | 3 |
| 162 | 2 | 52 | 1 |  |  |  |  |  | 3 |  |  |  |  |  | 2 | 0 | 0 | 0 | 0 | 0 | 0 | 0 | 1 | 5 |
| 164 | 2 | 69 |  |  |  |  |  |  |  |  |  |  |  |  | 2 | 0 | 0 | 0 | 0 | 0 | 0 | 0 | 0 | 7 |
| 165 | 2 | 31 | 1 | 11 | 14 | 25 |  |  | 1 | 1 | 3 | 1 |  |  | 2 | 0 | 0 | 0 | 0 | 0 | 0 | 0 | 0 | 5 |
| 166 | 1 | 79 | 25 | 1 |  |  |  |  | 3 | 3 |  |  |  |  | 2 | 0 | 0 | 0 | 0 | 0 | 0 | 0 | 1 | 7 |
| 171 | 1 | 74 | 1 | 11 |  |  |  |  | 3 | 3 |  |  |  |  | 2 | 0 | 0 | 0 | 0 | 0 | 0 | 0 | 1 | 3 |
| 173 | 2 | 17 | 11 |  |  |  |  |  | 3 |  |  |  |  |  | 2 | 0 | 0 | 0 | 0 | 0 | 0 | 0 | 0 | 2 |
| 183 | 2 | 29 | 35 | 17 |  |  |  |  | 1 | 1 |  |  |  |  | 1 | 0 | 0 | 0 | 0 | 0 | 1 | 0 | 1 | 6 |
| 185 | 2 | 43 |  |  |  |  |  |  |  |  |  |  |  |  | 2 | 0 | 0 | 0 | 0 | 0 | 0 | 0 | 1 | 5 |
| 190 | 2 | 36 | 1 | 11 | 25 | 17 |  |  | 1 | 3 | 3 | 3 |  |  | 2 | 0 | 0 | 0 | 0 | 0 | 0 | 0 | 1 | 7 |
| 191 | 2 | 51 | 1 |  |  |  |  |  | 3 |  |  |  |  |  | 2 | 0 | 0 | 0 | 0 | 0 | 0 | 0 | 0 | 4 |
| 198 | 1 | 45 | 1 | 11 |  |  |  |  | 3 | 3 |  |  |  |  | 2 | 0 | 0 | 0 | 0 | 0 | 0 | 0 | 0 | 5 |
| 199 | 2 | 40 | 1 |  |  |  |  |  | 3 |  |  |  |  |  | 2 | 0 | 0 | 0 | 0 | 0 | 0 | 0 | 0 | 5 |
| 202 | 2 | 58 | 11 |  |  |  |  |  | 3 |  |  |  |  |  | 2 | 0 | 0 | 0 | 0 | 0 | 0 | 0 | 1 | 5 |
| 203 | 1 | 68 |  |  |  |  |  |  |  |  |  |  |  |  | 2 | 0 | 0 | 0 | 0 | 0 | 0 | 0 | 1 | 7 |
| 207 | 1 | 72 | 11 | 1 | 16 |  |  |  | 1 | 1 | 3 |  |  |  | 3 | 0 | 0 | 0 | 0 | 0 | 0 | 0 | 1 | 7 |
| 208 | 2 | 31 | 11 |  |  |  |  |  | 3 |  |  |  |  |  | 1 | 1 | 0 | 1 | 1 | 0 | 0 | 0 | 1 | 6 |
| 210 | 1 | 40 | 35 |  |  |  |  |  | 1 |  |  |  |  |  | 2 | 0 | 0 | 0 | 0 | 0 | 0 | 0 | 1 | 5 |
| 213 | 2 | 20 | 35 |  |  |  |  |  | 1 |  |  |  |  |  | 2 | 0 | 0 | 0 | 0 | 0 | 0 | 0 | 0 | 4 |
| 221 | 2 | 40 |  |  |  |  |  |  |  |  |  |  |  |  | 2 | 0 | 0 | 0 | 0 | 0 | 0 | 0 | 1 | 7 |
| 225 | 2 | 59 | 16 | 11 | 36 |  |  |  | 3 | 3 | 3 |  |  |  | 2 | 0 | 0 | 0 | 0 | 0 | 0 | 0 | 1 | 7 |
| 230 | 1 | 45 |  |  |  |  |  |  |  |  |  |  |  |  | 2 | 0 | 0 | 0 | 0 | 0 | 0 | 0 | 0 | 7 |
| 234 | 2 | 22 | 1 | 11 | 56 | 14 |  |  |  |  |  | 3 |  |  | 1 | 0 | 1 | 0 | 0 | 0 | 0 | 0 | 0 | 5 |
| 236 | 2 | 45 | 11 | 1 |  |  |  |  | 1 | 2 |  |  |  |  | 2 | 0 | 0 | 0 | 0 | 0 | 0 | 0 | 1 | 7 |
| 241 | 1 | 71 | 11 | 14 | 60 |  |  |  | 1 | 3 | 2 |  |  |  | 2 | 0 | 0 | 0 | 0 | 0 | 0 | 0 | 1 | 5 |
| 242 | 2 | 46 | 1 | 11 | 25 | 13 |  |  | 1 | 1 | 3 | 3 |  |  | 2 | 0 | 0 | 0 | 0 | 0 | 0 | 0 | 1 | 3 |
| 245 | 2 | 18 |  |  |  |  |  |  |  |  |  |  |  |  | 1 | 0 | 0 | 0 | 0 | 0 | 0 | 1 | 0 | 4 |
| 246 | 1 | 27 | 14 | 1 | 11 | 35 | 25 |  | 1 |  | 1 | 1 | 3 |  | 2 | 0 | 0 | 0 | 0 | 0 | 0 | 0 | 0 | 5 |
| 256 | 2 | 39 | 11 | 1 | 25 |  |  |  | 1 | 3 | 3 |  |  |  | 1 | 1 | 0 | 1 | 0 | 0 | 1 | 0 | 0 | 3 |
| 262 | 1 | 51 | 11 |  |  |  |  |  | 3 |  |  |  |  |  | 2 | 0 | 0 | 0 | 0 | 0 | 0 | 0 | 1 | 3 |
| 277 | 1 | 72 |  |  |  |  |  |  |  |  |  |  |  |  | 2 | 0 | 0 | 0 | 0 | 0 | 0 | 0 | 1 | 5 |
| 354 | 1 | 62 | 11 |  |  |  |  |  | 3 |  |  |  |  |  | 2 | 0 | 0 | 0 | 0 | 0 | 0 | 0 | 1 | 5 |
| 361 | 2 | 33 | 11 |  |  |  |  |  | 3 |  |  |  |  |  | 2 | 0 | 0 | 0 | 0 | 0 | 0 | 0 | 1 | 5 |
| 362 | 2 | 58 | 11 |  |  |  |  |  | 1 |  |  |  |  |  | 2 | 0 | 0 | 0 | 0 | 0 | 0 | 0 | 1 | 7 |
| 367 | 2 | 57 | 1 |  |  |  |  |  |  |  |  |  |  |  | 1 | 1 | 0 | 1 | 0 | 0 | 0 | 0 | 1 | 3 |
| 383 | 1 | 29 | 11 |  |  |  |  |  | 3 |  |  |  |  |  | 2 | 0 | 0 | 0 | 0 | 0 | 0 | 0 | 1 | 5 |
| 398 | 2 | 71 | 1 | 39 |  |  |  |  | 3 | 1 |  |  |  |  | 2 | 0 | 0 | 0 | 0 | 0 | 0 | 0 | 0 | 5 |
| 401 | 2 | 61 | 11 | 1 |  |  |  |  |  |  |  |  |  |  | 1 | 0 | 0 | 0 | 0 | 0 | 0 | 0 | 1 | 4 |
| 411 | 2 | 23 |  |  |  |  |  |  |  |  |  |  |  |  | 2 | 0 | 0 | 0 | 0 | 0 | 0 | 0 | 0 | 5 |
| 421 | 2 | 55 | 11 | 1 | 25 |  |  |  | 1 | 3 |  |  |  |  | 2 | 0 | 0 | 0 | 0 | 0 | 0 | 0 | 1 | 3 |
| 441 | 2 | 62 | 1 |  |  |  |  |  | 3 |  |  |  |  |  | 2 | 0 | 0 | 0 | 0 | 0 | 0 | 0 | 1 | 7 |
| 443 | 1 | 62 | 1 | 11 | 58 | 7 |  |  | 2 | 2 | 2 | 2 |  |  | 1 | 0 | 0 | 0 | 0 | 0 | 1 | 1 | 0 | 5 |
| 448 | 2 | 19 | 11 |  |  |  |  |  | 3 |  |  |  |  |  | 2 | 0 | 0 | 0 | 0 | 0 | 0 | 0 | 0 | 4 |
| 450 | 2 | 33 | 11 | 14 | 55 | 25 | 35 | 1 |  |  |  |  | 1 |  | 1 | 0 | 1 | 0 | 0 | 0 | 0 | 0 | 1 | 5 |
| 452 | 1 | 57 | 1 |  |  |  |  |  | 1 |  |  |  |  |  | 2 | 0 | 0 | 0 | 0 | 0 | 0 | 0 | 1 | 7 |
| 453 | 2 | 38 | 11 | 1 | 20 |  |  |  | 3 | 3 | 1 |  |  |  | 2 | 0 | 0 | 0 | 0 | 0 | 0 | 0 | 0 | 4 |
| 454 | 1 | 20 | 11 | 61 |  |  |  |  | 2 | 2 |  |  |  |  | 2 | 0 | 0 | 0 | 0 | 0 | 0 | 0 | 0 | 4 |
| 455 | 2 | 68 | 1 | 11 |  |  |  |  | 1 | 3 |  |  |  |  | 2 | 0 | 0 | 0 | 0 | 0 | 0 | 0 | 1 | 4 |
| 464 | 2 | 52 | 1 | 11 |  |  |  |  |  |  |  |  |  |  | 2 | 0 | 0 | 0 | 0 | 0 | 0 | 0 | 0 | 4 |
| 466 | 1 | 41 | 11 | 14 |  |  |  |  |  |  |  |  |  |  | 2 | 0 | 0 | 0 | 0 | 0 | 0 | 0 | 0 | 7 |
| 469 | 2 | 39 | 1 | 11 |  |  |  |  | 1 | 3 |  |  |  |  | 2 | 0 | 0 | 0 | 0 | 0 | 0 | 0 | 1 | 7 |
| 471 | 2 | 76 |  |  |  |  |  |  |  |  |  |  |  |  | 2 | 0 | 0 | 0 | 0 | 0 | 0 | 0 | 0 | 5 |
| 472 | 1 | 40 |  |  |  |  |  |  |  |  |  |  |  |  | 2 | 0 | 0 | 0 | 0 | 0 | 0 | 0 | 1 | 5 |
| 481 | 2 | 63 | 1 | 11 |  |  |  |  |  |  |  |  |  |  | 1 | 0 | 0 | 0 | 0 | 0 | 0 | 1 | 1 | 4 |
| 483 | 2 | 28 | 11 |  |  |  |  |  | 3 |  |  |  |  |  | 2 | 0 | 0 | 0 | 0 | 0 | 0 | 0 | 0 | 6 |
| 487 | 2 | 22 | 1 | 11 | 56 | 14 | 25 |  | 2 | 2 | 2 | 2 | 2 |  | 1 | 0 | 1 | 0 | 1 | 0 | 0 | 1 | 0 | 5 |
| 491 | 2 | 66 | 11 | 1 |  |  |  |  |  |  |  |  |  |  | 2 | 0 | 0 | 0 | 0 | 0 | 0 | 0 |  | 5 |
| 493 | 1 | 77 | 1 | 11 |  |  |  |  | 3 | 3 |  |  |  |  | 2 | 0 | 0 | 0 | 0 | 0 | 0 | 0 | 1 | 4 |
| 498 | 2 | 57 |  |  |  |  |  |  |  |  |  |  |  |  | 2 | 0 | 0 | 0 | 0 | 0 | 0 | 0 | 1 | 1 |
| 499 | 2 | 53 | 1 | 16 | 11 | 35 | 14 |  | 1 | 3 | 1 | 1 | 3 |  | 2 | 0 | 0 | 0 | 0 | 0 | 0 | 0 | 1 | 4 |
| 504 | 1 | 22 | 1 | 35 | 11 | 14 |  |  |  |  | 3 | 3 |  |  | 2 | 0 | 0 | 0 | 0 | 0 | 0 | 0 | 0 | 4 |
| 506 | 1 | 76 |  |  |  |  |  |  |  |  |  |  |  |  | 2 | 0 | 0 | 0 | 0 | 0 | 0 | 0 | 0 | 7 |
| 512 | 1 | 51 |  |  |  |  |  |  |  |  |  |  |  |  | 2 | 0 | 0 | 0 | 0 | 0 | 0 | 0 | 1 | 3 |
| 513 | 2 | 33 |  |  |  |  |  |  |  |  |  |  |  |  | 2 | 0 | 0 | 0 | 0 | 0 | 0 | 0 | 1 | 3 |
| 514 | 1 | 65 |  |  |  |  |  |  |  |  |  |  |  |  | 2 | 0 | 0 | 0 | 0 | 0 | 0 | 0 | 1 | 3 |
| 515 | 2 | 33 | 1 | 11 | 57 | 58 | 39 | 22 |  |  | 2 | 2 | 1 | 1 | 1 | 0 | 0 | 0 | 0 | 0 | 0 | 0 | 0 | 5 |
| 518 | 2 | 44 |  |  |  |  |  |  |  |  |  |  |  |  | 2 | 0 | 0 | 0 | 0 | 0 | 0 | 0 | 1 | 4 |
| 519 | 2 | 67 | 11 | 53 | 16 | 35 | 20 |  | 3 | 3 | 2 | 3 | 3 |  | 2 | 0 | 0 | 0 | 0 | 0 | 0 | 0 | 1 | 3 |
| 520 | 2 | 29 | 11 |  |  |  |  |  | 2 |  |  |  |  |  | 2 | 0 | 0 | 0 | 0 | 0 | 0 | 0 | 0 | 5 |
| 525 | 2 | 51 | 1 | 11 |  |  |  |  |  |  |  |  |  |  | 1 | 0 | 0 | 0 | 0 | 0 | 0 | 0 | 0 | 5 |
| 527 | 2 | 68 |  |  |  |  |  |  |  |  |  |  |  |  | 2 | 0 | 0 | 0 | 0 | 0 | 0 | 0 | 0 | 5 |
| 528 | 2 | 66 | 1 | 11 |  |  |  |  | 3 | 3 |  |  |  |  | 2 | 0 | 0 | 0 | 0 | 0 | 0 | 0 | 0 | 3 |
| 529 | 2 | 59 | 11 |  |  |  |  |  | 3 |  |  |  |  |  | 2 | 0 | 0 | 0 | 0 | 0 | 0 | 0 | 0 | 3 |
| 531 | 2 | 21 |  |  |  |  |  |  |  |  |  |  |  |  | 2 | 0 | 0 | 0 | 0 | 0 | 0 | 0 | 0 | 4 |
| 538 | 1 | 72 | 1 | 25 |  |  |  |  | 1 | 3 |  |  |  |  | 2 | 0 | 0 | 0 | 0 | 0 | 0 | 0 | 1 | 7 |
| 542 | 2 | 61 | 1 | 11 |  |  |  |  |  |  |  |  |  |  | 2 | 0 | 0 | 0 | 0 | 0 | 0 | 0 | 1 | 3 |
| 546 | 2 | 78 | 1 | 11 | 14 |  |  |  | 3 | 1 | 3 |  |  |  | 2 | 0 | 0 | 0 | 0 | 0 | 0 | 0 | 1 | 4 |
| 547 | 1 | 62 | 1 | 39 | 40 | 62 |  |  | 3 | 3 | 3 | 3 |  |  | 2 | 0 | 0 | 0 | 0 | 0 | 0 | 0 | 1 | 3 |
| 549 | 2 | 52 | 11 |  |  |  |  |  | 3 |  |  |  |  |  | 2 | 0 | 0 | 0 | 0 | 0 | 0 | 0 | 1 | 5 |
| 559 | 1 | 74 | 1 | 11 |  |  |  |  | 1 | 3 |  |  |  |  | 2 | 0 | 0 | 0 | 0 | 0 | 0 | 0 | 0 | 7 |
| 561 | 2 | 80 |  |  |  |  |  |  |  |  |  |  |  |  | 2 | 0 | 0 | 0 | 0 | 0 | 0 | 0 | 0 | 7 |
| 569 | 1 | 20 |  |  |  |  |  |  |  |  |  |  |  |  | 2 | 0 | 0 | 0 | 0 | 0 | 0 | 0 | 0 | 4 |
| 575 | 1 | 59 |  |  |  |  |  |  |  |  |  |  |  |  | 2 | 0 | 0 | 0 | 0 | 0 | 0 | 0 | 1 | 5 |
| 595 | 1 | 68 | 11 |  |  |  |  |  | 3 |  |  |  |  |  | 1 | 0 | 0 | 0 | 0 | 1 | 0 | 0 | 1 | 5 |
| 596 | 2 | 66 |  |  |  |  |  |  |  |  |  |  |  |  | 2 | 0 | 0 | 0 | 0 | 0 | 0 | 0 | 1 | 5 |
| 607 | 1 | 80 | 58 |  |  |  |  |  | 2 |  |  |  |  |  | 2 | 0 | 0 | 0 | 0 | 0 | 0 | 0 | 0 | 7 |
| 611 | 2 | 84 | 1 | 11 |  |  |  |  | 1 | 3 |  |  |  |  | 2 | 0 | 0 | 0 | 0 | 0 | 0 | 0 | 0 | 7 |
| 614 | 1 | 59 |  |  |  |  |  |  |  |  |  |  |  |  | 2 | 0 | 0 | 0 | 0 | 0 | 0 | 0 | 1 | 4 |
| 622 | 1 | 33 |  |  |  |  |  |  |  |  |  |  |  |  | 2 | 0 | 0 | 0 | 0 | 0 | 0 | 0 | 1 | 5 |
| 629 | 2 | 73 | 1 | 58 |  |  |  |  | 3 | 2 |  |  |  |  | 2 | 0 | 0 | 0 | 0 | 0 | 0 | 0 | 1 | 4 |
| 632 | 1 | 57 |  |  |  |  |  |  |  |  |  |  |  |  | 2 | 0 | 0 | 0 | 0 | 0 | 0 | 0 | 1 | 4 |
| 638 | 1 | 85 |  |  |  |  |  |  |  |  |  |  |  |  | 2 | 0 | 0 | 0 | 0 | 0 | 0 | 0 | 1 | 5 |
| 640 | 1 | 36 | 1 | 11 |  |  |  |  |  |  |  |  |  |  | 3 | 0 | 0 | 0 | 0 | 0 | 0 | 0 | 0 | 5 |
| 641 | 1 | 56 | 35 |  |  |  |  |  |  |  |  |  |  |  | 2 | 0 | 0 | 0 | 0 | 0 | 0 | 0 | 1 | 4 |
| 644 | 2 | 61 |  |  |  |  |  |  |  |  |  |  |  |  | 2 | 0 | 0 | 0 | 0 | 0 | 0 | 0 | 1 | 3 |
| 649 | 2 | 65 | 1 | 39 |  |  |  |  | 3 | 3 |  |  |  |  | 2 | 0 | 0 | 0 | 0 | 0 | 0 | 0 | 1 | 5 |
| 650 | 1 | 44 |  |  |  |  |  |  |  |  |  |  |  |  | 2 | 0 | 0 | 0 | 0 | 0 | 0 | 0 | 1 | 3 |
| 651 | 2 | 70 | 1 |  |  |  |  |  | 3 |  |  |  |  |  | 2 | 0 | 0 | 0 | 0 | 0 | 0 | 0 | 0 | 4 |
| 652 | 1 | 62 | 11 | 28 |  |  |  |  | 3 | 1 |  |  |  |  | 2 | 0 | 0 | 0 | 0 | 0 | 0 | 0 | 1 | 5 |
| 653 | 2 | 76 |  |  |  |  |  |  |  |  |  |  |  |  | 2 | 0 | 0 | 0 | 0 | 0 | 0 | 0 | 1 | 3 |
| 654 | 2 | 72 |  |  |  |  |  |  |  |  |  |  |  |  | 2 | 0 | 0 | 0 | 0 | 0 | 0 | 0 | 1 | 3 |
| 656 | 2 | 66 | 1 |  |  |  |  |  | 2 |  |  |  |  |  | 2 | 0 | 0 | 0 | 0 | 0 | 0 | 0 | 1 | 5 |
| 662 | 1 | 62 | 11 | 1 | 39 |  |  |  | 1 | 1 | 3 |  |  |  | 2 | 0 | 0 | 0 | 0 | 0 | 0 | 0 | 1 | 5 |
| 663 | 1 | 62 | 1 | 11 |  |  |  |  | 3 | 3 |  |  |  |  | 2 | 0 | 0 | 0 | 0 | 0 | 0 | 0 | 0 | 3 |
| 665 | 1 | 54 |  |  |  |  |  |  |  |  |  |  |  |  | 1 | 0 | 0 | 0 | 0 | 0 | 0 | 0 | 1 | 3 |
| 670 | 1 | 48 | 1 | 11 |  |  |  |  |  |  |  |  |  |  | 2 | 0 | 0 | 0 | 0 | 0 | 0 | 0 | 1 | 5 |
| 672 | 1 | 66 | 1 |  |  |  |  |  |  |  |  |  |  |  | 2 | 0 | 0 | 0 | 0 | 0 | 0 | 0 | 1 | 7 |
| 674 | 1 | 71 |  |  |  |  |  |  |  |  |  |  |  |  | 2 | 0 | 0 | 0 | 0 | 0 | 0 | 0 | 1 | 4 |
| 677 | 1 | 46 | 58 |  |  |  |  |  |  |  |  |  |  |  | 2 | 0 | 0 | 0 | 0 | 0 | 0 | 0 | 1 | 3 |
| 678 | 1 | 46 | 17 | 1 |  |  |  |  | 3 | 3 |  |  |  |  | 1 | 0 | 0 | 0 | 0 | 0 | 0 | 0 | 1 | 5 |
| 685 | 1 | 50 |  |  |  |  |  |  |  |  |  |  |  |  | 2 | 0 | 0 | 0 | 0 | 0 | 0 | 0 | 1 | 5 |
| 686 | 1 | 25 |  |  |  |  |  |  |  |  |  |  |  |  | 2 | 0 | 0 | 0 | 0 | 0 | 0 | 0 | 1 | 2 |
| 689 | 2 | 76 |  |  |  |  |  |  |  |  |  |  |  |  | 2 | 0 | 0 | 0 | 0 | 0 | 0 | 0 | 1 | 5 |
| 690 | 2 | 79 | 1 | 11 |  |  |  |  | 3 | 3 |  |  |  |  | 2 | 0 | 0 | 0 | 0 | 0 | 0 | 0 | 0 | 3 |
| 693 | 1 | 41 | 11 | 1 |  |  |  |  | 3 | 2 |  |  |  |  | 3 | 0 | 0 | 0 | 0 | 0 | 0 | 0 | 0 | 7 |
| 695 | 1 | 69 |  |  |  |  |  |  |  |  |  |  |  |  | 2 | 0 | 0 | 0 | 0 | 0 | 0 | 0 | 1 | 5 |
| 697 | 2 | 39 | 1 | 11 | 35 | 56 | 14 |  | 1 | 1 | 1 | 2 | 3 |  | 1 | 1 | 0 | 1 | 0 | 0 | 0 | 1 | 1 | 5 |
| 699 | 2 | 23 | 11 | 1 |  |  |  |  | 3 | 3 |  |  |  |  | 2 | 0 | 0 | 0 | 0 | 0 | 0 | 0 | 1 | 3 |
| 701 | 2 | 83 | 1 | 25 |  |  |  |  |  | 3 |  |  |  |  | 2 | 0 | 0 | 0 | 0 | 0 | 0 | 0 | 1 | 3 |
| 702 | 1 | 46 | 16 | 11 | 1 |  |  |  | 3 | 3 | 3 |  |  |  | 1 | 0 | 0 | 0 | 0 | 0 | 0 | 0 | 1 | 4 |
| 703 | 1 | 32 |  |  |  |  |  |  |  |  |  |  |  |  | 2 | 0 | 0 | 0 | 0 | 0 | 0 | 0 | 1 | 5 |
| 705 | 1 | 67 |  |  |  |  |  |  |  |  |  |  |  |  | 2 | 0 | 0 | 0 | 0 | 0 | 0 | 0 | 1 | 7 |
| 707 | 2 | 78 |  |  |  |  |  |  |  |  |  |  |  |  | 1 | 0 | 0 | 0 | 0 | 0 | 0 | 0 | 1 | 4 |
| 708 | 2 | 35 | 11 | 1 | 32 |  |  |  | 3 | 3 | 3 |  |  |  | 3 | 0 | 0 | 0 | 0 | 0 | 0 | 0 | 0 | 3 |
| 709 | 2 | 59 | 1 | 11 |  |  |  |  | 3 | 3 |  |  |  |  | 2 | 0 | 0 | 0 | 0 | 0 | 0 | 0 | 1 | 4 |
| 711 | 2 | 55 |  |  |  |  |  |  |  |  |  |  |  |  | 2 | 0 | 0 | 0 | 0 | 0 | 0 | 0 | 1 | 4 |
| 714 | 1 | 74 |  |  |  |  |  |  |  |  |  |  |  |  | 2 | 0 | 0 | 0 | 0 | 0 | 0 | 0 | 1 | 5 |
| 721 | 2 | 31 | 1 | 11 |  |  |  |  | 3 | 3 |  |  |  |  | 2 | 0 | 0 | 0 | 0 | 0 | 0 | 0 | 1 | 3 |
| 723 | 1 | 67 |  |  |  |  |  |  |  |  |  |  |  |  | 2 | 0 | 0 | 0 | 0 | 0 | 0 | 0 | 1 | 5 |
| 725 | 1 | 75 | 1 | 56 | 11 |  |  |  | 3 | 2 | 3 |  |  |  | 2 | 0 | 0 | 0 | 0 | 0 | 0 | 0 | 1 | 5 |
| 727 | 2 | 43 | 11 | 35 |  |  |  |  | 3 | 1 |  |  |  |  | 1 | 0 | 0 | 1 | 0 | 0 | 0 | 0 | 1 | 7 |
| 729 | 1 | 86 |  |  |  |  |  |  |  |  |  |  |  |  | 2 | 0 | 0 | 0 | 0 | 0 | 0 | 0 | 0 | 3 |
| 730 | 1 | 59 | 1 | 11 |  |  |  |  |  |  |  |  |  |  | 2 | 0 | 0 | 0 | 0 | 0 | 0 | 0 | 0 | 7 |
| 733 | 1 | 64 |  |  |  |  |  |  |  |  |  |  |  |  | 2 | 0 | 0 | 0 | 0 | 0 | 0 | 0 | 1 | 3 |
| 736 | 1 | 28 | 19 | 11 |  |  |  |  | 3 | 3 |  |  |  |  | 2 | 0 | 0 | 0 | 0 | 0 | 0 | 0 | 0 | 4 |
| 738 | 2 | 20 | 11 | 25 |  |  |  |  | 3 | 3 |  |  |  |  | 2 | 0 | 0 | 0 | 0 | 0 | 0 | 0 | 0 | 4 |
| 739 | 1 | 31 |  |  |  |  |  |  |  |  |  |  |  |  | 2 | 0 | 0 | 0 | 0 | 0 | 0 | 0 | 1 | 5 |
| 741 | 1 | 33 |  |  |  |  |  |  |  |  |  |  |  |  | 2 | 0 | 0 | 0 | 0 | 0 | 0 | 0 | 1 | 7 |
| 742 | 2 | 32 | 1 | 11 |  |  |  |  | 2 |  |  |  |  |  | 2 | 0 | 0 | 0 | 0 | 0 | 0 | 0 | 0 | 4 |
| 743 | 1 | 65 |  |  |  |  |  |  |  |  |  |  |  |  | 2 | 0 | 0 | 0 | 0 | 0 | 0 | 0 | 1 | 7 |
| 747 | 2 | 78 | 1 |  |  |  |  |  |  |  |  |  |  |  | 2 | 0 | 0 | 0 | 0 | 0 | 0 | 0 | 1 | 5 |
| 748 | 1 | 69 |  |  |  |  |  |  |  |  |  |  |  |  | 2 | 0 | 0 | 0 | 0 | 0 | 0 | 0 | 1 | 5 |
| 751 | 1 | 33 | 25 | 11 |  |  |  |  | 3 |  |  |  |  |  | 2 | 0 | 0 | 0 | 0 | 0 | 0 | 0 | 1 | 4 |
| 767 | 1 | 55 | 11 |  |  |  |  |  | 3 |  |  |  |  |  | 2 | 0 | 0 | 0 | 0 | 0 | 0 | 0 | 1 | 5 |
| 769 | 2 | 56 | 1 | 11 |  |  |  |  | 3 | 3 |  |  |  |  | 2 | 0 | 0 | 0 | 0 | 0 | 0 | 0 | 1 | 5 |
| 770 | 1 | 19 | 5 | 11 | 26 | 56 | 58 | 29 | 1 | 3 | 1 | 2 | 2 | 3 | 2 | 0 | 0 | 0 | 0 | 0 | 0 | 0 | 0 | 4 |
| 771 | 2 | 25 | 11 | 1 | 61 | 16 | 22 |  | 3 | 3 | 2 | 2 | 3 |  | 2 | 0 | 0 | 0 | 0 | 0 | 0 | 0 | 0 | 7 |
| 782 | 1 | 60 | 25 |  |  |  |  |  | 1 |  |  |  |  |  | 2 | 0 | 0 | 0 | 0 | 0 | 0 | 0 | 1 | 7 |
| 785 | 1 | 73 |  |  |  |  |  |  |  |  |  |  |  |  | 2 | 0 | 0 | 0 | 0 | 0 | 0 | 0 | 0 | 3 |
| 789 | 1 | 63 | 1 |  |  |  |  |  | 3 |  |  |  |  |  | 2 | 0 | 0 | 0 | 0 | 0 | 0 | 0 | 1 | 3 |
| 790 | 2 | 56 |  |  |  |  |  |  |  |  |  |  |  |  | 2 | 0 | 0 | 0 | 0 | 0 | 0 | 0 | 1 | 2 |
| 791 | 2 | 54 | 1 | 11 | 7 |  |  |  | 3 | 3 | 3 |  |  |  | 3 | 0 | 0 | 0 | 0 | 0 | 0 | 0 | 1 | 3 |
| 792 | 1 | 52 | 11 | 5 |  |  |  |  | 3 | 3 |  |  |  |  | 2 | 0 | 0 | 0 | 0 | 0 | 0 | 0 | 0 | 7 |
| 793 | 2 | 78 |  |  |  |  |  |  |  |  |  |  |  |  | 2 | 0 | 0 | 0 | 0 | 0 | 0 | 0 | 0 | 3 |
| 794 | 2 | 60 | 1 |  |  |  |  |  | 3 |  |  |  |  |  | 2 | 0 | 0 | 0 | 0 | 0 | 0 | 0 | 1 | 3 |
| 796 | 1 | 74 | 11 | 25 |  |  |  |  | 3 | 3 |  |  |  |  | 2 | 0 | 0 | 0 | 0 | 0 | 0 | 0 | 1 | 5 |
| 803 | 1 | 82 | 39 |  |  |  |  |  | 1 |  |  |  |  |  | 2 | 0 | 0 | 0 | 0 | 0 | 0 | 0 | 1 | 5 |
| 809 | 1 | 57 | 1 | 11 |  |  |  |  | 3 | 3 |  |  |  |  | 2 | 0 | 0 | 0 | 0 | 0 | 0 | 0 | 1 | 2 |
| 812 | 1 | 56 | 32 |  |  |  |  |  | 1 |  |  |  |  |  | 2 | 0 | 0 | 0 | 0 | 0 | 0 | 0 | 1 | 2 |
| 813 | 1 | 69 |  |  |  |  |  |  |  |  |  |  |  |  | 2 | 0 | 0 | 0 | 0 | 0 | 0 | 0 | 1 | 7 |
| 815 | 2 | 41 |  |  |  |  |  |  |  |  |  |  |  |  | 2 | 0 | 0 | 0 | 0 | 0 | 0 | 0 | 1 | 5 |
| 816 | 2 | 50 | 1 | 25 | 16 |  |  |  | 3 | 1 | 3 |  |  |  | 2 | 0 | 0 | 0 | 0 | 0 | 0 | 0 | 1 | 5 |
| 825 | 2 | 40 | 1 | 11 | 62 |  |  |  | 3 | 3 | 2 |  |  |  | 2 | 0 | 0 | 0 | 0 | 0 | 0 | 0 | 1 | 7 |
| 830 | 1 | 41 | 1 | 11 | 40 | 39 |  |  | 3 | 3 | 1 | 3 |  |  | 2 | 0 | 0 | 0 | 0 | 0 | 0 | 0 | 1 | 3 |
| 838 | 2 | 50 | 1 | 11 | 24 |  |  |  | 1 | 3 | 1 |  |  |  | 2 | 0 | 0 | 0 | 0 | 0 | 0 | 0 | 1 | 7 |
| 842 | 1 | 49 | 11 | 14 |  |  |  |  | 1 | 3 |  |  |  |  | 2 | 0 | 0 | 0 | 0 | 0 | 0 | 0 | 1 | 4 |
| 847 | 1 | 58 | 1 | 25 | 11 |  |  |  | 3 | 3 | 3 |  |  |  | 2 | 0 | 0 | 0 | 0 | 0 | 0 | 0 | 1 | 2 |
| 856 | 2 | 60 | 11 |  |  |  |  |  | 3 |  |  |  |  |  | 1 | 0 | 1 | 1 | 0 | 0 | 0 | 0 | 0 | 8 |
| 858 | 1 | 36 | 1 | 11 | 56 | 39 | 40 |  | 1 |  |  | 1 | 1 |  | 2 | 0 | 0 | 0 | 0 | 0 | 0 | 0 | 0 | 5 |
| 863 | 1 | 32 | 11 |  |  |  |  |  | 3 |  |  |  |  |  | 2 | 0 | 0 | 0 | 0 | 0 | 0 | 0 | 1 | 7 |
| 870 | 1 | 63 | 1 | 11 |  |  |  |  | 3 | 3 |  |  |  |  | 2 | 0 | 0 | 0 | 0 | 0 | 0 | 0 | 1 | 4 |
| 871 | 2 | 41 | 11 |  |  |  |  |  | 3 |  |  |  |  |  | 2 | 0 | 0 | 0 | 0 | 0 | 0 | 0 | 1 | 5 |
| 874 | 1 | 64 | 25 | 1 | 58 | 11 |  |  | 3 | 3 | 2 | 3 |  |  | 2 | 0 | 0 | 0 | 0 | 0 | 0 | 0 | 1 | 5 |
| 880 | 2 | 73 | 11 |  |  |  |  |  | 3 |  |  |  |  |  | 2 | 0 | 0 | 0 | 0 | 0 | 0 | 0 | 0 | 7 |
| 882 | 1 | 59 | 11 | 1 | 58 |  |  |  | 3 | 3 | 2 |  |  |  | 2 | 0 | 0 | 0 | 0 | 0 | 0 | 0 | 1 | 4 |
| 890 | 2 | 43 | 11 | 14 | 25 | 5 |  |  | 3 | 3 | 3 | 3 |  |  | 2 | 0 | 0 | 0 | 0 | 0 | 0 | 0 | 1 | 7 |
| 899 | 2 | 22 | 11 | 5 | 35 |  |  |  | 3 | 3 | 1 |  |  |  | 2 | 0 | 0 | 0 | 0 | 0 | 0 | 0 | 0 | 5 |
| 903 | 1 | 44 | 11 |  |  |  |  |  | 3 |  |  |  |  |  | 2 | 0 | 0 | 0 | 0 | 0 | 0 | 0 | 1 | 7 |
| 911 | 2 | 74 | 11 |  |  |  |  |  | 3 |  |  |  |  |  | 2 | 0 | 0 | 0 | 0 | 0 | 0 | 0 | 0 | 7 |
| 914 | 1 | 72 |  |  |  |  |  |  |  |  |  |  |  |  | 2 | 0 | 0 | 0 | 0 | 0 | 0 | 0 | 1 | 5 |
| 920 | 2 | 67 | 1 | 11 |  |  |  |  | 1 | 1 |  |  |  |  | 1 | 0 | 1 | 0 | 0 | 0 | 0 | 0 | 0 | 3 |
| 929 | 2 | 79 | 11 | 1 | 25 |  |  |  | 3 | 3 | 3 |  |  |  | 2 | 0 | 0 | 0 | 0 | 0 | 0 | 0 | 1 | 4 |
| 935 | 2 | 65 | 11 |  |  |  |  |  | 3 |  |  |  |  |  | 2 | 0 | 0 | 0 | 0 | 0 | 0 | 0 | 1 | 3 |
| 954 | 2 | 37 | 1 |  |  |  |  |  | 2 |  |  |  |  |  | 1 | 0 | 1 | 0 | 0 | 0 | 0 | 1 | 0 | 7 |
| 959 | 1 | 67 |  |  |  |  |  |  |  |  |  |  |  |  | 2 | 0 | 0 | 0 | 0 | 0 | 0 | 0 | 1 | 5 |
| 968 | 1 | 66 | 1 | 11 |  |  |  |  |  |  |  |  |  |  | 2 | 0 | 0 | 0 | 0 | 0 | 0 | 0 | 0 | 3 |
| 972 | 2 | 69 |  |  |  |  |  |  |  |  |  |  |  |  | 2 | 0 | 0 | 0 | 0 | 0 | 0 | 0 | 1 | 3 |
| 977 | 1 | 73 | 1 | 11 |  |  |  |  | 3 | 3 |  |  |  |  | 2 | 0 | 0 | 0 | 0 | 0 | 0 | 0 | 0 | 4 |
| 1087 | 1 | 69 |  |  |  |  |  |  |  |  |  |  |  |  | 1 | 0 | 0 | 0 | 0 | 1 | 0 | 0 | 1 | 5 |

**DATA FILE B**

**Codebook for Data File B**

The following data elements were extracted from components of a REDCap database section containing ICD-10 codes for diagnoses received as a result of ESDS evaluation.

Variable Description and Analysis Notes

record_id number (38-1087) used to link (merge) data for each of n=247 patients presenting to the Emory Special Diagnostic Service clinic from Feb. 7, 2014 and May 31, 2017

resolved 1=yes, 0=no indicating whether the patient’s chief complaint was resolved as a result of ESDS evaluation and treatment

num_F_codes the number of primary or secondary ICD-10 F-code (psychiatric) diagnoses assigned through initial or 1-year evaluation

num_notF_codes the number of primary or secondary non-psychiatric ICD-10 code (i.e., not F-code) diagnoses assigned through initial or 1-year evaluation

final_prim_icd10 ICD-10 code for primary diagnosis following initial evaluation

second1_icd10  ICD-10 code for a secondary diagnosis following initial evaluation

second2_icd10 ICD-10 code for a secondary diagnosis following initial evaluation

second3_icd10 ICD-10 code for a secondary diagnosis following initial evaluation

second4_icd10 ICD-10 code for a secondary diagnosis following initial evaluation

second5_icd10 ICD-10 code for a secondary diagnosis following initial evaluation

new_unif_icd10 ICD-10 code for a new unifying diagnosis at 1-year evaluation

add1_icd10  ICD-10 code for a diagnosis added at 1-year evaluation

add2_icd10 ICD-10 code for a diagnosis added at 1-year evaluation

add3_icd10 ICD-10 code for a diagnosis added at 1-year evaluation

**Data File B**

| **File B**  record_id | resolved | num_F_codes | num_notF_codes | final_prim_icd10 | second1_icd10 | second2_icd10 | second3_icd10 | second4_icd10 | second5_icd10 | new_unif_icd10 | add1_icd10 | add2_icd10 | add3_icd10 |
| --- | --- | --- | --- | --- | --- | --- | --- | --- | --- | --- | --- | --- | --- |
| 38 | 0 | 0 | 0 |  |  |  |  |  |  |  |  |  |  |
| 39 | 0 | 0 | 0 |  |  |  |  |  |  |  |  |  |  |
| 43 | 1 | 0 | 2 | E11.9 | R21 |  |  |  |  |  |  |  |  |
| 46 | 0 | 3 | 0 | F32.9 | F41.9 |  | F43.10 |  |  |  |  |  |  |
| 47 | 1 | 1 | 2 | R55 | R00.1 | F31.9 |  |  |  |  |  |  |  |
| 49 | 0 | 0 | 0 |  |  |  |  |  |  |  |  |  |  |
| 54 | 1 | 1 | 3 | J64 | Z56.5 | R41.9 | F32.9 |  |  |  |  |  |  |
| 55 | 1 | 0 | 1 | K81.9 |  |  |  |  |  |  |  |  |  |
| 58 | 1 | 0 | 3 | D83.9 | R19.7 | K57.90 |  |  |  |  |  |  |  |
| 65 | 1 | 3 | 3 | E61.1 | E87.6 | R31.9 |  |  |  |  |  |  |  |
| 69 | 1 | 0 | 0 |  |  |  |  |  |  |  |  |  |  |
| 72 | 0 | 0 | 2 | R06.4 | G47.9 |  |  |  |  |  |  |  |  |
| 73 | 1 | 0 | 1 | M84.353A |  |  |  |  |  |  |  |  |  |
| 74 | 1 | 0 | 1 | C64.9 |  |  |  |  |  |  |  |  |  |
| 76 | 1 | 0 | 1 | G43.909 |  |  |  |  |  |  |  |  |  |
| 78 | 0 | 0 | 2 | K86.8 | K25.9 |  |  |  |  |  |  |  |  |
| 79 | 1 | 0 | 0 |  |  |  |  |  |  |  |  |  |  |
| 80 | 0 | 0 | 0 |  |  |  |  |  |  |  |  |  |  |
| 84 | 1 | 1 | 4 | J18.9 | I31.9 | F10.20 | J84.9 | I48.91 |  |  |  |  |  |
| 85 | 1 | 0 | 2 | M10.00 | B35.9 |  |  |  |  |  |  |  |  |
| 87 | 1 | 1 | 1 | G90.91 | F31.9 |  |  |  |  |  |  |  |  |
| 91 | 1 | 0 | 1 | M31.6 |  |  |  |  |  |  |  |  |  |
| 92 | 0 | 0 | 0 |  |  |  |  |  |  |  |  |  |  |
| 93 | 1 | 0 | 1 | A49.1 |  |  |  |  |  |  |  |  |  |
| 94 | 1 | 2 | 2 | F32.9 | F45.9 | G47.30 | N39.3 |  |  |  |  |  |  |
| 96 | 1 | 1 | 0 | F44.4 |  |  |  |  |  |  |  |  |  |
| 99 | 0 | 0 | 0 |  |  |  |  |  |  |  |  |  |  |
| 105 | 0 | 0 | 1 | C64.9 |  |  |  |  |  |  |  |  |  |
| 107 | 0 | 0 | 0 |  |  |  |  |  |  |  |  |  |  |
| 110 | 1 | 0 | 2 | T88.7XX4 | T88.7XX4 |  |  |  |  |  |  |  |  |
| 111 | 1 | 0 | 2 | R04.2 | T88.7XX4 |  |  |  |  |  |  |  |  |
| 113 | 0 | 0 | 0 |  |  |  |  |  |  |  |  |  |  |
| 115 | 0 | 0 | 0 |  |  |  |  |  |  |  |  |  |  |
| 116 | 0 | 0 | 2 | G23.9 | G90.9 |  |  |  |  |  |  |  |  |
| 120 | 1 | 0 | 1 | B86 |  |  |  |  |  |  |  |  |  |
| 121 | 0 | 0 | 0 |  |  |  |  |  |  |  |  |  |  |
| 122 | 1 | 0 | 2 | C37 | K63.9 |  |  |  |  |  |  |  |  |
| 124 | 1 | 0 | 0 |  |  |  |  |  |  |  |  |  |  |
| 125 | 1 | 0 | 1 | R53.83 |  |  |  |  |  |  |  |  |  |
| 126 | 1 | 0 | 1 | I26.99 |  |  |  |  |  |  |  |  |  |
| 127 | 0 | 0 | 0 |  |  |  |  |  |  |  |  |  |  |
| 128 | 1 | 0 | 2 | G47.33 | J43.9 |  |  |  |  |  |  |  |  |
| 129 | 0 | 0 | 4 | M79.2 | G62.9 | T88.7XXA | J98.6 |  |  |  |  |  |  |
| 130 | 1 | 0 | 2 | E75.6 | D36.9 |  |  |  |  |  |  |  |  |
| 131 | 1 | 0 | 2 | K31.89 | E27.40 |  |  |  |  |  |  |  |  |
| 132 | 0 | 0 | 0 |  |  |  |  |  |  |  |  |  |  |
| 133 | 1 | 0 | 1 | M08.20 |  |  |  |  |  |  |  |  |  |
| 134 | 1 | 0 | 2 | K21.9 | E21.3 |  |  |  |  |  |  |  |  |
| 135 | 0 | 1 | 1 | S74.02XS | F11.20 |  |  |  |  |  |  |  |  |
| 136 | 0 | 0 | 0 |  |  |  |  |  |  |  |  |  |  |
| 137 | 0 | 0 | 2 | M54.9 | M47.819 |  |  |  |  |  |  |  |  |
| 138 | 1 | 0 | 1 | M31.6 |  |  |  |  |  |  |  |  |  |
| 139 | 0 | 0 | 2 | G43.A0 | G43.909 |  |  |  |  |  |  |  |  |
| 140 |  | 0 | 0 |  |  |  |  |  |  |  |  |  |  |
| 142 | 1 | 0 | 4 | J45.909 | M85.80 | G47.9 | E03.9 |  |  |  |  |  |  |
| 146 | 0 | 0 | 0 |  |  |  |  |  |  |  |  |  |  |
| 152 | 1 | 0 | 1 | L12.1 |  |  |  |  |  |  |  |  |  |
| 159 | 1 | 0 | 2 | Q67.6 | K58.9 |  |  |  |  |  |  |  |  |
| 160 | 0 | 0 | 0 |  |  |  |  |  |  |  |  |  |  |
| 162 | 0 | 0 | 1 | M77.9 |  |  |  |  |  |  |  |  |  |
| 164 | 0 | 0 | 0 |  |  |  |  |  |  |  |  |  |  |
| 165 | 1 | 0 | 3 | K58.9 | G43.909 | J34.2 |  |  |  |  |  |  |  |
| 166 | 0 | 0 | 0 |  |  |  |  |  |  |  |  |  |  |
| 171 | 0 | 1 | 2 | G23.9 | G62.9 | F32.9 |  |  |  |  |  |  |  |
| 173 | 1 | 0 | 1 | G43.909 |  |  |  |  |  |  |  |  |  |
| 183 | 0 | 0 | 1 | Q87.89 |  |  |  |  |  |  |  |  |  |
| 185 |  | 0 | 0 |  |  |  |  |  |  |  |  |  |  |
| 190 | 0 | 1 | 0 | F44.9 |  |  |  |  |  |  |  |  |  |
| 191 | 1 | 0 | 2 | L95.0 | M79.7 |  |  |  |  |  |  |  |  |
| 198 | 0 | 0 | 0 |  |  |  |  |  |  |  |  |  |  |
| 199 | 0 | 1 | 0 | F41.8 |  |  |  |  |  |  |  |  |  |
| 202 | 1 | 0 | 1 | M47.816 |  |  |  |  |  |  |  |  |  |
| 203 | 0 | 0 | 1 | M47.12 |  |  |  |  |  |  |  |  |  |
| 207 | 0 | 0 | 5 | I63.9 | M47.82 | G47.30 | E11.42 | Z86.73 |  |  |  |  |  |
| 208 | 1 | 0 | 2 | R00.0 |  |  |  |  |  |  | I72.0 |  |  |
| 210 | 0 | 0 | 0 |  |  |  |  |  |  |  |  |  |  |
| 213 | 1 | 1 | 1 | G90.9 | F90.0 |  |  |  |  |  |  |  |  |
| 221 | 1 | 0 | 1 | B34.9 |  |  |  |  |  |  |  |  |  |
| 225 | 1 | 0 | 2 | M79.7 | R53.82 |  |  |  |  |  |  |  |  |
| 230 | 0 | 0 | 0 |  |  |  |  |  |  |  |  |  |  |
| 234 | 0 | 1 | 1 | G43.909 | F32.9 |  |  |  |  |  |  |  |  |
| 236 | 1 | 0 | 1 | E24.0 |  |  |  |  |  |  |  |  |  |
| 241 | 1 | 3 | 1 | I63.9 | F41.9 | F41.0 | F10.10 |  |  |  |  |  |  |
| 242 | 1 | 1 | 0 | F44.5 |  |  |  |  |  |  |  |  |  |
| 245 | 0 | 0 | 1 | L50.5 |  |  |  |  |  |  |  |  |  |
| 246 | 0 | 0 | 0 |  |  |  |  |  |  |  |  |  |  |
| 256 | 1 | 1 | 1 | F41.9 | R41.3 |  |  |  |  |  |  |  |  |
| 262 | 0 | 0 | 0 |  |  |  |  |  |  |  |  |  |  |
| 277 | 1 | 0 | 2 | M11.80 | D17.9 |  |  |  |  |  |  |  |  |
| 354 | 0 | 0 | 3 | G47.30 | L71.9 | M94.0 |  |  |  |  |  |  |  |
| 361 | 1 | 0 | 1 | Q18.0 |  |  |  |  |  |  |  |  |  |
| 362 | 0 | 1 | 1 | G62.9 | F41.9 |  |  |  |  |  |  |  |  |
| 367 | 1 | 0 | 2 | G90.9 | I95.1 |  |  |  |  |  |  |  |  |
| 383 | 0 | 0 | 1 | R53.83 |  |  |  |  |  |  |  |  |  |
| 398 | 0 | 0 | 0 |  |  |  |  |  |  |  |  |  |  |
| 401 | 0 | 0 | 1 | G70.00 |  |  |  |  |  |  |  |  |  |
| 411 | 1 | 0 | 0 |  |  |  |  |  |  |  |  |  |  |
| 421 | 1 | 2 | 1 | G20 | F32.9 | F42.9 |  |  |  |  |  |  |  |
| 441 | 0 | 0 | 1 | C25.9 |  |  |  |  |  |  |  |  |  |
| 443 | 0 | 1 |  | 0 |  |  |  |  |  | F31.9 |  |  |  |
| 448 | 0 | 0 | 0 |  |  |  |  |  |  |  |  |  |  |
| 450 | 0 | 1 | 0 | F54 |  |  |  |  |  |  |  |  |  |
| 452 | 0 | 0 | 0 |  |  |  |  |  |  |  |  |  |  |
| 453 | 0 | 0 | 0 |  |  |  |  |  |  |  |  |  |  |
| 454 | 0 | 2 | 0 | F50.9 | F41.9 |  |  |  |  |  |  |  |  |
| 455 | 1 | 0 | 1 | K85.9 |  |  |  |  |  |  |  |  |  |
| 464 | 1 | 0 | 1 | B25.9 |  |  |  |  |  |  |  |  |  |
| 466 | 0 | 2 | 0 | F45.9 | F41.9 |  |  |  |  |  |  |  |  |
| 469 | 1 | 0 | 1 | R50.9 |  |  |  |  |  |  |  |  |  |
| 471 | 1 | 0 | 0 |  |  |  |  |  |  |  |  |  |  |
| 472 | 0 | 0 | 1 | L74.4 |  |  |  |  |  |  |  |  |  |
| 481 | 1 | 3 | 0 | F41.9 | F32.9 | F41.0 |  |  |  |  |  |  |  |
| 483 | 1 | 0 | 1 | M62.59 |  |  |  |  |  |  |  |  |  |
| 487 | 0 | 4 | 0 | F34.1 |  |  |  |  |  |  | F31.8 | F43.10 | F10.20 |
| 491 | 0 | 0 | 1 | G11.9 |  |  |  |  |  |  |  |  |  |
| 493 | 0 | 0 | 1 | G70.00 |  |  |  |  |  |  |  |  |  |
| 498 | 1 | 0 | 0 |  |  |  |  |  |  |  |  |  |  |
| 499 | 0 | 1 | 1 | F41.9 | M79.7 |  |  |  |  |  |  |  |  |
| 504 | 1 | 1 | 1 | K21.9 | F41.0 |  |  |  |  |  |  |  |  |
| 506 | 1 | 0 | 1 | B88.2 |  |  |  |  |  |  |  |  |  |
| 512 | 0 | 0 | 2 | D86.9 | B19.20 |  |  |  |  |  |  |  |  |
| 513 | 0 | 0 | 1 | M79.7 |  |  |  |  |  |  |  |  |  |
| 514 | 1 | 0 | 2 | G20 |  |  |  |  |  |  | G90.3 |  |  |
| 515 | 1 | 0 | 2 | E87.2 | K63.89 |  |  |  |  |  |  |  |  |
| 518 | 0 | 0 | 1 | A92.30 |  |  |  |  |  |  |  |  |  |
| 519 | 0 | 0 | 1 | K58.9 |  |  |  |  |  |  |  |  |  |
| 520 | 0 | 0 | 0 |  |  |  |  |  |  |  |  |  |  |
| 525 | 1 | 0 | 1 | G56.21 |  |  |  |  |  |  |  |  |  |
| 527 | 1 | 0 | 0 |  |  |  |  |  |  |  |  |  |  |
| 528 | 1 | 0 | 1 | E2740 |  |  |  |  |  |  |  |  |  |
| 529 | 1 | 0 | 1 | G47.30 |  |  |  |  |  |  |  |  |  |
| 531 | 1 | 0 | 1 | S82.401A |  |  |  |  |  |  |  |  |  |
| 538 | 0 | 1 | 0 | F03.90 |  |  |  |  |  |  |  |  |  |
| 542 | 1 | 0 | 1 | R55 |  |  |  |  |  |  |  |  |  |
| 546 | 1 | 0 | 1 | R15.9 |  |  |  |  |  |  |  |  |  |
| 547 | 1 | 0 | 2 | G43.909 | J34.2 |  |  |  |  |  |  |  |  |
| 549 | 1 | 0 | 1 | K22.70 |  |  |  |  |  |  |  |  |  |
| 559 | 0 | 0 | 0 |  |  |  |  |  |  |  |  |  |  |
| 561 | 0 | 0 | 1 | H53.19 |  |  |  |  |  |  |  |  |  |
| 569 |  | 0 | 0 |  |  |  |  |  |  |  |  |  |  |
| 575 | 1 | 0 | 2 | T78.3 | D47.2 |  |  |  |  |  |  |  |  |
| 595 | 0 | 0 | 1 | C7A.00 |  |  |  |  |  |  |  |  |  |
| 596 | 1 | 0 | 1 | K52.89 |  |  |  |  |  |  |  |  |  |
| 607 | 0 | 0 | 2 | R41.9 | I42.9 |  |  |  |  |  |  |  |  |
| 611 | 1 | 0 | 0 |  |  |  |  |  |  |  |  |  |  |
| 614 | 1 | 0 | 3 | I71.2 | I48.0 | K40 |  |  |  |  |  |  |  |
| 622 | 0 | 0 | 0 |  |  |  |  |  |  |  |  |  |  |
| 629 | 0 | 0 | 3 | G20 | G90.3 |  |  |  |  |  | G90.3 |  |  |
| 632 | 0 | 0 | 0 |  |  |  |  |  |  |  |  |  |  |
| 638 | 0 | 0 | 1 | L23.89 |  |  |  |  |  |  |  |  |  |
| 640 | 0 | 0 | 1 | Q87.2 |  |  |  |  |  |  |  |  |  |
| 641 | 0 | 0 | 1 | G12.21 |  |  |  |  |  |  |  |  |  |
| 644 | 1 | 0 | 1 | K31.84 |  |  |  |  |  |  |  |  |  |
| 649 | 1 | 0 | 1 | K58.0 |  |  |  |  |  |  |  |  |  |
| 650 | 0 | 0 | 0 |  |  |  |  |  |  |  |  |  |  |
| 651 | 1 | 0 | 0 |  |  |  |  |  |  |  |  |  |  |
| 652 | 0 | 0 | 1 | G90.8 |  |  |  |  |  |  |  |  |  |
| 653 | 0 | 0 | 1 | C64.9 |  |  |  |  |  |  |  |  |  |
| 654 | 1 | 0 | 1 | R55 |  |  |  |  |  |  |  |  |  |
| 656 | 0 | 0 | 1 | C07 |  |  |  |  |  |  |  |  |  |
| 662 | 1 | 0 |  | F33 | G90.09 |  |  |  |  |  | G31.84 |  |  |
| 663 | 0 | 1 | 2 |  |  |  |  |  |  |  |  |  |  |
| 665 | 1 | 0 | 2 | K25.9 | K44.9 |  |  |  |  |  |  |  |  |
| 670 | 1 | 1 | 3 | G47.30 | j34.89 |  | F41.9 | R41.3 |  |  |  |  |  |
| 672 | 1 | 0 | 1 | J32.9 |  |  |  |  |  |  |  |  |  |
| 674 | 1 | 0 | 1 | M17.9 |  |  |  |  |  |  |  |  |  |
| 677 | 0 | 0 | 1 | C85.10 |  |  |  |  |  |  |  |  |  |
| 678 | 0 | 0 | 1 | G63.3 |  |  |  |  |  |  |  |  |  |
| 685 | 1 | 0 | 0 |  |  |  |  |  |  |  |  |  |  |
| 686 | 1 | 0 | 2 | D86.9 | K20.0 |  |  |  |  |  |  |  |  |
| 689 | 0 | 0 | 2 | G21 | G62.9 |  |  |  |  |  |  |  |  |
| 690 | 0 | 0 | 0 |  |  |  |  |  |  |  |  |  |  |
| 693 | 0 | 0 | 1 | D86.0. |  |  |  |  |  |  |  |  |  |
| 695 | 1 | 0 | 1 | I31.1 |  |  |  |  |  |  |  |  |  |
| 697 | 0 | 2 | 0 | F60 | F41.9 |  |  |  |  |  |  |  |  |
| 699 | 0 | 0 | 2 | L50.1 | I47.1 |  |  |  |  |  |  |  |  |
| 701 | 0 | 0 | 1 | G12.21 |  |  |  |  |  |  |  |  |  |
| 702 | 0 | 0 | 1 | G90.9 |  |  |  |  |  |  |  |  |  |
| 703 | 0 | 0 | 1 | C91.0 |  |  |  |  |  |  |  |  |  |
| 705 |  | 0 | 0 |  |  |  |  |  |  |  |  |  |  |
| 707 | 0 | 0 | 0 |  |  |  |  |  |  |  |  |  |  |
| 708 | 0 | 0 | 0 |  |  |  |  |  |  |  |  |  |  |
| 709 | 1 | 0 | 1 | M33.22 |  |  |  |  |  |  |  |  |  |
| 711 | 1 | 0 | 0 |  |  |  |  |  |  |  |  |  |  |
| 714 | 0 | 0 | 2 | T88.7XXA | J84.0 |  |  |  |  |  |  |  |  |
| 721 | 1 | 0 | 1 | B27.90 |  |  |  |  |  |  |  |  |  |
| 723 | 0 | 0 | 1 | G04.898 |  |  |  |  |  |  |  |  |  |
| 725 | 1 | 0 | 1 | I88.1 |  |  |  |  |  |  |  |  |  |
| 727 | 0 | 0 | 1 | Q99.9 |  |  |  |  |  |  |  |  |  |
| 729 |  | 0 | 0 |  |  |  |  |  |  |  |  |  |  |
| 730 | 0 | 1 | 1 | G43.909 | F41.9 |  |  |  |  |  |  |  |  |
| 733 | 0 | 0 | 1 | G12.20 |  |  |  |  |  |  |  |  |  |
| 736 | 0 | 0 | 0 |  |  |  |  |  |  |  |  |  |  |
| 738 | 0 | 2 | 0 | F41.9 | F44.4 |  |  |  |  |  |  |  |  |
| 739 | 0 | 0 | 0 |  |  |  |  |  |  |  |  |  |  |
| 741 | 0 | 0 | 0 |  |  |  |  |  |  |  |  |  |  |
| 742 | 0 | 0 | 0 |  |  |  |  |  |  |  |  |  |  |
| 743 | 0 | 0 | 0 |  |  |  |  |  |  |  |  |  |  |
| 747 | 0 | 0 | 0 |  |  |  |  |  |  |  |  |  |  |
| 748 | 0 | 0 | 0 |  |  |  |  |  |  |  |  |  |  |
| 751 | 0 | 0 | 1 | J84.10 |  |  |  |  |  |  |  |  |  |
| 767 | 1 | 0 | 1 | K58.9 |  |  |  |  |  |  |  |  |  |
| 769 | 1 | 0 | 0 |  |  |  |  |  |  |  |  |  |  |
| 770 | 0 | 0 | 3 | N53.12 | G47.11 | G47.419 |  |  |  |  |  |  |  |
| 771 | 0 | 1 | 1 | F42 | T73.3XXA |  |  |  |  |  |  |  |  |
| 782 | 1 | 0 | 2 | R55 |  |  |  |  |  |  | G31.84 |  |  |
| 785 | 1 | 0 | 1 | I63.9 |  |  |  |  |  |  |  |  |  |
| 789 | 1 | 0 | 1 | M31.6 |  |  |  |  |  |  |  |  |  |
| 790 | 0 | 0 | 0 |  |  |  |  |  |  |  |  |  |  |
| 791 | 1 | 0 | 1 | K85.3 |  |  |  |  |  |  |  |  |  |
| 792 | 1 | 0 | 0 |  |  |  |  |  |  |  |  |  |  |
| 793 | 0 | 0 | 0 |  |  |  |  |  |  |  |  |  |  |
| 794 | 0 | 0 | 1 | D61 |  |  |  |  |  |  |  |  |  |
| 796 | 0 | 0 | 0 |  |  |  |  |  |  |  |  |  |  |
| 803 |  | 0 | 1 | G30.9 |  |  |  |  |  |  |  |  |  |
| 809 | 0 | 0 | 2 | G03.9 | A69.22 |  |  |  |  |  |  |  |  |
| 812 | 0 | 1 | 1 | S06.9XOA | F79 |  |  |  |  |  |  |  |  |
| 813 | 0 | 0 | 1 | L23 |  |  |  |  |  |  |  |  |  |
| 815 | 0 | 0 | 1 | R53.82 |  |  |  |  |  |  |  |  |  |
| 816 | 0 | 0 | 0 |  |  |  |  |  |  |  |  |  |  |
| 825 | 0 | 0 | 0 |  |  |  |  |  |  |  |  |  |  |
| 830 | 1 | 0 | 0 |  |  |  |  |  |  |  |  |  |  |
| 838 | 1 | 0 | 1 | J01.40 |  |  |  |  |  |  |  |  |  |
| 842 | 0 | 2 | 0 | F41.1 | f40.01 |  |  |  |  |  |  |  |  |
| 847 | 1 | 0 | 2 | N30.20 | N20.0 |  |  |  |  |  |  |  |  |
| 856 |  | 0 | 2 |  | G14 | E53.8 |  |  |  |  |  |  |  |
| 858 | 0 | 0 | 1 | M79.1 |  |  |  |  |  |  |  |  |  |
| 863 | 0 | 0 | 0 |  |  |  |  |  |  |  |  |  |  |
| 870 | 0 | 0 | 1 | K86.1 |  |  |  |  |  |  |  |  |  |
| 871 | 1 | 0 | 1 | D13.4 |  |  |  |  |  |  |  |  |  |
| 874 | 0 | 1 | 1 | F44.4 | N25.81 |  |  |  |  |  |  |  |  |
| 880 | 0 | 0 | 0 |  |  |  |  |  |  |  |  |  |  |
| 882 | 0 | 0 | 0 |  |  |  |  |  |  |  |  |  |  |
| 890 | 0 | 3 | 0 | F44.7 | F51.01 | F43.12 |  |  |  |  |  |  |  |
| 899 | 0 | 0 | 0 |  |  |  |  |  |  |  |  |  |  |
| 903 | 1 | 0 | 1 | T44.3X1A |  |  |  |  |  |  |  |  |  |
| 911 | 0 | 0 | 2 | H51.8 | M79.7 |  |  |  |  |  |  |  |  |
| 914 | 0 | 0 | 0 |  |  |  |  |  |  |  |  |  |  |
| 920 | 0 | 2 | 0 | F41.9 | F33 |  |  |  |  |  |  |  |  |
| 929 | 0 | 0 | 4 | G47.30 | M47.812 | M79.604 | M48.06 |  |  |  |  |  |  |
| 935 | 0 | 0 | 2 | G90.8 | E11.43 |  |  |  |  |  |  |  |  |
| 954 | 1 | 1 | 0 | F51.01 |  |  |  |  |  |  |  |  |  |
| 959 | 0 | 1 | 0 | F03.90 |  |  |  |  |  |  |  |  |  |
| 968 | 0 | 0 | 2 |  | G47.33 | J44.9 |  |  |  |  |  |  |  |
| 972 | 1 | 0 | 2 |  | R61 | M25.56 |  |  |  |  |  |  |  |
| 977 | 0 | 0 | 0 |  |  |  |  |  |  |  |  |  |  |
| 1087 |  | 0 | 0 |  |  |  |  |  |  |  |  |  |  |

**Data File C**

**Codebook for Data File C:**

The following data elements were extracted from components of a REDCap database. These were entered from responses to sections of the Home Health Questionnaire (HHQ) concerning disability and quality of life associated with the current illness.

Variable Description and Analysis Notes

record_id number (38-1087) used to link (merge) data for each of n=247 patients presenting to the Emory Special Diagnostic Service clinic from Feb. 7, 2014 and May 31, 2017

4 yes/no HHQ items asking about the impact of this illness, each coded 1=yes, 2=no, 3=not asked, 4=left blank:

quit_work had to quit working because of this illness

cut_work had to cut back on number of work hours due to this illness

apply_disab felt it necessary to apply for disability benefits due to this illness

get_disab receiving disability benefits because of this illness

prof_family whether this illness has affected professional or family well being

any_qlesq 1=yes, 2=no, 3=not asked, 4=left blank, indicating whether the Quality of Life Enjoyment and Satisfaction Questionnaire – Short Form (Q-LES-Q-SF) section of the HHQ was answered.

If so, each item is answered with 1=very poor, 2=poor, 3=fair, 4=good, 5=very good for “taking everything into consideration, during the past week how satisfied have you been with your…”

qlesq_1 physical health

qlesq_2 mood

qlesq_3 work

qlesq_4 household activities

qlesq_5 social relationships

qlesq_6 family relationships

qlesq_7 leisure time activities

qlesq_8 ability to function in daily life

qlesq_9 sexual drive, interest, and/or performance

qlesq_10 economic status

qlesq_11 living/housing situation

qlesq_12 ability to get around physically without dizziness/unsteady/falling

qlesq_13 vision in terms of ability to do work or hobbies

qlesq_14 overall sense of well being

qlesq_15 medication (if taking any)

qlesq_16 overall life satisfaction and contentment

NOTE: Per the authors of this instrument, the Q-LES-Q-SF score is the sum of items 1-14, expressed as a percentage of the maximum possible score for the number of items answered; the score is not compute if more than 2 of these 14 items are missing.

any_wsas 1=yes, 2=no, 3=not asked indicating whether the Work and Social Adjustment Scale (WSAS) section of the HHQ was answered.

If so, each item is answered on a 0-8 scale where 0=not at all, 2=slightly, 4=definitely, 6=markedly, and 8=very severely in response to “determine on the scale provided how much your problem impairs your ability to carry out the activity.”

wsas_1 work

wsas_2 home/household management

wsas_3 social leisure activities

wsas_4 private leisure activities

wsas_5 family and other relationships

NOTE: The total WSAS score, with a possible range of 0-40, is computed as the sum of the above 5 illness impact items. If item 1 (work) was not answered it was filled in with 6 if the previous item asking whether the patient had ever cut back on work hours due to this illness was answered “yes,” or with 8 if the previous item asking whether the patient had ever quit work due to this illness was answered “yes.” If only one of the 5 WSAS items was still missing it was filled in with the mean of the remaining 4 items. If more than one item was missing, the WSAS score was not computed.

**Data File C**

| **File C**  record_id | quit_work | cut_work | apply_disab | get_disab | prof_family | any_qlesq | qlesq_1 | qlesq_2 | qlesq_3 | qlesq_4 | qlesq_5 | qlesq_6 | qlesq_7 | qlesq_8 | qlesq_9 | qlesq_10 | qlesq_11 | qlesq_12 | qlesq_13 | qlesq_14 | qlesq_15 | qles_16 | any_wsas | wsas_1 | wsas_2 | wsas_3 | wsas_4 | wsas_5 |
| --- | --- | --- | --- | --- | --- | --- | --- | --- | --- | --- | --- | --- | --- | --- | --- | --- | --- | --- | --- | --- | --- | --- | --- | --- | --- | --- | --- | --- |
| 38 | 4 | 4 | 4 | 4 | 4 | 1 | 2 | 2 | 2 | 3 | 3 | 3 | 2 | 2 | 2 | 4 | 4 | 2 | 2 | 2 | 2 | 1 | 1 | 6 | 5 | 8 | 6 | 6 |
| 39 | 4 | 4 | 4 | 4 | 4 | 1 | 2 | 3 | 6 | 2 | 3 | 3 | 1 | 2 | 2 | 2 | 5 | 4 | 4 | 2 | 3 | 3 | 1 | 8 | 6 | 6 | 7 | 5 |
| 43 | 4 | 4 | 4 | 4 | 4 | 1 | 3 | 5 | 5 | 3 | 4 | 5 | 3 | 5 | 2 | 3 | 5 | 5 | 4 | 5 | 6 | 2 | 1 | 0 | 1 | 0 | 0 | 0 |
| 46 | 1 | 1 | 1 | 2 | 1 | 1 | 1 | 1 | 1 | 1 | 3 | 5 | 1 | 1 | 1 | 5 | 5 | 3 | 1 | 1 | 3 | 1 | 1 | 9 | 8 | 8 | 7 | 5 |
| 47 | 2 | 2 | 2 | 2 | 2 | 1 | 5 | 5 | 6 | 5 | 4 | 5 | 5 | 5 | 6 |  | 5 | 2 | 5 | 5 | 5 | 5 | 1 | 9 | 0 | 7 | 6 | 4 |
| 49 | 2 | 2 | 2 | 2 | 2 | 1 | 3 | 4 | 5 | 2 | 5 | 4 | 5 | 5 | 1 | 3 | 4 | 5 | 4 | 4 | 3 | 4 | 1 | 6 | 5 | 5 | 2 | 1 |
| 54 | 2 | 2 | 2 | 2 | 1 | 1 | 2 | 2 | 1 | 1 | 3 | 5 | 1 | 1 | 1 | 4 | 5 | 3 | 1 | 2 | 1 | 1 | 1 | 6 | 4 | 2 | 2 | 1 |
| 55 | 2 | 2 | 2 | 2 | 1 | 1 | 2 | 3 | 2 | 2 | 3 | 3 | 2 | 3 | 2 | 3 | 3 | 2 | 3 | 2 | 3 | 3 | 2 |  |  |  |  |  |
| 58 | 2 | 2 | 2 | 2 | 2 | 3 |  |  |  |  |  |  |  |  |  |  |  |  |  |  |  |  | 3 |  |  |  |  |  |
| 65 | 1 | 1 | 3 | 2 | 1 | 1 | 1 | 4 | 1 | 1 | 1 | 4 | 2 | 1 | 1 | 3 | 4 | 3 | 4 | 2 | 3 | 1 | 1 | 8 | 7 | 7 | 6 | 6 |
| 69 | 2 | 1 | 2 | 2 | 1 | 1 | 1 | 2 | 2 | 1 | 1 | 2 | 1 | 1 | 1 | 3 | 3 | 2 | 1 | 1 | 1 | 1 | 1 | 7 | 8 | 8 | 8 | 8 |
| 72 | 2 | 1 | 2 | 2 | 1 | 1 | 1 | 3 | 3 | 2 | 2 | 3 | 2 | 2 | 6 | 4 | 5 | 4 | 2 | 3 | 2 | 2 | 1 | 4 | 4 | 4 | 2 | 4 |
| 73 | 1 | 1 | 2 | 2 | 1 | 1 | 3 | 4 | 6 | 3 | 4 | 4 | 6 | 3 | 3 | 4 | 4 | 4 | 4 | 4 | 3 | 4 | 1 | 6 | 4 | 2 | 4 | 0 |
| 74 | 2 | 2 | 2 | 2 | 2 | 1 | 4 | 3 | 6 | 4 | 4 | 5 | 3 | 3 | 2 | 3 | 4 | 4 | 4 | 4 | 4 | 4 | 1 | 3 | 0 | 2 | 4 | 1 |
| 76 | 1 | 1 | 2 | 2 | 1 | 1 | 3 | 3 | 4 | 4 | 4 | 4 |  | 3 | 2 | 2 | 3 | 2 | 3 | 3 | 4 | 3 | 1 | 7 | 4 | 4 | 4 | 3 |
| 78 | 1 | 1 | 2 | 2 | 1 | 1 | 2 | 2 |  | 3 | 4 | 5 | 3 | 3 | 2 | 3 | 5 | 1 | 2 | 2 | 1 | 3 | 1 | 8 | 8 | 8 | 8 |  |
| 79 | 2 | 1 | 1 | 1 | 1 | 1 | 2 | 2 | 1 | 1 | 5 | 5 | 2 | 1 | 1 |  | 2 | 2 | 2 | 2 | 3 | 2 | 1 | 8 | 8 | 8 | 4 | 0 |
| 80 | 2 | 2 | 2 | 2 | 2 | 1 | 3 | 3 | 3 | 3 | 3 | 3 | 3 | 1 | 1 | 3 | 3 | 3 | 3 | 3 | 6 | 3 | 1 | 6 | 9 | 0 | 0 | 2 |
| 84 | 4 | 4 | 4 | 4 | 4 | 3 |  |  |  |  |  |  |  |  |  |  |  |  |  |  |  |  | 3 |  |  |  |  |  |
| 85 | 1 | 1 | 2 | 2 | 2 | 1 | 3 | 2 | 4 | 2 | 4 | 4 | 1 | 4 | 2 | 5 | 4 | 2 | 5 | 3 | 4 | 5 | 1 | 7 | 4 | 6 | 5 | 2 |
| 87 | 1 | 2 | 2 | 2 | 2 | 1 | 1 | 1 | 1 | 1 | 1 | 5 | 2 | 1 | 4 | 6 | 5 | 2 | 2 | 2 |  | 2 | 1 | 7 | 7 | 7 | 7 | 0 |
| 91 | 2 | 1 | 2 | 2 | 2 | 1 | 4 | 3 | 3 | 3 | 3 | 4 | 3 | 3 | 1 | 5 | 5 | 5 | 4 | 3 | 5 | 3 | 1 | 4 | 3 | 4 | 4 | 3 |
| 92 | 2 | 2 | 2 | 2 | 1 | 1 | 1 | 1 | 1 | 1 | 1 | 3 | 1 | 1 | 2 | 5 | 5 | 5 | 1 | 2 |  | 2 | 1 | 7 | 7 | 7 | 7 | 7 |
| 93 | 2 | 2 | 2 | 2 | 2 | 1 | 1 | 4 | 4 | 4 | 4 | 4 |  | 1 | 3 | 3 | 5 | 5 | 5 | 4 | 3 | 4 | 1 | 0 | 0 | 0 | 0 | 0 |
| 94 | 1 | 1 | 2 | 2 | 1 | 3 |  |  |  |  |  |  |  |  |  |  |  |  |  |  |  |  | 3 |  |  |  |  |  |
| 96 | 1 | 1 | 2 | 2 | 1 | 1 | 1 | 3 | 3 | 3 | 4 | 5 | 2 | 3 | 3 | 3 | 3 | 1 | 3 | 3 | 3 | 2 | 1 | 9 | 4 | 4 | 4 | 1 |
| 99 | 2 | 2 | 2 | 2 | 1 | 1 | 2 | 2 | 6 | 2 | 2 | 3 | 1 | 1 |  | 3 | 3 | 1 | 4 | 2 | 3 | 1 | 1 | 8 | 6 | 5 | 7 | 5 |
| 105 | 4 | 4 | 4 | 4 | 4 | 1 | 3 | 4 | 4 | 3 |  | 5 | 4 | 4 | 2 | 4 | 4 | 3 | 2 | 3 | 3 | 2 | 2 |  |  |  |  |  |
| 107 | 2 | 2 | 2 | 2 | 1 | 1 | 2 | 2 | 2 | 2 | 1 | 2 | 2 | 2 | 2 | 5 | 5 | 3 | 2 | 1 | 1 | 1 | 1 | 6 | 5 | 8 | 8 | 7 |
| 110 | 2 | 2 | 2 | 2 | 1 | 1 | 3 | 4 | 3 | 2 | 4 | 4 | 3 | 2 | 3 | 4 | 4 | 2 | 4 | 4 | 3 | 2 | 1 | 4 | 7 | 6 | 4 | 3 |
| 111 | 4 | 4 | 4 | 4 | 4 | 1 | 2 | 4 | 6 | 4 | 4 | 4 | 2 | 3 | 4 | 4 | 4 | 2 | 4 | 4 | 4 | 3 | 1 | 8 | 7 | 6 | 7 | 3 |
| 113 | 2 | 2 | 2 | 2 | 1 | 1 | 3 | 2 | 6 | 4 | 3 | 4 | 3 | 3 | 3 | 5 | 5 | 3 | 3 | 2 | 3 | 3 | 1 | 9 | 4 | 4 | 4 | 2 |
| 115 | 2 | 1 | 2 | 2 | 1 | 1 | 2 | 3 | 2 | 2 | 3 | 3 | 2 | 2 | 2 | 4 | 4 | 3 | 4 | 2 | 3 | 3 | 1 | 6 | 6 | 4 | 4 | 3 |
| 116 | 1 | 1 | 2 | 2 | 1 | 1 | 1 | 1 | 6 | 1 | 1 | 3 | 1 | 1 | 1 | 4 | 4 | 1 | 1 | 1 | 1 | 1 | 1 | 8 | 8 | 8 | 8 | 7 |
| 120 | 2 | 2 | 2 | 2 | 1 | 1 | 1 | 1 | 6 | 1 | 2 | 5 | 3 | 3 | 1 | 2 | 3 | 3 | 4 | 2 | 1 | 1 | 1 | 8 | 8 | 8 | 8 | 0 |
| 121 | 2 | 2 | 2 | 2 | 1 | 1 | 3 | 3 | 3 | 4 | 4 | 5 | 1 | 2 | 3 | 5 | 5 | 1 | 4 | 3 | 3 | 2 | 1 | 6 | 2 | 6 | 6 |  |
| 122 | 2 | 1 | 2 | 2 | 1 | 1 | 2 | 2 | 2 | 2 | 2 | 3 | 2 | 2 | 2 | 2 | 3 | 4 | 4 |  |  | 2 | 1 | 8 | 2 | 2 | 4 | 3 |
| 124 | 4 | 4 | 4 | 4 | 4 | 1 | 1 | 2 | 4 | 3 | 5 | 5 | 1 | 4 | 4 | 4 | 5 | 5 | 4 | 2 | 3 | 2 | 1 | 3 | 3 | 2 | 8 | 3 |
| 125 | 1 | 1 | 2 | 2 | 1 | 1 | 2 | 2 | 2 | 3 | 4 | 5 | 3 | 2 | 3 | 5 | 5 | 4 | 4 | 2 | 6 | 2 | 1 | 6 | 5 | 6 | 5 | 2 |
| 126 | 2 | 2 | 2 | 2 | 1 | 1 | 2 | 3 |  | 3 | 4 | 4 | 3 | 3 | 2 |  | 5 | 3 | 4 | 3 | 4 | 3 | 3 |  |  |  |  |  |
| 127 | 4 | 4 | 4 | 4 | 4 | 1 | 4 | 4 | 4 | 5 | 5 | 5 | 5 | 4 |  | 4 | 4 | 4 | 5 | 4 | 4 | 4 | 1 | 9 | 0 | 0 | 0 | 0 |
| 128 | 1 | 1 | 2 | 2 | 1 | 1 | 1 | 2 | 6 | 2 | 3 | 3 | 3 | 2 | 1 | 3 | 4 | 2 | 2 | 2 | 5 | 2 | 1 | 9 | 7 | 7 | 7 | 3 |
| 129 | 4 | 4 | 4 | 4 | 4 | 1 | 1 | 2 | 3 | 4 | 5 | 5 | 3 | 2 | 1 | 5 | 4 | 3 | 5 | 2 | 4 | 1 | 1 | 7 | 3 | 7 | 2 | 4 |
| 130 | 2 | 1 | 2 | 2 | 1 | 1 | 2 | 2 | 2 | 2 | 2 | 4 | 2 | 2 | 2 | 4 | 4 | 3 | 3 | 2 | 6 | 2 | 1 | 7 | 6 | 8 | 8 | 6 |
| 131 | 2 | 2 | 2 | 2 | 1 | 1 | 1 | 2 | 6 | 3 | 3 | 3 | 1 | 1 | 1 | 4 | 5 | 2 | 3 | 2 | 1 | 2 | 1 | 8 | 5 | 6 | 4 | 4 |
| 132 | 2 | 2 | 2 | 2 | 1 | 1 | 3 | 3 | 3 | 3 |  | 4 | 3 | 3 | 2 | 4 | 3 | 3 | 3 | 3 | 3 | 3 | 1 | 4 | 4 | 4 | 5 | 2 |
| 133 |  |  |  |  |  | 1 | 3 | 2 | 3 | 3 | 3 | 3 | 2 | 3 | 3 | 3 | 4 | 3 | 3 | 3 | 3 | 3 | 1 | 5 | 5 | 6 | 2 | 3 |
| 134 | 1 | 1 | 2 | 2 | 1 | 1 | 2 | 2 | 6 | 2 | 3 |  |  | 2 | 2 | 3 | 4 | 4 | 4 | 2 | 3 | 2 | 1 | 9 | 3 | 4 | 2 | 0 |
| 135 | 1 | 1 | 2 | 2 | 1 | 1 | 1 | 2 | 1 | 1 | 2 | 3 | 1 | 1 | 2 | 1 | 4 | 1 | 5 | 2 | 2 | 1 | 1 | 8 | 6 | 6 | 7 | 6 |
| 136 | 2 | 1 | 2 | 2 | 3 | 1 | 3 | 3 | 3 | 3 | 3 | 3 | 3 | 5 |  | 4 | 3 | 5 | 3 | 3 | 3 | 3 | 1 | 4 | 4 | 4 | 4 | 0 |
| 137 | 2 | 1 | 2 | 2 | 2 | 1 | 3 | 5 | 3 | 3 | 4 | 4 | 4 | 4 | 4 | 5 | 5 | 4 | 4 | 4 | 4 | 4 | 1 | 3 | 2 | 0 | 2 | 0 |
| 138 | 2 | 2 | 2 | 2 | 2 | 1 | 1 | 3 | 1 | 1 | 4 | 5 | 1 | 3 | 3 | 5 | 5 | 5 | 5 | 2 | 4 | 2 | 1 | 8 | 8 | 8 | 8 | 0 |
| 139 | 1 | 1 | 1 | 1 | 1 | 1 | 3 | 3 | 1 | 1 | 1 | 4 | 1 | 1 | 6 | 1 | 3 | 2 | 2 | 2 | 2 | 2 | 1 | 8 | 8 | 8 | 6 | 4 |
| 140 | 2 | 2 | 2 | 2 | 1 | 1 | 2 | 3 | 6 | 3 | 3 | 4 | 3 | 3 | 3 | 2 | 3 | 5 | 4 | 4 | 3 | 3 | 1 | 9 | 2 | 4 | 2 | 2 |
| 142 | 2 | 2 | 2 | 2 | 2 | 1 | 2 | 3 | 3 | 3 |  | 5 | 4 | 3 | 3 | 4 | 5 | 5 | 4 | 3 | 4 | 4 | 1 | 4 | 0 | 0 | 4 | 0 |
| 146 | 2 | 1 | 2 | 2 | 2 | 1 | 1 | 2 | 6 | 4 | 4 | 4 | 4 | 3 | 6 | 3 | 4 | 3 | 2 | 2 | 4 | 3 | 1 | 9 | 7 | 4 | 4 | 2 |
| 152 | 2 | 2 | 2 | 2 | 1 | 1 | 3 | 3 | 3 | 3 | 4 | 5 | 4 | 4 | 4 | 4 | 4 | 4 | 4 | 2 | 6 | 3 | 1 | 3 | 3 | 3 | 3 | 2 |
| 159 | 2 | 2 | 2 | 2 | 1 | 1 | 2 | 2 | 3 | 3 | 2 | 1 | 1 | 2 | 1 | 4 | 4 | 5 | 5 | 2 | 4 | 2 | 1 | 3 | 3 | 7 | 3 | 6 |
| 160 | 2 | 1 | 2 | 2 | 1 | 1 | 2 | 4 | 6 | 2 | 4 | 4 | 2 | 3 | 6 | 5 | 5 | 2 | 4 | 3 | 2 | 3 | 1 | 6 | 4 | 6 | 7 | 3 |
| 162 | 2 | 2 | 1 | 2 | 1 | 1 | 1 | 1 | 2 | 2 | 2 | 2 | 1 | 1 | 2 | 4 | 4 | 1 | 1 | 2 | 2 | 2 | 1 | 4 | 6 | 6 | 7 | 6 |
| 164 | 2 | 2 | 2 | 2 | 1 | 1 | 4 | 5 | 5 | 5 | 5 | 5 | 5 | 5 |  | 4 | 5 | 5 | 4 | 4 | 4 | 4 | 1 | 9 | 0 | 0 | 0 | 0 |
| 165 | 2 | 1 | 2 | 2 | 1 | 1 | 2 | 1 | 3 | 2 | 2 | 2 | 2 | 2 | 1 | 3 | 3 | 3 | 3 | 2 | 2 | 2 | 1 | 4 | 4 | 4 | 3 | 6 |
| 166 | 1 | 1 | 2 | 2 | 1 | 1 | 2 | 2 | 1 | 1 | 2 | 2 | 2 | 2 | 1 | 2 | 1 | 1 | 1 | 1 | 2 | 1 | 1 | 8 | 6 | 6 | 6 | 3 |
| 171 | 2 | 2 | 2 | 2 | 1 | 1 | 1 | 1 | 1 | 1 | 1 | 1 | 1 | 1 | 1 | 1 | 1 | 1 | 1 | 1 | 6 | 1 | 1 | 9 | 8 | 8 | 8 | 8 |
| 173 | 2 | 2 | 2 | 2 | 1 | 1 | 4 | 4 | 1 | 4 | 5 | 4 | 4 | 5 | 5 | 4 | 5 | 5 | 3 | 4 | 4 | 4 | 1 | 4 | 4 | 4 | 4 | 1 |
| 183 | 2 | 1 | 2 | 2 | 1 | 1 | 3 | 3 | 3 | 4 | 4 | 4 | 4 | 4 | 4 | 3 | 5 | 5 | 4 | 4 | 4 | 4 | 1 | 4 | 2 | 3 | 2 | 1 |
| 185 | 2 | 1 | 2 | 2 | 1 | 1 | 2 | 3 | 2 | 2 | 2 | 5 | 2 | 2 | 6 | 2 | 4 | 2 | 5 | 3 | 2 | 2 | 1 |  | 4 | 0 | 6 | 2 |
| 190 | 2 | 1 | 1 | 2 | 1 | 1 | 2 | 2 | 4 | 2 | 2 | 4 | 1 | 2 | 3 | 4 | 4 | 2 | 2 | 2 | 3 | 2 | 1 | 6 | 4 | 8 | 8 | 4 |
| 191 | 2 | 2 | 2 | 2 | 1 | 1 | 2 | 2 | 2 | 2 | 3 | 3 | 2 | 3 | 6 | 3 | 3 | 2 | 3 | 2 | 3 | 2 | 1 | 3 | 5 | 5 | 2 | 2 |
| 198 | 2 | 2 | 2 | 2 | 1 | 1 | 2 | 2 | 2 | 2 | 2 | 2 | 2 | 2 | 2 | 4 | 4 | 4 | 4 | 2 | 4 | 2 | 1 | 2 | 2 | 2 | 2 | 2 |
| 199 | 1 | 1 | 1 | 2 | 1 | 1 | 2 | 3 | 1 | 3 | 5 | 5 | 4 | 2 | 3 | 3 | 4 | 3 | 5 | 4 | 4 | 3 | 1 | 8 | 4 | 4 | 2 | 2 |
| 202 | 2 | 2 | 2 | 2 | 1 | 1 | 4 | 3 | 2 | 4 | 4 | 4 | 4 | 4 | 4 | 4 | 4 | 4 | 4 | 4 | 6 | 4 | 1 | 2 | 4 | 2 | 4 | 4 |
| 203 | 1 | 1 | 1 | 2 | 1 | 1 | 1 | 2 | 2 | 2 | 2 | 2 | 2 | 2 | 1 | 4 | 4 | 2 | 1 | 2 | 5 | 2 | 1 | 8 | 8 | 8 | 7 | 4 |
| 207 | 2 | 1 | 1 | 2 | 1 | 1 | 1 | 3 | 6 | 1 | 1 | 4 | 2 | 1 | 1 | 3 | 4 | 1 | 3 | 1 | 1 | 3 | 1 | 0 | 2 | 0 | 2 | 9 |
| 208 | 2 | 1 | 2 | 2 | 1 | 1 | 2 | 2 | 3 | 2 | 4 | 4 | 4 | 2 | 4 | 4 | 4 | 2 | 3 | 2 | 6 | 2 | 1 | 6 | 6 | 5 | 6 | 3 |
| 210 | 2 | 1 | 2 | 2 | 1 | 1 | 1 | 3 | 3 | 2 | 2 | 4 | 2 | 1 | 1 | 4 | 4 | 2 | 3 | 3 | 2 | 2 | 1 | 0 | 2 | 6 | 6 | 2 |
| 213 | 2 | 2 | 2 | 2 | 1 | 1 | 1 | 3 | 4 | 4 | 3 | 3 | 2 | 2 | 6 | 3 | 3 | 2 | 3 | 3 | 4 | 4 | 1 | 9 |  | 5 | 2 | 3 |
| 221 | 2 | 2 | 2 | 2 | 1 | 1 | 1 | 3 | 4 | 3 | 4 | 5 | 3 | 2 | 4 | 4 | 5 | 2 | 5 | 2 | 6 | 2 | 2 |  |  |  |  |  |
| 225 | 1 | 1 | 1 | 2 | 1 | 1 | 2 | 4 | 1 | 2 | 3 | 3 | 2 | 2 | 1 | 3 | 4 | 4 | 4 | 3 | 3 | 3 | 1 | 8 | 6 | 7 | 7 | 5 |
| 230 | 2 | 1 | 2 | 2 | 1 | 1 | 2 | 5 | 5 | 5 | 5 | 5 | 5 | 5 | 5 | 5 | 5 | 5 | 5 | 5 |  | 5 | 1 | 4 | 0 | 4 | 0 | 0 |
| 234 | 2 | 1 | 2 | 2 | 1 | 1 | 2 | 3 | 3 | 2 | 3 | 4 | 3 | 2 | 1 | 4 | 4 | 1 | 3 | 3 | 4 | 2 | 1 | 4 | 6 | 8 | 3 | 4 |
| 236 | 1 | 1 | 1 | 2 | 1 | 1 | 1 | 2 | 1 | 1 | 3 | 2 | 1 | 2 | 2 | 3 | 5 | 4 | 5 | 2 | 2 | 2 | 1 | 8 | 7 | 7 | 7 | 3 |
| 241 | 2 | 2 | 2 | 2 | 2 | 1 | 2 | 2 | 4 | 4 | 4 | 4 | 4 | 3 | 1 | 5 | 5 | 4 | 4 | 2 | 6 | 1 | 1 | 0 | 0 | 3 | 3 | 1 |
| 242 | 2 | 1 | 1 | 2 | 1 | 1 | 2 | 2 | 1 | 1 | 3 | 4 |  |  | 2 | 1 | 4 | 3 | 2 | 3 | 3 | 3 | 1 | 9 | 4 | 0 | 0 | 2 |
| 245 | 2 | 2 | 2 | 2 | 2 | 1 | 2 | 5 | 4 | 5 | 5 | 5 | 5 | 5 | 5 | 5 | 5 | 5 | 5 | 5 | 5 | 5 | 1 | 3 | 0 | 2 | 0 | 0 |
| 246 | 2 | 1 | 2 | 2 | 1 | 1 | 3 | 2 | 2 | 2 | 3 | 3 | 3 | 3 | 3 | 3 | 3 | 3 | 3 | 2 | 3 | 2 | 1 | 4 | 4 | 6 | 3 | 7 |
| 256 | 2 | 1 | 2 | 2 | 1 | 1 | 3 | 2 | 2 | 3 | 3 | 3 | 3 | 2 | 3 | 4 | 3 | 2 | 2 | 2 | 5 | 3 | 1 | 6 | 7 | 7 | 7 | 4 |
| 262 | 2 | 1 | 2 | 2 | 1 | 1 | 2 | 3 | 3 | 3 | 4 | 4 | 2 | 3 | 1 | 4 | 4 | 4 | 3 | 2 | 3 | 3 | 1 | 6 | 3 | 4 | 5 | 1 |
| 277 | 2 | 2 | 2 | 2 | 1 | 1 | 4 | 3 | 6 | 3 | 3 | 4 | 3 | 3 | 3 | 5 | 5 | 4 | 3 | 3 |  | 3 | 1 | 9 | 3 | 4 | 4 | 4 |
| 354 | 2 | 1 | 2 | 2 | 1 | 1 | 2 | 3 | 2 | 2 | 2 | 3 | 2 | 2 | 2 | 4 | 4 | 4 | 4 | 2 | 3 | 2 | 1 | 6 | 6 | 7 | 6 | 2 |
| 361 | 2 | 2 | 2 | 2 | 2 | 1 | 3 | 4 | 4 | 4 | 4 | 4 | 3 | 4 | 4 | 4 | 4 | 5 | 4 | 5 | 6 | 4 | 1 | 0 | 0 | 0 | 0 | 0 |
| 362 | 2 | 2 | 2 | 2 | 1 | 1 | 2 | 2 | 4 | 3 | 3 | 4 | 3 | 3 | 3 | 4 | 4 | 3 | 5 | 2 | 4 | 2 | 1 | 2 | 2 | 3 | 4 | 0 |
| 367 | 1 | 1 | 2 | 2 | 1 | 1 | 2 | 3 | 6 | 2 | 2 | 3 | 2 | 2 | 3 | 5 | 5 | 3 |  | 2 |  | 2 | 1 | 9 | 6 | 6 | 6 | 6 |
| 383 | 1 | 1 | 1 | 2 | 1 | 1 | 2 | 2 | 1 | 2 | 1 | 3 | 1 | 2 | 2 | 2 | 4 | 3 | 4 | 2 | 4 | 2 | 1 | 8 | 7 | 8 | 4 | 4 |
| 398 | 2 | 2 | 2 | 2 | 2 | 1 | 4 | 4 | 4 | 4 | 4 | 4 | 4 | 5 | 6 | 4 | 4 | 5 | 4 | 4 | 3 | 4 | 1 | 9 | 3 | 3 | 3 | 4 |
| 401 | 1 | 1 | 1 | 2 | 1 | 1 | 3 | 4 | 4 | 4 | 4 | 4 | 4 | 3 | 3 | 5 | 4 | 4 | 5 | 4 | 4 | 4 | 1 | 8 | 4 | 4 | 6 | 2 |
| 411 | 2 | 1 | 2 | 2 | 2 | 1 | 5 | 5 | 5 | 5 | 5 | 5 | 4 | 5 | 5 | 3 | 5 | 5 | 5 | 5 | 5 | 5 | 1 | 2 | 0 | 0 | 0 | 0 |
| 421 | 1 | 1 | 1 | 2 | 1 | 3 |  |  |  |  |  |  |  |  |  |  |  |  |  |  |  |  | 3 |  |  |  |  |  |
| 441 | 2 | 1 | 2 | 2 | 1 | 1 | 1 | 3 | 4 | 1 | 3 | 3 | 1 | 1 | 1 | 5 | 5 | 3 | 5 | 1 | 3 | 2 | 1 | 2 | 6 | 4 | 4 | 4 |
| 443 | 1 | 1 | 2 | 2 | 1 | 1 | 3 | 3 | 3 | 3 | 2 | 3 | 3 | 4 | 3 | 3 | 4 | 3 | 4 | 3 | 4 | 3 | 1 | 9 | 4 | 4 | 4 | 3 |
| 448 | 1 | 1 | 2 | 2 | 2 | 1 | 2 | 3 | 2 | 2 | 3 | 4 | 1 | 2 | 6 | 4 | 4 | 3 | 4 | 2 | 6 | 2 | 1 | 8 | 6 | 4 | 6 | 2 |
| 450 | 1 | 1 | 1 | 2 | 1 | 1 | 1 | 3 | 1 | 1 | 1 | 3 | 1 | 1 | 1 | 1 | 4 | 2 | 2 | 1 | 3 | 1 | 1 | 8 | 8 | 8 | 6 | 5 |
| 452 | 2 | 2 | 2 | 2 | 2 | 1 | 2 | 2 | 2 | 2 | 2 | 2 | 2 | 2 | 5 | 5 | 5 | 5 | 5 | 3 | 6 | 2 | 1 | 0 | 0 | 3 | 0 | 0 |
| 453 | 2 | 2 | 2 | 2 | 1 | 1 | 2 | 2 | 2 | 2 | 1 | 2 | 1 | 3 | 1 | 4 | 4 | 3 | 3 | 2 | 6 | 2 | 1 | 4 | 2 | 2 | 4 | 6 |
| 454 | 2 | 2 | 2 | 2 | 2 | 1 | 4 | 4 | 4 | 4 | 4 | 4 | 3 | 5 | 5 | 4 | 5 | 5 | 5 | 4 | 6 | 5 | 1 | 2 | 2 | 2 | 2 | 2 |
| 455 | 2 | 2 | 2 | 2 | 1 | 1 | 1 | 1 | 6 | 1 | 1 | 1 | 1 | 1 | 1 | 4 | 4 | 3 | 4 | 2 |  | 1 | 1 | 9 | 6 | 6 | 6 | 4 |
| 464 | 2 | 1 | 1 | 2 | 1 | 1 | 2 | 2 | 2 | 2 | 3 | 2 | 1 | 2 | 2 | 4 | 4 | 3 | 4 | 2 |  | 2 | 1 | 4 | 4 | 4 | 2 | 4 |
| 466 | 2 | 2 | 2 | 2 | 1 | 1 | 2 | 4 | 4 | 4 | 3 | 4 | 3 | 3 | 4 | 4 | 4 | 3 | 4 | 3 | 4 | 3 | 1 | 2 | 1 | 3 | 4 | 5 |
| 469 | 2 | 1 | 2 | 2 | 1 | 1 | 2 | 4 | 2 | 1 | 3 | 3 | 3 | 3 | 3 | 4 | 4 | 4 | 3 | 2 | 4 | 3 | 1 | 6 | 7 | 5 | 5 | 7 |
| 471 | 2 | 2 | 2 | 2 | 1 | 1 | 2 | 3 | 3 | 3 | 3 | 3 | 2 | 2 | 2 | 5 | 5 | 2 | 5 | 2 | 5 | 3 | 1 | 9 | 2 | 4 | 4 | 4 |
| 472 | 2 | 2 | 2 | 2 | 1 | 1 | 4 | 4 | 4 | 4 | 4 | 4 | 2 | 3 | 5 | 5 | 5 | 5 | 5 | 4 | 6 | 4 | 1 | 7 | 7 | 8 | 7 | 0 |
| 481 | 1 | 1 | 1 | 1 | 1 | 1 | 2 | 3 | 2 | 2 | 3 | 4 | 1 | 2 | 1 | 4 | 4 | 1 | 1 | 2 | 1 | 2 | 1 | 6 | 3 | 6 | 6 | 2 |
| 483 | 1 | 1 | 2 | 2 | 1 | 1 | 2 | 3 | 3 | 3 | 4 | 4 | 3 | 3 | 3 | 2 | 3 | 2 | 3 | 3 | 6 | 3 | 1 | 4 | 4 | 4 | 4 | 3 |
| 487 | 1 | 1 | 2 | 2 | 1 | 1 | 3 | 2 | 3 |  |  |  |  |  | 3 | 4 | 2 | 4 | 4 | 3 | 6 | 3 | 1 | 5 | 6 | 3 | 0 | 0 |
| 491 | 1 | 1 | 2 | 2 | 1 | 1 | 1 | 3 | 1 | 1 | 2 | 3 | 1 | 2 | 1 | 2 | 2 | 1 | 1 | 2 | 3 | 2 | 1 | 9 | 6 | 6 | 6 | 4 |
| 493 | 2 | 2 | 2 | 2 | 2 | 3 |  |  |  |  |  |  |  |  |  |  |  |  |  |  |  |  | 3 |  |  |  |  | 2 |
| 498 | 2 | 1 | 2 | 2 | 1 | 1 | 1 | 2 | 2 | 2 | 4 | 4 | 3 | 2 | 2 | 3 | 4 | 3 | 3 | 2 | 3 | 2 | 1 | 6 | 7 | 7 | 7 | 2 |
| 499 | 2 | 1 | 1 | 2 | 1 | 1 | 2 | 4 | 2 | 2 | 4 | 3 | 2 | 2 | 2 | 2 | 2 | 4 | 2 | 2 | 4 | 2 | 1 | 7 | 7 | 2 | 7 | 7 |
| 504 | 2 | 2 | 2 | 2 | 1 | 1 | 3 | 3 | 3 | 2 | 3 | 3 | 2 | 2 | 3 | 3 | 3 | 4 | 3 | 2 | 3 | 2 | 1 | 9 | 5 | 7 | 3 | 6 |
| 506 | 2 | 2 | 2 | 2 | 2 | 1 | 2 | 4 | 2 | 3 | 2 |  | 2 | 2 | 1 | 5 | 5 | 4 | 5 | 2 | 6 | 1 | 1 | 9 | 2 | 6 | 6 | 0 |
| 512 | 1 | 1 | 1 | 1 | 1 | 1 | 1 | 2 | 1 | 1 | 4 | 4 | 1 | 2 | 1 | 4 | 4 | 3 | 4 | 3 | 6 | 4 | 1 | 8 | 8 | 8 | 8 | 1 |
| 513 | 1 | 1 | 1 | 1 | 1 | 3 |  |  |  |  |  |  |  |  |  |  |  |  |  |  |  |  | 3 |  |  |  |  |  |
| 514 | 2 | 1 | 2 | 2 | 1 | 1 | 2 | 4 | 3 | 5 | 5 | 5 | 2 | 3 | 5 | 4 | 4 | 4 | 4 | 5 | 4 | 4 | 1 | 4 | 4 | 0 | 4 | 0 |
| 515 | 1 | 1 | 1 | 1 | 1 | 1 | 1 | 3 | 1 | 1 | 1 | 1 | 1 | 1 | 1 | 4 | 4 | 3 | 3 | 1 | 1 | 1 | 1 | 8 | 8 | 8 | 6 | 4 |
| 518 | 2 | 2 | 2 | 2 | 1 |  | 2 | 3 | 3 | 2 | 3 | 5 | 4 | 2 | 3 | 5 | 5 | 3 | 4 | 2 | 6 | 3 | 1 | 9 | 5 | 5 | 7 | 1 |
| 519 | 2 | 2 | 2 | 2 | 1 | 1 | 3 | 4 | 4 | 4 | 4 | 4 | 4 | 2 |  | 5 | 5 | 4 | 5 | 2 | 3 | 4 | 1 | 9 | 5 | 6 | 5 | 1 |
| 520 | 2 | 2 | 2 | 2 | 1 | 1 | 1 | 3 | 3 | 3 | 3 | 3 | 3 | 2 | 1 | 3 | 3 | 3 | 3 | 3 | 3 | 3 | 1 | 4 | 4 | 4 | 4 | 4 |
| 525 | 2 | 2 | 2 | 2 | 1 | 1 | 3 | 3 | 4 | 3 | 2 | 5 | 1 | 4 | 5 | 3 | 3 | 5 | 4 | 4 | 3 | 4 | 1 |  |  |  | 2 | 1 |
| 527 | 2 | 2 | 2 | 2 | 2 | 1 |  |  |  |  | 5 | 5 | 4 | 4 |  | 4 |  | 4 | 3 |  | 6 | 3 | 1 | 0 |  | 3 | 4 | 2 |
| 528 | 1 | 1 | 1 | 1 | 1 | 1 | 1 | 3 | 1 | 1 | 2 | 4 | 1 | 1 | 1 | 3 | 1 | 2 | 3 | 1 | 1 | 1 | 1 | 8 | 8 | 8 | 6 | 4 |
| 529 | 2 | 1 | 1 | 2 | 1 | 1 | 2 | 3 | 2 | 2 | 3 | 5 | 2 | 3 | 6 | 3 | 4 | 2 | 2 | 2 | 4 | 3 | 1 | 6 | 6 | 6 | 8 | 0 |
| 531 | 2 | 2 | 2 | 2 | 2 | 1 | 2 | 4 | 4 | 4 | 4 | 5 | 3 | 3 | 3 | 6 | 4 | 4 | 4 | 3 | 6 | 3 | 1 | 3 | 2 | 1 | 5 | 2 |
| 538 | 2 | 2 | 2 | 2 | 2 | 1 | 5 | 4 | 4 | 4 | 4 | 4 | 4 | 4 |  | 5 | 5 | 5 | 5 | 5 |  | 5 | 1 | 9 | 0 | 4 | 0 | 4 |
| 542 | 2 | 2 | 1 | 2 | 1 | 1 | 3 | 4 | 4 | 4 | 4 | 4 | 3 | 3 | 1 | 4 | 4 | 3 | 3 | 3 | 4 | 4 | 1 | 2 | 2 | 2 | 2 | 0 |
| 546 | 2 | 2 | 2 | 2 | 1 | 1 | 3 | 3 | 3 | 3 | 4 | 4 | 4 | 3 | 2 | 4 | 4 | 4 | 4 | 3 | 4 | 4 | 1 | 9 | 4 | 1 | 1 | 1 |
| 547 | 2 | 2 | 2 | 2 | 1 | 1 | 3 | 3 | 3 | 3 | 3 | 3 | 3 | 4 | 2 | 5 | 5 | 5 | 4 | 3 |  | 3 | 1 |  | 2 | 2 | 4 | 4 |
| 549 | 2 | 1 | 2 | 2 | 1 | 1 | 2 | 2 | 3 | 2 | 5 | 5 | 2 | 2 | 3 | 4 | 5 | 4 | 4 | 3 | 3 | 4 | 1 | 4 | 6 | 6 | 4 | 2 |
| 559 | 2 | 2 | 2 | 2 | 2 | 1 | 2 | 2 | 3 | 3 | 3 | 1 | 3 | 4 | 3 | 5 | 5 | 2 | 4 | 2 | 6 | 3 | 1 | 2 | 0 | 4 | 4 | 6 |
| 561 | 2 | 2 | 2 | 2 | 2 | 1 | 3 | 4 |  | 2 | 3 |  | 3 | 3 |  | 5 | 5 | 3 | 3 | 3 |  | 3 | 1 | 9 | 2 | 2 | 6 |  |
| 569 | 2 | 1 | 2 | 2 | 2 | 1 | 5 | 5 | 5 | 5 | 5 | 5 | 5 | 5 | 5 | 5 | 5 | 5 | 5 | 5 | 5 | 5 | 1 | 0 | 0 | 0 | 0 | 0 |
| 575 | 2 | 2 | 2 | 2 | 2 | 1 | 4 | 4 | 4 | 4 | 4 | 5 | 4 | 5 | 4 | 5 | 5 | 5 | 4 | 5 | 5 | 5 | 1 | 0 | 0 | 0 | 4 | 0 |
| 595 | 2 | 2 | 2 | 2 | 2 | 1 | 5 | 5 | 5 | 5 | 5 | 5 | 5 | 5 | 5 | 5 | 5 | 5 | 5 | 5 | 5 | 5 | 1 | 0 | 0 | 0 | 4 | 0 |
| 596 | 2 | 2 | 2 | 2 | 1 | 1 | 3 | 3 | 4 | 4 | 4 | 4 | 4 | 3 | 3 | 4 | 4 | 4 | 4 | 4 | 4 | 4 | 1 | 9 | 2 | 5 | 3 | 1 |
| 607 | 2 | 2 | 2 | 2 | 2 | 1 | 3 | 3 | 3 | 3 | 3 | 3 | 3 | 4 | 3 | 3 | 4 | 4 | 4 | 4 | 4 | 3 | 2 | 9 |  |  |  |  |
| 611 | 2 | 2 | 2 | 2 | 2 | 1 | 2 | 3 | 6 | 2 |  | 4 | 2 | 1 | 6 | 5 | 5 | 1 | 1 | 3 | 4 | 2 | 1 | 9 | 8 | 8 | 8 | 0 |
| 614 | 2 | 2 | 2 | 2 | 2 | 1 | 2 | 4 | 3 | 3 | 4 | 5 | 3 | 3 | 3 | 5 | 5 | 4 | 5 | 3 | 3 | 3 | 1 | 2 | 2 | 2 | 1 | 0 |
| 622 | 2 | 2 | 2 | 2 | 2 | 1 | 3 | 5 | 5 | 5 | 5 | 5 | 5 | 5 | 5 | 5 | 5 | 5 | 5 | 4 | 3 | 4 | 1 | 1 | 0 | 2 | 0 | 0 |
| 629 | 1 | 2 | 2 | 2 | 1 | 1 | 2 | 2 | 1 | 1 | 2 | 3 | 1 | 2 | 1 |  | 4 | 2 | 2 |  | 6 | 3 | 1 | 8 | 8 | 8 | 8 | 8 |
| 632 | 2 | 2 | 2 | 2 | 2 | 1 | 3 | 4 | 3 | 4 | 4 | 4 | 4 | 4 | 4 | 4 | 4 | 4 | 4 | 4 | 4 | 4 | 1 | 0 | 0 | 0 | 0 | 0 |
| 638 | 2 | 2 | 2 | 2 | 2 | 1 | 3 | 4 | 4 | 4 | 4 | 4 | 4 | 4 | 2 | 5 | 4 | 4 | 4 | 5 | 4 | 5 | 1 | 3 | 2 | 3 | 1 | 3 |
| 640 | 1 | 1 | 1 | 2 | 1 | 1 | 2 | 3 | 1 | 2 | 3 | 3 | 2 | 2 | 2 | 1 | 1 | 2 | 2 | 2 | 3 | 2 | 1 | 8 | 6 | 6 | 4 | 4 |
| 641 | 2 | 2 | 2 | 2 | 2 | 1 | 4 | 5 | 5 | 5 | 5 | 5 | 4 | 4 | 5 | 4 |  | 5 | 5 | 5 | 6 | 5 | 1 | 2 | 0 | 1 | 0 | 0 |
| 644 | 2 | 2 | 2 | 2 | 2 | 1 | 3 | 5 | 5 | 5 | 5 | 5 | 4 | 5 | 4 | 4 | 5 | 5 | 4 | 5 | 3 | 4 | 1 | 0 | 0 | 0 | 0 | 0 |
| 649 | 1 | 1 | 2 | 2 | 1 | 1 | 2 | 2 | 2 | 2 | 2 | 2 | 2 | 2 | 1 | 4 | 4 | 3 | 3 | 3 | 2 | 2 | 1 | 9 | 4 | 8 | 6 | 4 |
| 650 | 1 | 1 | 1 | 2 | 1 | 1 | 1 | 2 | 1 | 2 | 2 | 4 | 2 | 2 | 4 |  | 4 | 4 | 4 | 3 | 3 | 2 | 1 | 8 | 3 | 6 | 5 | 2 |
| 651 | 2 | 2 | 2 | 2 | 2 | 1 | 2 | 2 | 1 | 1 | 1 | 1 | 1 | 1 | 1 | 1 | 1 | 1 | 2 | 2 |  | 1 | 1 | 9 | 7 | 8 | 6 | 6 |
| 652 | 2 | 2 | 2 | 2 | 2 | 1 | 3 | 4 | 3 | 3 | 3 | 4 | 3 | 4 | 1 | 4 | 4 | 1 | 4 | 3 | 2 | 3 | 1 | 4 | 4 | 4 | 4 | 2 |
| 653 | 2 | 2 | 2 | 2 | 2 | 1 | 5 | 5 | 5 | 5 | 5 | 5 | 5 | 5 |  | 5 | 5 | 5 | 5 | 5 | 5 | 5 | 1 | 0 | 0 | 0 | 0 | 0 |
| 654 | 2 | 2 | 2 | 2 | 1 | 1 | 4 | 4 | 4 | 4 | 4 | 4 | 4 | 4 | 4 | 5 | 5 | 4 | 4 | 4 | 4 | 4 | 1 | 8 | 8 | 8 | 8 | 8 |
| 656 | 2 | 1 | 2 | 2 | 1 | 1 | 2 | 2 | 3 | 2 | 5 | 5 | 2 | 2 | 3 | 4 | 5 | 4 | 4 | 3 | 3 | 4 | 1 | 4 | 6 | 6 | 4 | 2 |
| 662 | 1 | 1 | 2 | 2 | 1 | 1 | 1 | 2 | 1 | 1 | 3 | 3 | 1 | 2 | 1 | 4 | 4 | 3 | 2 | 2 |  | 2 | 1 | 8 | 7 | 4 | 4 | 4 |
| 663 | 1 | 1 | 1 | 1 | 1 | 1 | 2 | 2 | 1 | 2 | 2 | 4 | 3 | 2 | 1 | 2 | 5 | 3 | 2 | 2 |  | 2 | 1 | 8 | 2 | 0 | 2 | 8 |
| 665 | 2 | 2 | 2 | 2 | 2 | 1 | 3 | 3 | 4 | 2 | 4 | 4 | 4 | 4 | 4 | 4 | 4 | 5 | 4 | 4 | 4 | 4 | 1 | 2 | 0 | 0 | 5 | 0 |
| 670 | 1 | 1 | 1 | 2 | 1 | 1 | 2 | 3 | 2 | 2 | 4 | 4 | 3 | 2 | 2 | 4 | 2 | 3 | 3 | 2 |  | 2 | 1 | 7 | 6 | 4 | 5 | 3 |
| 672 | 2 | 2 | 2 | 2 | 1 | 1 | 3 | 3 | 3 | 3 | 3 | 3 | 2 | 3 | 2 | 3 | 4 | 4 | 4 | 3 | 4 | 3 | 1 | 9 | 2 | 5 | 4 | 4 |
| 674 | 2 | 1 | 2 | 2 | 1 | 1 | 3 | 3 | 3 | 1 | 2 | 4 | 1 | 1 | 1 | 5 | 5 | 1 | 4 | 2 | 3 | 1 | 1 | 6 | 5 | 8 | 7 | 2 |
| 677 | 1 | 1 | 1 | 1 | 1 | 1 | 2 | 3 | 6 | 2 | 3 | 4 | 3 | 3 | 3 | 3 | 3 | 3 | 3 | 3 | 3 | 2 | 1 | 9 | 5 | 4 | 4 | 0 |
| 678 | 1 | 1 | 1 | 1 | 1 | 1 | 2 | 3 | 2 | 2 | 3 | 3 | 2 | 3 | 1 | 4 | 4 | 3 | 2 | 3 | 1 | 3 | 1 | 7 | 6 | 7 | 5 | 3 |
| 685 | 1 | 1 | 1 | 1 | 1 | 1 | 1 | 4 | 1 | 1 | 2 | 5 | 1 | 1 |  | 3 | 5 | 2 | 6 | 2 | 6 | 2 | 1 | 8 | 7 | 8 | 7 | 3 |
| 686 | 2 | 1 | 2 | 2 | 1 | 1 | 3 | 3 | 4 | 4 | 4 | 4 | 4 | 4 | 5 | 3 | 5 | 5 | 5 | 4 | 6 | 3 | 1 | 3 | 1 | 1 | 0 | 2 |
| 689 | 2 | 2 | 2 | 2 | 2 | 1 | 2 | 4 | 2 | 2 | 4 | 4 | 2 | 2 | 2 | 4 | 4 | 1 | 4 | 4 |  | 4 | 1 | 8 | 8 | 8 |  | 6 |
| 690 | 2 | 2 | 2 | 2 | 2 | 1 | 3 | 3 |  | 3 | 4 | 4 | 3 | 4 |  | 2 | 4 | 3 | 2 | 3 | 4 | 4 | 1 | 9 | 4 | 4 | 4 | 2 |
| 693 | 1 | 1 | 2 | 2 | 1 | 1 | 2 | 3 | 2 | 2 | 3 | 3 | 3 | 3 | 3 | 2 | 4 | 4 | 3 | 4 | 3 | 3 | 1 | 0 | 2 | 2 | 3 | 8 |
| 695 | 2 | 2 | 2 | 2 | 2 | 1 | 4 | 5 | 4 | 4 | 4 | 4 | 4 | 4 | 2 | 5 | 5 | 5 | 5 | 4 | 4 | 4 | 3 |  |  |  |  |  |
| 697 | 1 | 1 | 1 | 2 | 1 | 1 | 1 | 2 | 6 | 1 | 2 | 2 | 1 | 1 | 1 |  | 1 | 1 | 5 | 2 | 2 | 2 | 1 | 9 | 8 | 8 | 7 | 6 |
| 699 | 2 | 2 | 2 | 2 | 2 | 1 | 3 | 3 | 4 | 2 | 4 | 4 | 2 | 4 | 2 | 4 | 4 | 4 | 4 | 4 | 4 | 4 | 1 | 3 | 6 | 6 | 5 | 1 |
| 701 | 2 | 2 | 2 | 2 | 2 | 1 | 2 | 3 | 2 | 2 | 4 | 5 | 2 | 2 | 2 | 4 | 5 | 2 | 4 | 2 | 5 | 2 | 1 | 6 | 7 | 7 | 7 | 0 |
| 702 | 1 | 1 | 1 | 1 | 1 | 1 | 2 | 3 | 1 | 2 | 3 | 4 | 3 | 3 | 2 | 4 | 4 | 3 | 2 | 4 | 2 | 3 | 1 | 8 | 4 | 4 | 6 | 2 |
| 703 | 2 | 2 | 2 | 2 | 2 | 1 | 5 | 5 | 5 | 5 | 5 | 5 | 3 |  | 5 | 5 | 5 | 5 | 5 | 5 | 3 | 4 | 1 | 0 | 3 | 0 | 0 | 0 |
| 705 | 2 | 2 | 2 | 2 | 2 | 1 | 4 | 4 | 4 | 4 | 4 | 4 | 4 | 4 | 3 | 4 | 5 | 5 | 5 | 4 | 4 | 4 | 1 | 9 | 0 | 0 | 0 | 0 |
| 707 | 2 | 2 | 2 | 2 | 2 | 1 |  | 5 | 6 | 2 | 3 | 5 | 2 | 2 | 6 | 4 | 5 | 4 | 4 | 2 | 4 | 3 | 1 | 0 | 5 | 4 | 4 | 1 |
| 708 | 1 | 1 | 1 | 1 | 1 | 1 | 4 | 1 | 6 | 1 | 1 | 1 | 1 | 1 |  | 1 | 5 | 4 | 1 | 1 |  | 1 | 1 | 8 | 8 | 8 | 8 | 8 |
| 709 | 2 | 1 | 2 | 2 | 1 | 1 | 1 | 1 | 1 | 1 | 1 | 2 | 1 | 2 | 1 | 3 | 3 | 2 | 3 | 1 | 3 | 1 | 1 |  | 8 | 8 | 6 | 4 |
| 711 | 2 | 1 | 2 | 2 | 2 | 1 | 2 | 3 | 2 | 2 | 3 | 4 | 2 | 3 | 4 | 4 | 4 | 4 | 4 | 3 | 6 | 3 | 1 | 9 | 4 | 6 | 4 | 2 |
| 714 | 2 | 2 | 2 | 2 | 2 | 1 | 1 | 1 | 6 | 1 | 3 | 4 | 2 | 1 | 1 | 4 | 4 | 1 | 1 | 1 | 3 | 1 | 1 | 9 | 0 | 0 | 4 | 2 |
| 721 | 1 | 1 | 2 | 2 | 1 | 1 | 2 | 4 | 1 | 1 | 4 | 4 | 1 | 1 | 1 | 4 | 4 | 4 | 4 | 4 | 3 | 3 | 1 | 8 | 6 | 8 | 8 | 6 |
| 723 | 2 | 2 | 2 | 2 | 2 | 1 | 2 | 3 | 2 | 2 | 3 | 5 | 1 | 1 | 2 | 4 | 4 | 2 | 4 | 3 | 3 | 3 | 1 | 9 | 2 | 4 | 6 | 2 |
| 725 | 1 | 1 | 2 | 2 | 1 | 1 | 1 | 2 | 1 | 1 | 1 | 4 | 1 | 1 | 1 | 3 | 4 | 2 | 1 | 2 | 6 | 1 | 1 | 8 | 8 | 8 | 8 | 2 |
| 727 | 2 | 2 | 2 | 2 | 1 | 1 | 5 | 4 | 5 | 3 | 4 | 4 | 5 | 4 | 1 | 5 | 5 | 5 | 4 | 4 | 4 | 4 | 1 | 3 | 1 | 1 | 1 | 0 |
| 729 | 2 | 2 | 2 | 2 | 2 | 1 | 1 | 1 |  | 1 | 5 | 5 | 1 | 1 | 1 | 4 | 4 | 1 | 2 | 1 | 1 | 1 |  | 8 | 8 | 8 | 8 | 3 |
| 730 | 1 | 1 | 1 | 2 | 1 | 1 | 3 | 5 | 1 | 2 | 4 | 5 | 2 | 2 | 3 | 4 | 5 | 3 | 5 | 3 | 5 | 3 | 1 | 8 | 4 | 4 | 4 | 0 |
| 733 | 2 | 2 | 2 | 2 | 1 | 1 | 1 | 3 | 3 | 1 | 3 | 4 | 2 | 4 | 1 | 3 | 5 | 2 | 4 | 4 | 4 | 4 | 1 | 0 | 0 | 4 | 4 | 0 |
| 736 | 1 | 1 | 1 | 2 | 1 | 1 | 1 | 3 | 1 | 1 | 4 | 5 | 1 | 2 | 3 | 1 | 3 | 2 | 4 | 3 | 4 | 2 | 1 | 8 | 6 | 8 | 7 | 2 |
| 738 | 1 | 1 | 1 | 2 | 1 | 1 | 1 | 3 |  | 2 | 3 | 3 | 1 | 1 | 6 | 1 | 4 | 1 | 2 | 1 | 2 | 2 | 1 | 8 | 7 | 8 | 7 | 4 |
| 739 | 2 | 1 | 2 | 2 | 1 | 1 | 1 | 3 | 4 | 2 | 3 | 4 | 2 | 2 | 3 | 5 | 4 | 4 | 3 | 2 | 2 | 2 | 1 | 3 | 6 | 7 | 6 | 3 |
| 741 | 2 | 2 | 2 | 2 | 1 | 1 | 1 | 3 | 4 | 4 | 5 | 4 | 2 | 3 | 3 | 5 | 5 | 5 | 2 | 2 | 2 | 2 | 1 | 1 | 1 | 3 | 5 | 2 |
| 742 | 2 | 2 | 2 | 2 | 2 | 1 | 4 | 3 | 3 | 4 | 4 | 4 | 5 | 4 | 4 | 5 | 5 | 5 | 3 | 3 | 6 | 5 | 1 | 1 | 3 | 2 | 2 | 0 |
| 743 | 1 | 5 | 5 | 5 | 1 | 1 | 1 | 3 | 1 | 1 | 1 | 4 | 1 | 1 | 1 | 2 | 3 | 1 | 3 | 1 | 1 | 1 | 1 | 8 | 8 | 8 | 8 | 4 |
| 747 | 2 | 2 | 2 | 2 | 2 | 1 | 2 | 3 | 1 | 1 | 2 | 3 | 1 | 1 | 1 | 4 | 4 | 2 | 3 | 2 | 2 | 2 | 1 | 9 | 8 | 7 | 7 | 4 |
| 748 | 2 | 2 | 2 | 2 | 1 | 1 | 4 | 5 | 5 | 5 | 5 | 5 | 5 | 5 | 3 | 5 | 5 | 5 | 3 | 5 | 5 | 5 | 1 | 0 | 0 | 0 | 0 | 0 |
| 751 | 1 | 1 | 1 | 2 | 1 | 1 | 2 | 2 |  | 2 |  | 3 | 3 | 3 | 4 | 3 | 4 | 5 | 5 | 3 | 3 | 3 | 1 | 6 | 4 | 6 | 4 | 2 |
| 767 | 2 | 2 | 2 | 2 | 1 | 1 | 1 | 2 | 2 | 2 | 2 | 4 | 3 | 3 | 3 | 4 | 5 | 3 | 3 | 3 |  | 3 | 1 |  | 4 | 4 | 4 | 4 |
| 769 | 2 | 1 | 2 | 2 | 2 | 1 | 2 | 1 | 3 | 3 | 1 | 3 | 3 | 3 | 2 | 3 | 4 | 4 | 4 | 3 | 3 | 2 | 1 | 3 | 2 | 4 | 2 | 3 |
| 770 | 1 | 1 | 1 | 2 | 1 | 1 | 2 | 2 |  | 2 | 1 | 2 | 4 | 2 | 2 | 3 | 3 | 4 | 2 | 2 | 1 | 1 | 1 | 6 | 7 | 8 | 4 | 4 |
| 771 | 1 | 1 | 1 | 2 | 1 | 1 | 1 | 3 | 5 | 1 | 1 | 1 | 1 | 1 | 1 | 1 | 1 | 1 | 1 | 2 | 6 | 1 | 2 |  |  |  |  |  |
| 782 | 2 | 2 | 2 | 2 | 2 | 2 |  |  |  |  |  |  |  |  |  |  |  |  |  |  |  |  | 1 | 1 | 1 | 4 | 4 | 2 |
| 785 | 2 | 2 | 2 | 2 | 2 | 1 | 4 | 4 | 4 | 3 | 5 | 5 | 5 | 4 | 2 | 4 | 4 | 4 | 4 | 4 | 4 | 4 | 1 | 0 | 1 | 0 | 0 | 0 |
| 789 | 2 | 2 | 2 | 2 | 1 | 1 | 1 | 2 | 1 | 1 | 1 | 1 | 1 | 1 | 1 |  |  | 5 | 5 | 1 | 3 | 1 | 1 | 8 | 8 | 8 | 8 | 8 |
| 790 | 2 | 1 | 2 | 2 | 1 | 1 | 2 | 3 | 3 | 2 | 3 | 3 | 2 | 3 | 2 | 3 | 4 | 3 | 4 | 2 | 4 | 2 | 1 | 4 | 6 | 7 | 6 | 0 |
| 791 | 2 | 2 | 1 | 1 | 1 | 1 | 2 | 3 |  | 1 | 4 | 4 | 2 | 2 | 3 | 3 | 4 | 4 | 3 | 3 | 3 | 3 | 1 | 8 | 6 | 4 | 6 | 1 |
| 792 | 2 | 2 | 2 | 2 | 1 | 1 | 2 | 4 | 4 | 4 | 4 | 4 | 3 | 4 | 3 | 5 | 5 | 5 | 4 | 3 | 2 | 4 | 1 | 3 | 4 | 4 | 3 | 3 |
| 793 | 2 | 2 | 2 | 2 | 1 | 1 | 2 | 3 | 2 | 2 | 4 | 4 | 1 | 3 | 6 | 4 | 4 | 2 | 2 | 2 | 2 | 3 | 1 | 9 | 4 | 6 | 6 | 2 |
| 794 | 2 | 1 | 2 | 2 | 1 | 2 |  |  |  |  |  |  |  |  |  |  |  |  |  |  |  |  | 2 |  |  |  |  |  |
| 796 | 2 | 2 | 2 | 2 | 1 | 1 | 3 | 3 | 3 | 2 | 3 | 3 |  | 3 | 2 | 4 | 4 | 3 | 4 |  | 4 | 3 | 3 |  |  |  |  |  |
| 803 | 2 | 2 | 2 | 2 | 1 | 1 | 4 | 4 | 4 | 4 | 4 | 4 | 4 | 4 |  | 5 | 5 | 5 | 5 | 5 |  | 4 | 1 | 0 | 0 | 0 | 0 | 0 |
| 809 | 2 | 2 | 2 | 2 | 1 | 1 | 2 | 3 |  | 6 | 6 | 2 | 2 | 1 | 1 |  | 5 | 2 | 2 | 2 | 5 | 3 | 1 | 9 | 8 |  | 8 | 8 |
| 812 | 2 | 2 | 2 | 2 | 2 | 1 | 4 | 4 | 3 | 3 | 3 | 4 | 3 | 4 | 3 |  | 4 | 4 | 4 | 4 | 4 | 4 | 1 | 9 | 3 | 2 | 2 | 2 |
| 813 | 2 | 2 | 2 | 2 | 2 | 1 | 4 | 4 | 6 | 4 | 4 | 5 | 5 | 4 | 3 | 4 | 5 | 5 | 4 | 4 | 4 | 4 | 1 | 2 | 0 | 2 | 2 | 3 |
| 815 | 1 | 1 | 1 | 1 | 1 | 1 | 2 | 3 | 6 | 3 | 4 | 5 | 3 | 3 | 3 | 4 | 5 | 2 | 3 | 3 | 6 | 3 | 1 | 6 | 5 | 5 | 5 | 2 |
| 816 | 2 | 2 | 2 | 2 | 1 | 1 | 1 | 1 |  | 3 | 2 | 5 | 1 | 1 | 3 | 4 | 4 | 2 | 1 | 1 |  | 1 | 1 | 0 | 6 | 8 | 8 | 2 |
| 825 | 1 | 1 | 1 | 1 | 1 | 1 | 3 | 3 | 6 | 3 | 2 | 3 | 2 | 3 | 2 | 4 | 4 | 3 | 4 | 3 | 1 | 3 | 1 | 8 | 5 | 6 | 4 | 4 |
| 830 | 2 | 2 | 2 | 2 | 2 | 1 | 3 | 3 | 2 | 3 | 3 | 3 | 3 | 4 | 3 | 3 | 4 | 5 | 4 | 4 | 4 | 4 | 1 | 0 | 0 | 0 | 0 | 0 |
| 838 | 2 | 2 | 2 | 2 | 2 | 1 | 2 | 3 | 2 | 2 | 5 | 5 | 2 | 2 | 2 | 5 | 5 | 2 | 5 | 3 | 1 | 2 | 1 | 4 | 4 | 3 | 2 | 7 |
| 842 | 2 | 2 | 2 | 2 | 1 | 1 | 2 | 3 | 3 | 3 | 3 | 4 | 2 | 2 | 3 | 5 | 3 | 1 | 2 | 3 | 6 | 3 | 1 | 9 | 4 | 4 | 6 | 2 |
| 847 | 1 | 1 | 1 | 2 | 1 | 1 | 1 | 2 | 1 | 1 | 1 | 5 | 1 | 1 | 1 | 1 | 4 | 1 | 4 | 1 | 3 | 1 | 1 | 8 | 8 | 8 | 8 | 4 |
| 856 | 1 | 1 | 1 | 2 | 1 | 1 | 1 | 3 | 1 | 1 | 2 | 2 | 2 | 2 | 6 | 2 | 2 | 2 | 2 | 3 |  | 3 | 1 | 8 | 6 | 6 | 6 | 5 |
| 858 | 1 | 1 | 1 | 2 | 1 | 1 | 1 | 1 | 1 | 1 | 1 | 5 | 1 | 1 | 4 | 4 | 3 | 1 | 1 | 1 | 1 | 1 | 1 | 8 | 7 | 8 | 7 | 0 |
| 863 | 2 | 2 | 2 | 2 | 1 | 1 | 2 | 3 | 4 | 4 | 4 | 5 | 2 | 2 | 4 | 4 | 4 | 4 | 4 | 3 | 4 | 3 | 1 | 6 | 3 | 7 | 6 | 5 |
| 870 | 1 | 1 | 2 | 2 | 1 | 1 | 1 | 3 | 1 | 4 | 4 | 5 | 1 | 1 | 3 | 3 | 5 | 4 | 4 | 2 | 2 | 2 | 1 | 8 | 6 | 6 | 6 | 4 |
| 871 | 2 | 1 | 2 | 2 | 1 | 1 | 2 | 2 | 2 | 2 | 2 | 4 | 2 | 1 | 1 | 2 | 4 | 2 | 5 | 2 | 3 | 2 | 1 | 4 | 6 | 6 | 2 | 1 |
| 874 | 2 | 1 | 2 | 2 | 1 | 1 | 2 | 5 | 2 | 2 | 5 | 5 | 5 | 3 | 3 | 5 | 5 | 2 | 2 | 5 | 2 | 5 | 1 | 6 | 4 | 4 | 4 | 0 |
| 880 | 2 | 2 | 2 | 2 | 1 | 1 | 3 |  | 6 | 3 | 3 | 4 | 3 | 3 | 2 | 4 | 3 | 3 | 3 |  | 3 | 3 | 1 | 4 | 4 | 4 | 4 | 4 |
| 882 | 1 | 2 | 1 | 2 | 1 | 1 | 1 | 1 | 1 | 3 | 2 | 2 | 3 | 4 | 1 | 1 | 4 | 3 | 4 | 2 | 6 | 2 | 1 | 8 | 2 | 6 | 4 | 4 |
| 890 | 1 | 1 | 1 | 2 | 1 | 1 | 2 | 3 | 2 | 2 | 2 | 3 | 3 | 2 | 1 | 4 | 5 | 2 | 2 | 2 | 2 | 3 | 1 | 6 | 6 | 8 | 6 | 4 |
| 899 | 2 | 2 | 2 | 2 | 2 | 1 | 3 | 3 | 3 | 3 | 3 | 3 | 3 | 3 | 3 | 3 | 5 | 4 | 4 | 4 | 4 | 4 | 1 | 0 | 0 | 0 | 0 | 0 |
| 903 | 2 | 2 | 2 | 2 | 1 | 1 | 3 | 3 | 4 | 4 | 4 | 4 | 4 | 4 | 4 | 4 | 4 | 5 | 4 | 3 | 3 | 3 | 1 | 2 | 2 | 4 | 4 | 2 |
| 911 | 2 | 2 | 2 | 2 | 2 | 1 | 1 | 4 |  | 1 | 5 | 4 | 1 | 1 | 6 | 4 |  | 1 | 1 | 5 | 4 | 2 | 1 | 8 | 8 | 8 | 8 | 2 |
| 914 | 2 | 1 | 2 | 2 | 2 | 1 | 2 | 4 | 2 | 2 | 3 | 3 | 2 | 2 | 1 | 4 | 4 | 2 | 2 | 4 | 4 | 2 | 1 | 7 | 7 | 8 | 7 | 6 |
| 920 | 1 | 1 | 2 | 2 | 1 | 1 | 2 | 3 | 1 | 3 | 1 | 3 | 1 | 1 | 1 | 2 | 4 | 4 | 4 | 1 | 1 | 1 | 1 | 8 | 2 | 8 | 1 | 8 |
| 929 | 2 | 2 | 2 | 2 | 1 | 1 | 2 | 2 | 4 | 4 | 4 | 5 | 4 | 4 |  |  | 5 | 3 | 4 | 3 |  | 3 | 1 | 9 | 2 | 4 | 2 | 2 |
| 935 | 2 | 2 | 2 | 2 | 1 | 1 | 1 | 2 |  | 1 | 1 | 2 | 1 | 1 | 1 | 3 | 4 | 1 | 1 | 1 |  | 2 | 1 | 0 | 0 | 0 | 0 | 2 |
| 954 | 1 | 1 | 1 | 1 | 1 | 1 | 3 | 4 | 6 | 1 | 4 | 4 | 1 | 2 | 2 | 3 | 4 | 3 | 4 | 4 | 3 | 4 | 1 | 4 | 4 | 6 | 0 | 0 |
| 959 | 2 | 2 | 2 | 2 | 1 | 1 | 4 | 4 | 6 | 3 |  | 4 | 3 | 3 |  | 3 | 3 | 3 | 2 | 3 | 3 | 3 | 1 | 5 | 1 | 0 | 3 | 6 |
| 968 | 2 | 2 | 2 | 2 | 1 | 1 | 3 | 4 | 6 | 3 | 3 | 4 | 3 | 3 | 1 | 5 | 5 | 4 | 4 | 3 | 3 | 3 |  | 9 | 5 | 4 | 2 | 8 |
| 972 | 2 | 2 | 2 | 2 | 2 | 1 | 3 | 3 | 3 | 3 | 3 | 4 | 3 |  | 3 |  |  |  |  |  |  | 4 | 1 | 6 | 4 | 2 | 2 | 2 |
| 977 | 2 | 2 | 2 | 2 | 1 | 1 | 2 | 2 | 6 | 3 | 2 | 3 | 2 | 2 | 1 | 4 | 4 | 4 | 3 | 2 | 3 | 2 | 1 | 9 | 4 | 1 | 3 | 3 |
| 1087 | 2 | 2 | 2 | 2 | 2 | 1 | 4 | 4 | 4 | 4 | 4 | 5 | 3 | 4 | 4 | 5 | 5 | 5 | 4 | 5 | 5 | 5 | 1 | 9 | 1 | 3 | 0 | 0 |
